# Design, synthesis, and cytotoxic activities of isaindigotone derivatives as potential anti-gastric cancer agents

**DOI:** 10.1080/14756366.2022.2065672

**Published:** 2022-04-21

**Authors:** Kangjia Du, Wantong Ma, Chengjie Yang, Zhongkun Zhou, Shujian Hu, Yanan Tian, Hao Zhang, Yunhao Ma, Xinrong Jiang, Hongmei Zhu, Huanxiang Liu, Peng Chen, Yingqian Liu

**Affiliations:** School of Pharmacy, Lanzhou University, Lanzhou, China

**Keywords:** Isaindigotone, cytotoxicity, MMP, apoptosis, PI3K/AKT/mTOR

## Abstract

A series of novel derivatives of isaindigotone, which comes from the root of *isaits indinatca* Fort, were synthesised (Compound **1**–**26**). Four human gastrointestinal cancer cells (HCT116, PANC-1, SMMC-7721, and AGS) were employed to evaluate the anti-proliferative activity. Among them, Compound **6** displayed the most effective inhibitory activity on AGS cells with an IC_50_ (50% inhibitory concentration) value of 2.2 μM. The potential mechanism study suggested that Compound **6** induced apoptosis in AGS cells. The collapse of mitochondrial membrane potential (MMP) in AGS cells was proved. In docking analysis, good affinity interaction between Compound **6** and AKT1 was discovered. Treatment of AGS cells with Compound **6** also resulted in significant suppression of PI3K/AKT/mTOR signal pathway. The collapse of MMP and suppression of PI3K/AKT/mTOR signal pathway may be responsible for induction of apoptosis. This derivative Compound **6** could be useful as an underlying anti-tumour agent for treatment of gastric cancer.

## Graphical Abstract

**Figure:**
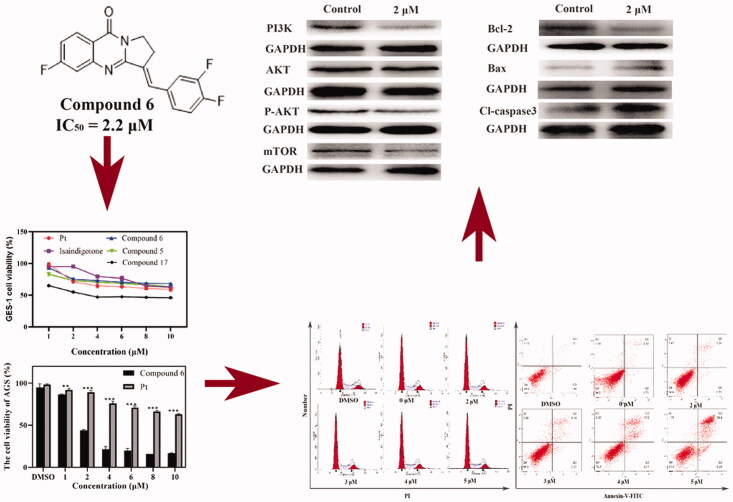

## Introduction

Gastric cancer (GC) is the fourth leading cause of death in the world and is well-known for its high mortality rate^1^. Advanced GC treatment still needs research for more effective drugs and regimens with lower adverse reactions^2^. Therefore, novel cytotoxic agents with higher therapeutic effects and lower toxicity are wanted urgently. It is generally acknowledged that PI3K/AKT/mTOR signal pathway plays significant roles in the regulation of cellular proliferation, migration, and apoptosis^3–5^. As one of the most frequent mutationally altered pathways in GCs, PI3K/AKT/mTOR signal pathway is hyperactive in 47% of GCs^6–9^. Many approved PI3K/AKT/mTOR pathway targeting compounds can suppress proliferation and induce apoptosis, which suggests that inhibition of PI3K/AKT/mTOR pathway could achieve good treatment for GC^10–14^.

Natural products have increasingly aroused researchers’ attention in anti-cancer drug discovery for their unique chemical diversities^15^. It was reported that in the past four decades, nearly 62% anti-cancer drugs were directly or indirectly derived from natural products^16^. The rich resources of Traditional Chinese Medicine accumulated through long-term research and clinical treatment provide a rich source for further research and development of anti-tumour drugs.

In traditional Chinese medical practice, *Isatis indigotica* Fortune is commonly used in the treatment of epidemic hepatitis and epidemic encephalitis^17^. Current research also indicated that the extracts from dried leaves and roots of *I. indigotica* has a distinguished anti-tumour activity^18^. As a naturally occurring alkaloid, isaindigotone (**1**) was isolated from the root of *isaits indinatca* Fort^19^. The structure of Isaindigotone comprised a pyrrolo [*2, 1*-*b*] quinazoline moiety conjugated with a benzylidene group^20^. The previous study discovered that the modified isaindigotone derivatives can be a class of highly selective ligands for telomeric G-quardruplex^21^. Chan’s research further implied that isaindigotone derivatives can downregulate *c-myc* transcription through disrupting the interaction of G-quardruplex and NM23-H2^20^. Recent study indicated that multiple signal pathways, including PI3K-AKT, were involved in the herbal efficacy of *I. indigotica*^22^. Same parent structure can also be found in PI3K delta inhibitor Idelalisib^23^. All above inspired these isaindigotone derivatives may have further applications in cancer therapy strategy.

**Figure 1.**
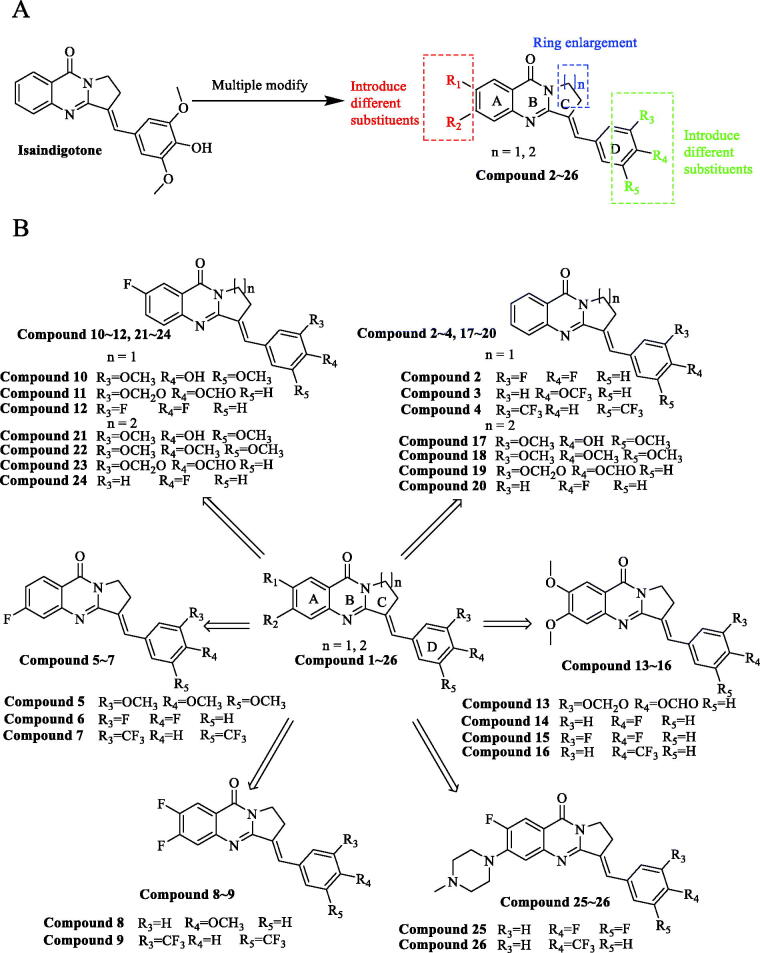
Isaindigotone and its derivatives. (A) General design strategy of the title compounds. (B) Chemical structures of Isaindigotone derivatives.

A derivative library containing 26 compounds was established to investigate the possible cytotoxic mechanism (Figure 1). The anti-proliferation effects of the analogues on human liver cancer SMMC-7721, pancreatic cancer PANC-1, and GC AGS, and colorectal cancer HCT116 cell lines were determined by 3-(4,5-Dimethylthiazol-2-yl)-2,5-diphenyltetrazolium bromide (MTT) assay. We found that Compound **6** was a highly potent inhibitor against GC. Cell cycle and apoptosis assay, Western blotting, transwell cell invasion assay was then performed to investigate the pharmacological mechanism of Compound **6**.

## Result and discussion

### Chemistry

**Scheme 1.**
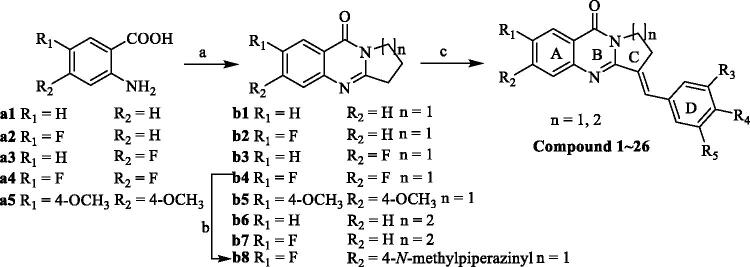
Synthesis of isaindigotone derivatives. Reagents and conditions: (A) pyrrolidin-2-one or 2-piperidone, POCl_3_, toluene, reflux; (B) 1-methylpiperazine, DMF, K_2_CO_3_, reflux; (C) different substituted benzaldehydes, AcOH, AcONa, reflux.

The synthetic methods for Compounds **1**–**27** was shown in Scheme 1. The cyclisation of a1–a5 with phosphorus oxychloride proceeded smoothly with formation of b1–b7 and the intermediate of b8 was prepared from b4 by fluorination with *N*-methylpiperazine. Then, intermediate products b1–b8 was condensed with appropriate benzaldehydes to afford the title Compounds **1**–**27**. The structures of the synthesised compounds were then confirmed by ^1^H NMR, ^13 ^C NMR, and mass spectrometry.

### Anti-proliferation activity of isaindigotone derivatives

**Figure 2.**
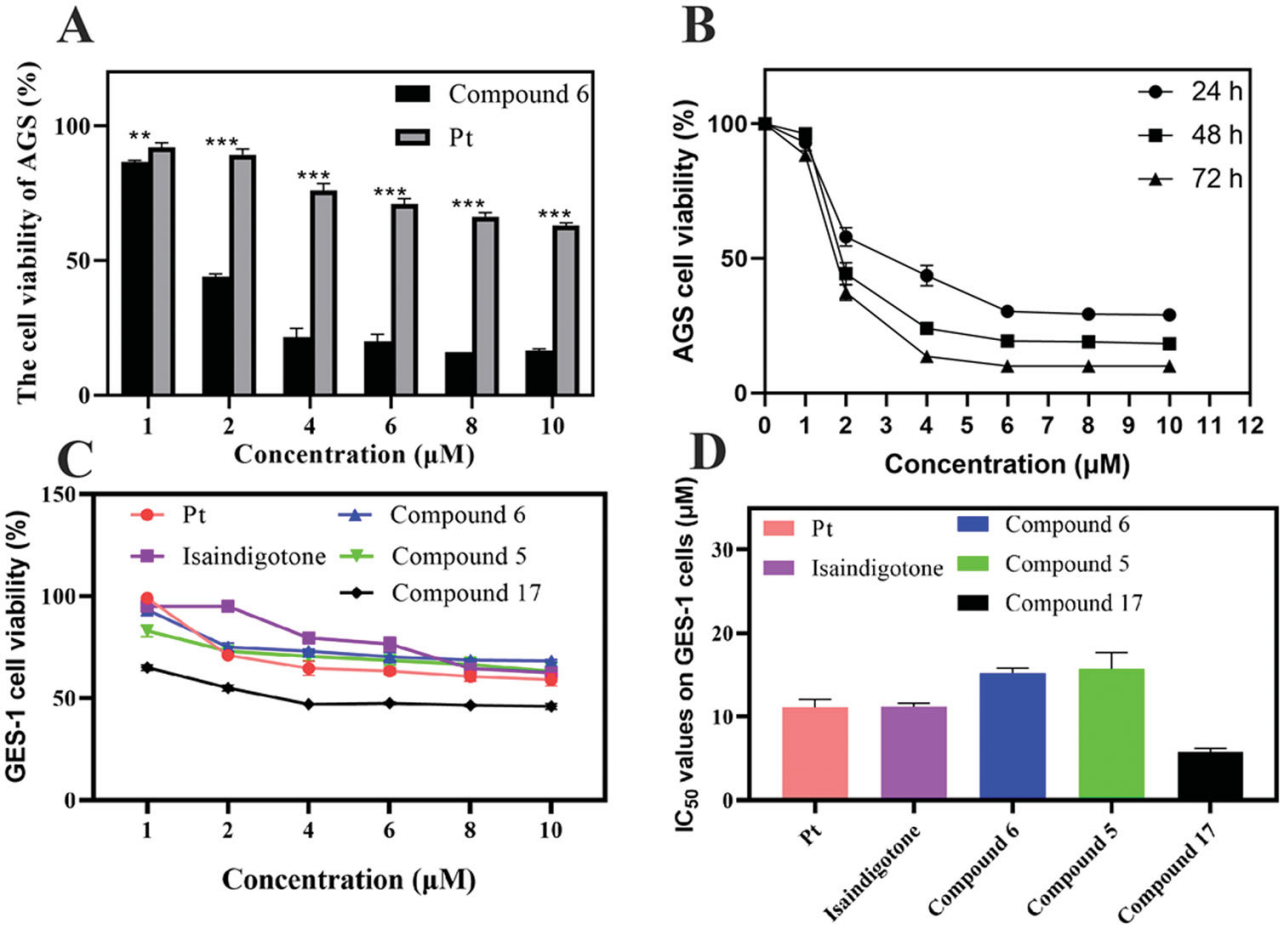
AGS and GES-1 cell viability after Compound **6** and cisplatin treatment. (A, B) Concentration and time dependent anti-proliferation effects of Compound **6** on AGS cells. (C, D) After 48 h treatment, the cytotoxic effects of the most active compounds, Isaindigotone and cisplatin on GES-1 cells. Values are shown as the means ± standard, *n* = 3. * *p*< 0.05, **p< 0.01, *** *p*< 0.001 compared with Pt.

*In vitro* cytotoxic activities of 26 isaindigotone derivatives were investigated on different human cancer cell lines including AGS, HCT116, SMMZ-7721, and PANC-1 by MTT assay. The IC_50_ values of isaindigotone derivatives and positive control cisplatin (Pt) for 48 h were presented in Table 1. Most isaindigotone derivatives showed more pronounced cytotoxicity in AGS cells (IC_50_=1.9–44.5 μM). Isaindigotone derivatives with better anti-proliferation activities on AGS cells were selected and tested on human gastric mucosa cells GES-1 (Table 1). The results showed that most of the selected derivatives showed better safety on GES-1 cells than Pt (Figure 2(C,D)).

**Table 1.** Anti-proliferative activities (IC_50_, μM) of the derivatives for 48 h.

Compounds	PANC-1	HCT116	SMMC7721	AGS
**1**	>50.00	>50.00	>50.00	7.93 ± 0.97
**2**	>50.00	>50.00	>50.00	20.71 ± 0.93
**3**	>50.00	>50.00	>50.00	>50.00
**4**	19.15 ± 2.22	>50.00	>50.00	20.47 ± 2.65
**5**	>50.00	>50.00	>50.00	1.96 ± 0.84
**6**	9.94 ± 1.57	17.95 ± 0.00	>50.00	2.23 ± 0.01
**7**	>50.00	22.01 ± 1.25	>50.00	19.62 ± 0.31
**8**	>50.00	>50.00	>50.00	44.50 ± 2.10
**9**	>50.00	>50.00	>50.00	22.32 ± 1.05
**10**	>50.00	>50.00	>50.00	13.40 ± 0.11
**11**	>50.00	>50.00	>50.00	13.29 ± 1.90
**12**	28.12 ± 1.54	>50.00	>50.00	17.62 ± 2.54
**13**	>50.00	>50.00	>50.00	>50.00
**14**	37.32 ± 2.60	>50.00	27.31 ± 1.42	18.37 ± 3.06
**15**	>50.00	>50.00	>50.00	12.76 ± 3.13
**16**	31.93 ± 1.55	>50.00	40.42 ± 1.86	9.07 ± 1.19
**17**	21.37 ± 1.52	2.80 ± 0.90	>50.00	6.79 ± 1.03
**18**	14.87 ± 0.30	25.08 ± 2.22	49.05 ± 0.80	9.50 ± 1.43
**19**	37.12 ± 2.76	>50.00	>50.00	9.11 ± 0.22
**20**	>50.00	>50.00	>50.00	>50.00
**21**	>50.00	>50.00	>50.00	43.82 ± 1.00
**22**	>50.00	>50.00	>50.00	>50.00
**23**	23.94 ± 1.82	>50.00	>50.00	20.29 ± 0.44
**24**	>50.00	>50.00	>50.00	18.74 ± 2.07
**25**	>50.00	>50.00	>50.00	13.62 ± 1.23
**26**	>50.00	>50.00	>50.00	14.22 ± 1.90
**Pt**	>50.00	>50.00	>50.00	>50.00

**Table 2.** The structure of isaindigotone derivatives.

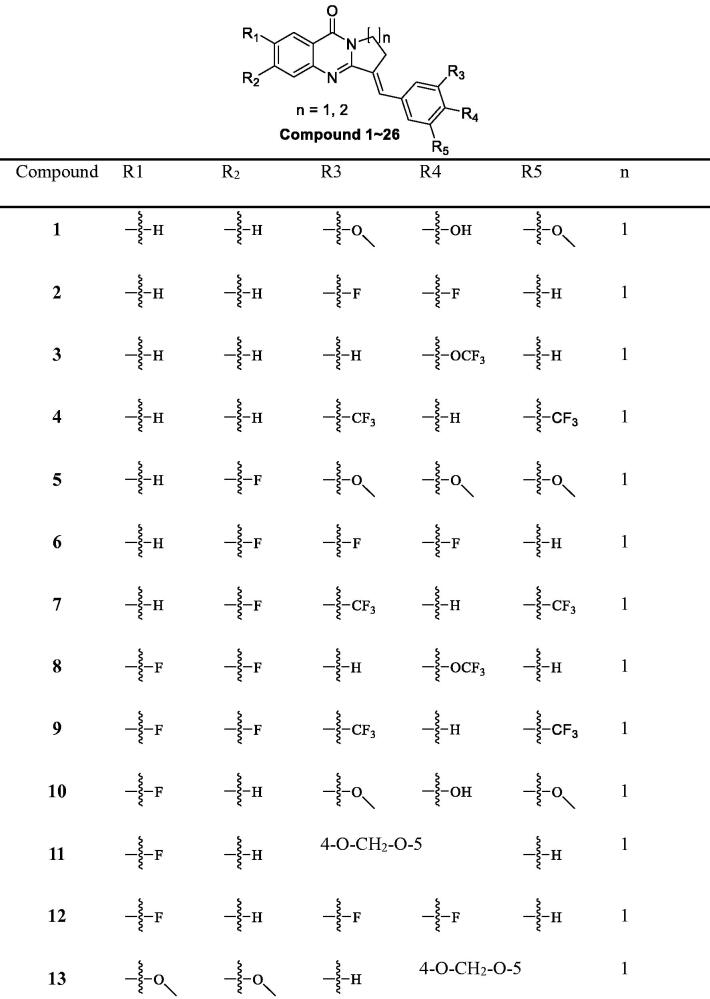	
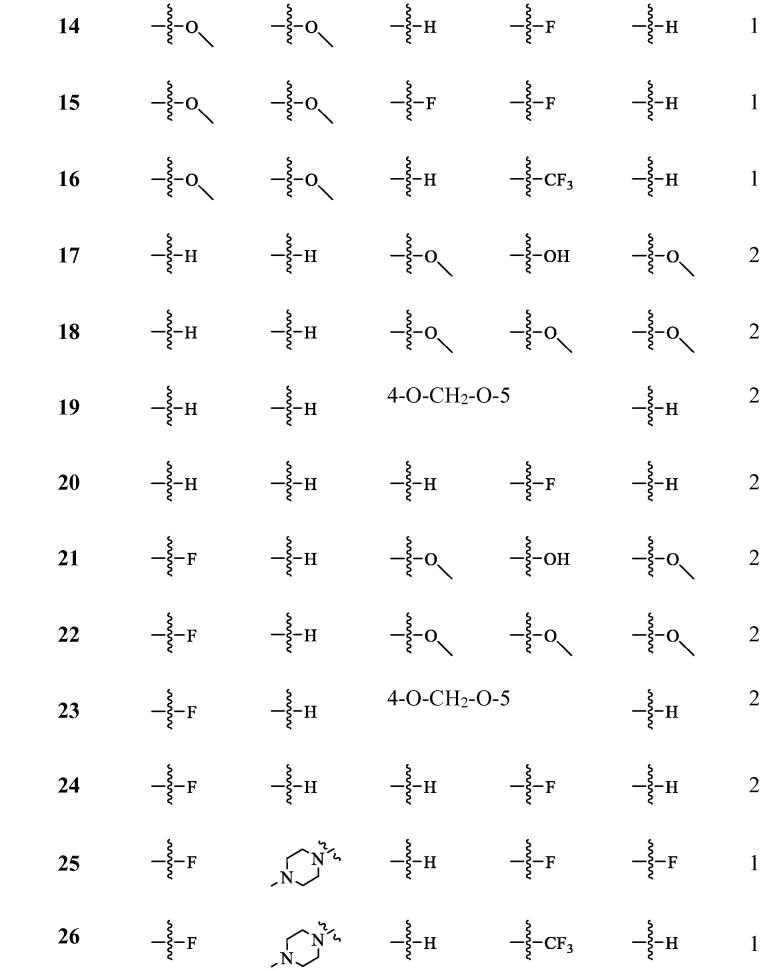	

For AGS cells, having changed ring-C from five-membered ring to six-membered ring (Figure 1(B)), and introduce tri-substituted methoxy (Compound **18**), *p*-fluorophenyl (Compound **20**), Benzo 1,3 dioxolane (Compound **19**) on the terminal benzene ring, we found that the activities of the derivatives are not improved when compared to isaindigotone (Compound **1**). Therefore, changing ring-C from five-membered ring to six-membered ring cannot help to improve isaindigotone’s anti-proliferation activity. When introduced double-substituted electron donating groups and electron withdrawing groups on R_1_ and R_2_ (Figure 1(B)), we found that the activity of the methoxy substituted derivatives (Compounds **13**–**16**) is better than that of the difluoro substituted derivatives (Compounds **8**, **9**), but both of their activities are lower than that of isaindigotone (Compound **1**). The impact of diverse substitution pattern of ring-A and ring-D was also assessed (Figure 1(B)). To determine the effects of substitutions on ring-A, Compound **10** containing halogen group (R_1_=F) was examined and displayed less inhibitory activity than isaindigotone (Compound **1**). When exploring the effects of substitutions on ring-D, we found that Compounds **10**, **11** containing electron donating groups (benzodioxole, methoxyl, and hydroxyl) have better inhibitory effects than Compound **12** containing electron withdrawing groups on phenyl ring-D. Same rule could also be found among Compounds **5**–**7**. Additionally, the effects of the same functional group at R_1_ and R_2_ position were evaluated. For example, Compound **6** containing F at position R_2_ has better anti-proliferation activity than Compound **2** (R_1_=H, R_2_=H) and Compound **12** (R_1_=F, R_2_=H). The inhibitory effects improved in the order Compound **2** (R_1_=H, R_2_=H)<Compound **12** (R_1_=F, R_2_=H)<Compound **6** (R1 = H, R2 = F). Taken together, among these isaindigotone derivatives, Compound **6** showed stronger inhibitory activities and was selected for further study to define its possible molecular mechanism.

### Cell viability assay

Cytotoxic activity testes of Compound **6** on AGS cell line at 24, 48, and 72 h were then conducted using MTT assay. As observed, the cell viability was significantly inhibited in dose- and time- dependent manner (Figure 2(A,B)). Thus, Compound **6** was selected for further mechanism studies.

#### Cell cycle analysis

**Figure 3.**
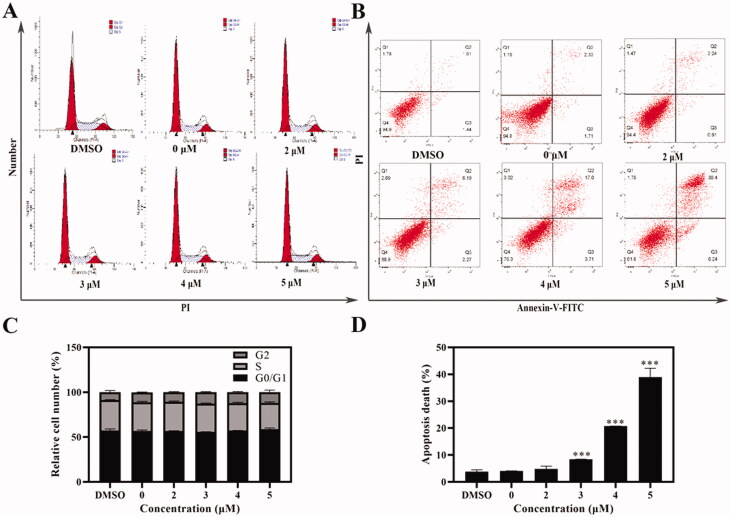
Cell cycle and apoptosis assays were tested with flow cytometry. (A, C) AGS cells were treated with Compound **6** (0.0, 2.0, 3.0, 4.0, and 5.0 μM) for 24 h, and analysed by flowed cytometry (B, D) Analysis of cell apoptosis induced by Compound **6** using Annexin V/PI assay. * *p*< 0.05, **p< 0.01, *** *p*< 0.001 compared with negative control.

As a novel isaindigotone derivative, Compound **6** may induce cell cycle arrest^20^. Thus, AGS cells were treated with Compound **6** in different concentrations for 24 h and stained with PI (DMSO was used as negative control). Cell cycle distributions were analysed with a flow cytometry. The proportions of AGS cells in different phases had little distinction when compared with negative control group (Figure 3(A,C)). Therefore, it can be concluded that the anti-proliferation effect of Compound **6** was not induced by cell cycle arrest.

### Morphological analysis

**Figure 4.**
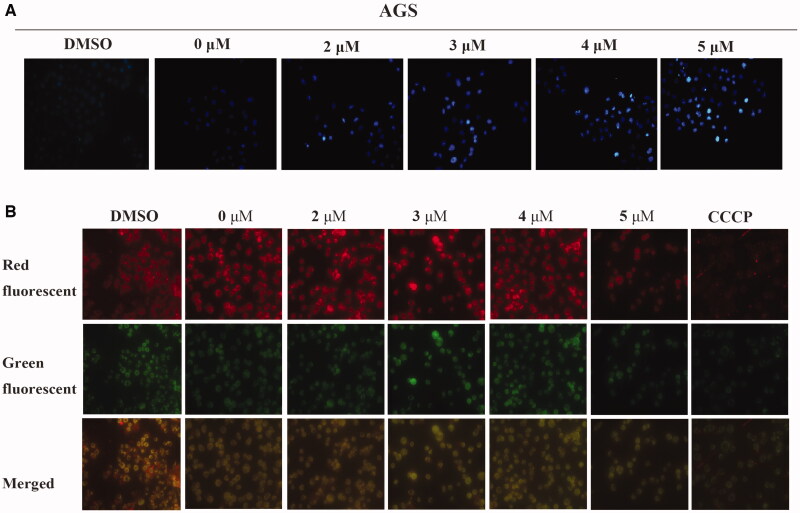
Compound **6** induced apoptosis and mitochondrial depolarisation in AGS cells. (A) AGS cells were treated with Compound **6** (0.0, 2.0, 3.0, 4.0, and 5.0 μM) for 48 h and stained with Hoechst 33258. DMSO was used as negative control. (A) AGS cells were treated with Compound **6** (0.0, 2.0, 3.0, 4.0, and 5.0 μM), CCCP (5.0 μM) and DMSO for 48 h and MMP was determined using the JC-10 kit.

To further determine whether the growth inhibition was caused by apoptosis, Hoechst 33258 staining was used to visualise nuclear morphology^24^. Fluorescence microscopy examination revealed that the treated AGS cells showed typical morphological changes such as chromatin condensation and cell shrinkage (Figure 4(A)). This result indicates that inducing apoptosis could be the predominant mechanism of Compound **6** in anti-proliferation activity.

### Apoptosis analysis

Due to the significant morphological changes and high death rate of AGS caused by Compound **6**, an Annexin-V/PI staining was performed to further determine whether Compound **6** could induce apoptosis^25^. The translocation of phosphatidylserine in apoptotic cells makes it possible for Annexin V-FITC, a fluorescein active dye, to bind with. PI can penetrate the broken membrane and bind with DNA. Therefore, Annexin-V/PI staining can distinct live cells from apoptotic cells and necrotic cells. The result showed that the percentage of early apoptotic cells increased from 0.9% to 6.2% and the late apoptotic ratio varies from 3.2% to 30.4% (Figure 3(B,D)). The variation of the early apoptotic cells and late apoptotic cells were both in a dose-dependent manner, which indicated that Compound **6** could induce apoptosis in AGS cells.

### Mitochondrial membrane potential (MMP) analysis

It has been widely acknowledged that the loss of MMP is an early sign of mitochondrial dysfunction in apoptotic cells^26^. So, the effects of Compound **6** on MMP in AGS cells were then assessed. As shown in Figure 4(B), CCCP was used as positive control, when AGS cells were treated with different concentrations of Compound **6,** apparent loss of MMP was observed in a dose-dependent manner. The changes of MMP were detected by fluorescence microscopy using JC-10 dye. The experimental results indicated that the apoptosis of AGS cells induced by Compound **6** was associated with mitochondrial-mediated pathways. The effects of Compound **6** on activation of mitochondrial-dependent pathway related factors were then examined, Bax and Bcl-2 and apoptosis effectors caspase-3 by Western blot (WB) assays. As shown in Figure 6(B,D) after treating with Compound **6** for 48 h, the protein expression levels of Bax and cleaved-caspase-3 increased significantly and the protein expression level of Bcl-2 decreased. These results indicated that the apoptosis of AGS cells was affected by Compound **6** through mitochondrial-related pathways.

#### Molecular docking study

**Figure 5.**
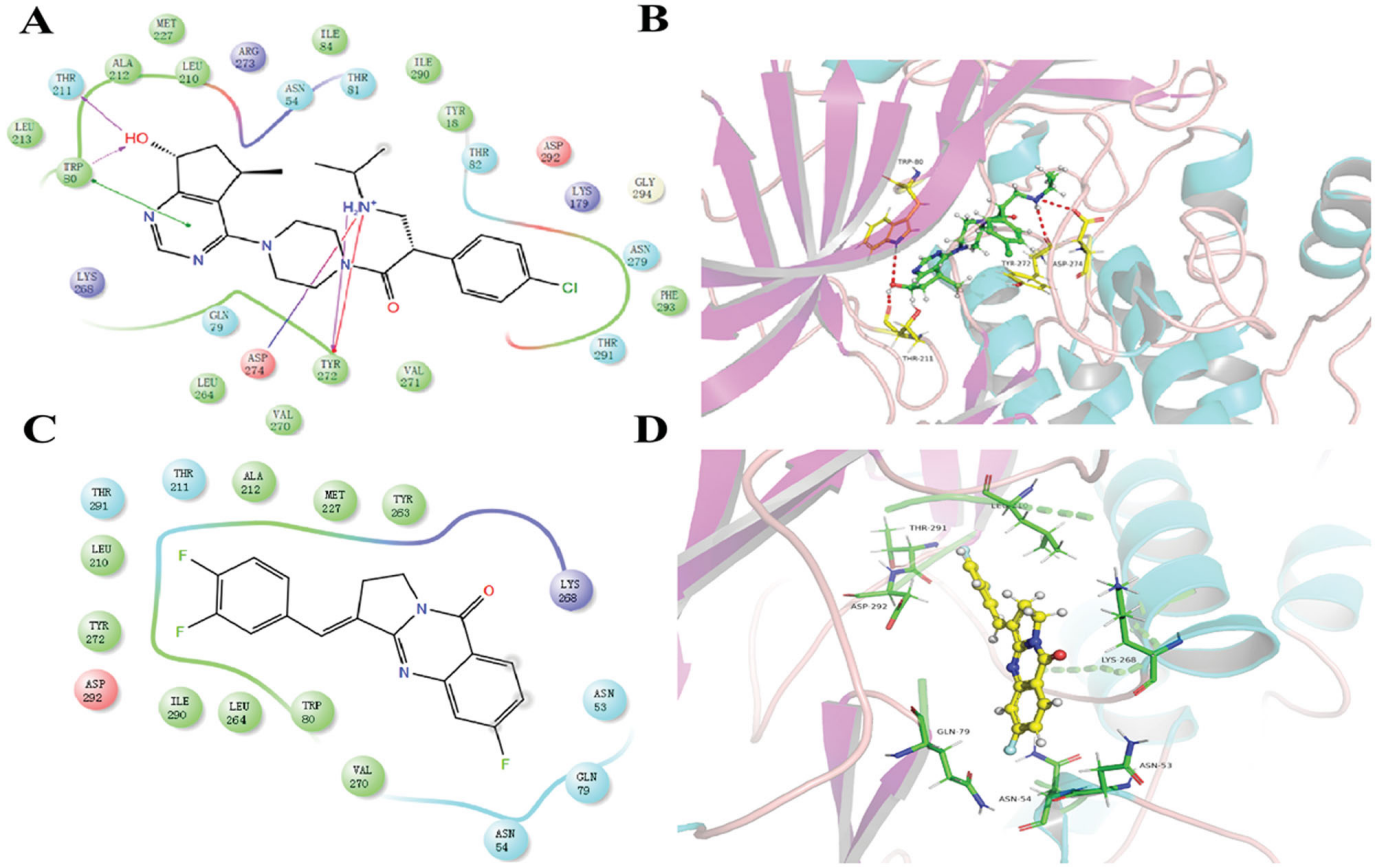
The result of molecular docking of Compound **6** and ipatasertib with AKT1 (PDB: 6HHF). (A, B) The binding conformation of ipatasertib in the active site of AKT1. (C, D) The binding conformation of Compound **6** in the active site of AKT1.

Molecular docking analysis was made between Compound **6** and PI3Kα (PDB: 6gvf), AKT1 (PDB: 6hhf), mTOR (PDB: 4jt6), respectively (Supplementary material). Better affinity interaction between Compound **6** and AKT1 was discovered. We also compared the binding model between Compound **6** and AKT inhibitor ipatasertib (Figure 5). Both of them were able to fit into the same active site. For AKT1 protein, the carboxyl anion on Asp 292 formed a negative interaction with Compound **6** and ipatasertib. Lys 268 could form a positive interaction with both of them due to its amino cation. Asn 54 and Glu 79 as polar amino acids formed strong polar interactions with them. However, ipatasertib could also form hydrogen bonds with Tyr 272, Asp 274, Thr 211, and Trp 80. These docking results provided crucial insights into the protein-ligand interactions and further structural modification for activity improvement.

### WB analysis

**Figure 6.**
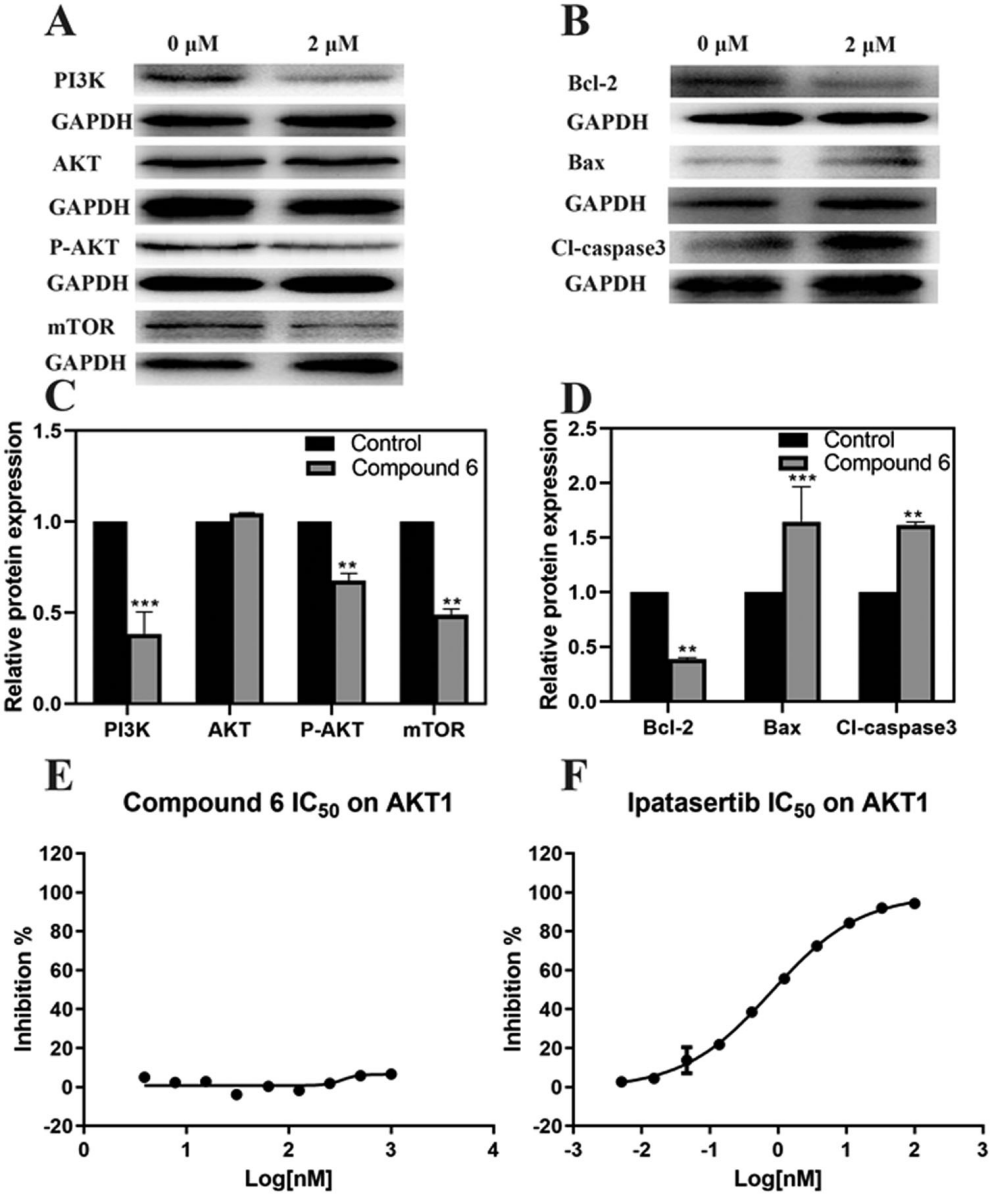
The effect of Compound **6** and the expression of mitochondrial-dependent pathway related proteins and PI3K/AKT/mTOR signal pathway related proteins and AKT1 inhibition effects of Compound **6** and positive control ipatasertib. (A, B) protein levels of PI3K, AKT, p-AKT, mTOR, Bcl-2, Bax, and cleaved-caspase 3. (C, D) Statistical analysis. (E) Inhibition effects of Compound **6**. (F) Inhibition effects of Ipatasertib. * *p*< 0.05, **p< 0.01, *** *p*< 0.001 compared with control.

PI3K/AKT/mTOR signalling pathway was reported to have connection with apoptosis^27–29^. So, WB assay was performed to evaluate whether Compound **6** had affected this signal pathway. As is shown in Figure 6(A,C), Compound **6** can significantly reduce the phosphorylation of AKT. Furthermore, Compound **6** down-regulated the expression of PI3KCA and mTOR proteins in AGS cell lines, which suggested that the function of PI3K/AKT/mTOR signal pathway can be influenced by Compound **6**.

### *In vitro* AKT1 inhibition assay

To further confirm whether Compound **6** can block AKT1 kinase, *in vitro* enzyme assay was performed (Figure 6(E,F)). The results indicated that Compound **6** showed miner inhibitory effects on AKT1 kinase when compared with positive control ipatasertib.

### Cell migration analysis

**Figure 7.**
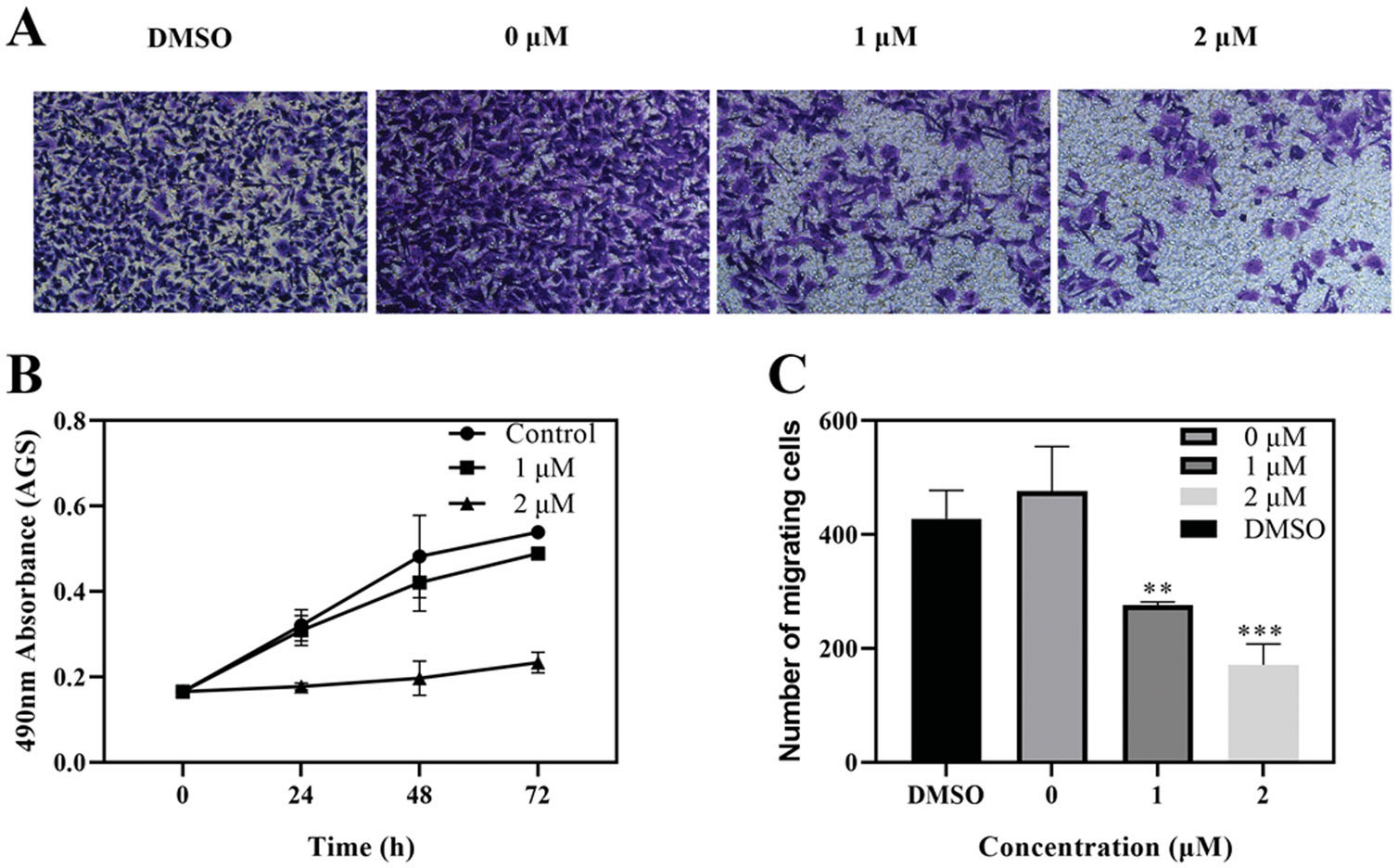
Effects of Compound **6** on tumour cell migration. (A) Transwell assay showed that cell migration of AGS were inhibited by Compound **6**. (B) MTT assay showed that Compound **6** (0.0, 1.0, and 2.0 μM) inhibited AGS cell proliferation at 24, 48, and 72 h. (C) Statistic analysis. Values are shown as the means ± standard, *n* = 3. * *p*< 0.05, **p< 0.01, *** *p*< 0.001 compared with negative control (DMSO).

Tumour metastatic ability is an important characteristic of tumourigenesis, which can be reflected by cell migration *in vitro*^30^. PI3K/AKT/mTOR signal pathway has been reported to connect with cancer invasion and metastasis^31^. Thus, we evaluated the ability of Compound **6** inhibiting cell migration with transwell migration assay. As shown in Figure 7, when compared with negative control group, Compound **6** can significantly reduce the number of cells, which migrated to the bottom layer of the chamber. The result indicated that Compound **6** prevented the cell migration in a dose-dependent manner.

## Conclusion

MTT assay indicated that the most active Compound **6** had better cytotoxic activity than Pt and less cytotoxic effect on human gastric mucosa cells. Cell migration experiment verified that Compound **6** can inhibit cell proliferation. Hoechst stain indicated that Compound **6** might induce the apoptosis of AGS cell line, which was further confirmed with flow cytometry analysis. However, the results showed that Compound **6** could not cause cell cycle arrest in AGS cell line. Through docking analysis, good binding affinity between kinase AKT1 and Compound **6** was found, which suggested that Compound **6** may affect PI3K/AKT signalling pathway. The results showed that a significant decrease in expression of PI3KCA, p-AKT, and mTOR after treating with Compound **6**. Through MMP assay we found that Compound **6** did cause the loss of MMP, which was consistent with the results of WB that Compound **6** activated Bax and reduced Bcl-2 expression.

*I. indigotica* was widely used in China for alleviating inflammation^32^, and many research focussed on its anti-inflammatory activities including attenuating pyrexia, inhibiting the writhing response and paw oedema of mice^33^, and inhibiting interleukin-6 and tumour necrosis factor-α production^34^. Many studies also demonstrated that anti-inflammatory drugs have anti-proliferation effects which indicated us to explore the chemotherapeutic activities of the extraction of *I*. *indigotica* for its significant anti-inflammatory effects^35^^,^^36^. Different from the previous studies of the constituents identified in *I. indigotica,* which induced apoptosis through regulating caspase-3/Fas antigen pathway and NF-κB pathway^37^^,^^38^, our research discovered that one of the constituents, isaindigotone, and its derivatives have distinct anti-proliferation activities on AGS cell line and can affect PI3K/AKT/mTOR pathway.

Further specific studies on determining how Compound **6** affects PI3K signal pathway will be the priority in subsequent work. Thus, we believe that Compound **6** will provide a choice for GC treatment and hope our work will inspire other researches to focus more attention on *isaits indinatca* Fortune’s potential anti-cancer activities.

## Experimental section

### General experimental procedures

Chemical reagents and solvent required during the synthesis process were purchased from Energy Chemical and were used and purified according to standard methods. Bruker AM-400 (Bruker Company, USA) spectrometer was employed to obtain ^1^H NMR (400 MHz) and ^13 ^C NMR (100 MHz) spectra of new derivatives and TMS was used as reference. ZAB-HS and Bruker Daltonics APEXII49e instruments were used to obtain electrospray ionisation mass spectrometry. A Kofler melting point apparatus was used to detect melting points of the new derivatives. The purity of synthesised compound was confirmed by high performance liquid chromatography (VARIAN, ProStar) equipped with an Ultimate XB-C18 column and eluted with methanol-water containing 0.1% TFA at a flow rate of 1 ml/min.

### Chemical synthesis

#### Synthetic procedure for target Compound b1–b7

Initially, POCl_3_ (45.0 ml) was carefully added to a solution of various 2-aminobenzoic acid (19.3 mmol, 1 eq) and pyrrolidine-*2*-one or 2-piperidone (38.6 mmol, 2 eq) at room temperature. The mixture was then stirred at 110 °C for 7 h. After POCl_3_ was removed under reduced pressure, the residue was poured into ice water, and then a solution of NaOH was added to make the solution basic. The mixture was extracted with 3 × 50 ml portions of CH_2_Cl_2_. The combined organic phase was dried over MgSO_4_ and was concentrated *in vacuo* and then purified by flash column eluted with petroleum ether/EtOAc (4:1) to afford a white solid.

#### Synthetic procedure for target Compound b8

A catalytic amount of Na_2_CO_3_ was added to a DMF (36.0 ml) solution of intermediate b4 (15.0 mmol) and N-methylpiperazine (4.0 ml). The mixture was then stirred at 140 °C for 2 h. After DMF was partly removed under reduced pressure, the residue was poured into 30.0 ml of water, and then filtered and washed with water to afford a white solid.

#### Synthetic procedure for target Compound 1–26

A catalytic amount of NaOAc was added to an AcOH (70.0 ml) solution of intermediates b1–b8 (10.0 mmol) and different substituted benzaldehydes (20.0 mmol). The mixture was then stirred at 115 °C for 6 h. After AcOH was partly removed under reduced pressure, the residue was poured into 15.0 ml of ice-cold acetone and then filtered and washed with acetone to afford a white or yellow solid (Table 2). The spectral date of Compounds **1–26** are given below.

##### (E)-3–(4-hydroxy-3,5-dimethoxybenzylidene)-2,3-dihydropyrrolo[2,1-b]quinazolin-9(1H)-one (1)

Yield: 80%; yellow solid; ^1^H NMR (400 MHz, CDCl_3_) *δ*: 8.30 (d, *J* = 7.9 Hz, 1H), 7.78 (s, 1H), 7.74 (m, 2H), 7.44 (m, 1H), 6.84 (s, 2H), 5.79 (s, 1H), 4.46–4.21 (t, *J* = 7.2 Hz, 2H), 3.96 (s, 6H), 3.32 (t, *J* = 6.1 Hz, 2H), 1.59 (s, 3H). ^13 ^C NMR (100 MHz, CDCl_3_) *δ*: 161.31, 155.77, 149.81, 147.18, 136.21, 134.24, 131.14, 128.98, 127.16, 127.14, 126.00, 120.81, 107.10, 56.40, 44.04, 25.45. Purity 95.5% by HPLC. HRESIMS *m/z* 351.1267 [M + H]^+^ (calcd for C_20_H_18_N_2_O_4_, 351.1284).

##### (E)-3-(3,4-difluorobenzylidene)-2,3-dihydropyrrolo[2,1-b]quinazolin-9(1H)-one(2)

Yield: 69%; yellow solid; ^1^H NMR (400 MHz, CDCl_3_) *δ*: 8.29 (d, *J* = 7.9 Hz, 1H), 7.87–7.63 (m, 3H), 7.45 (m, 1H), 7.37 (m, 1H), 7.27 (m, 2H), 4.31 (t, *J* = 7.2 Hz, 2H), 3.26 (t, *J* = 7.4 Hz, 2H). ^13 ^C NMR (100 MHz, CDCl_3_) *δ*: 161.11, 155.03, 151.66 (d, *J* = 13.0 Hz), 149.54, 149.19 (d, *J* = 12.8 Hz), 134.32, 132.56 (d, *J* = 2.8 Hz), 128.37 (d, *J* = 2.3 Hz), 127.34, 126.44, 126.41, 120.95, 118.12, 117.94, 117.92, 117.74, 44.00, 25.34. Purity 95.2% by HPLC. HRESIMS *m/z* 311.0918 [M + H]^+^ (calcd for C_18_H_12_F_2_N_2_O, 311.0985).

##### (E)-3–(4-(trifluoromethoxy)benzylidene)-2,3-dihydropyrrolo[2,1-b]quinazolin-9(1H)-one (3)

Yield: 73%; yellow solid; ^1^H NMR (400 MHz, CDCl_3_) *δ*: 8.29 (d, *J* = 8.0 Hz, 1H), 7.82 (d, *J* = 3.0 Hz, 1H), 7.75 (d, *J* = 4.1 Hz, 2H), 7.58 (d, *J* = 8.4 Hz, 2H), 7.44 (m, 1H), 7.36–7.12 (m, 2H), 4.30 (t, *J* = 7.2 Hz, 2H), 3.27 (t, *J* = 7.4, 2H). ^13 ^C NMR (100 MHz, CDCl_3_) *δ*: 161.15, 155.19, 149.59, 149.26, 134.29, 134.10, 132.48, 131.10, 128.97, 127.33, 126.43, 126.34, 121.12, 120.95, 44.00, 25.44. Purity 98.2% by HPLC. HRESIMS *m/z* 359.0929 [M + H]^+^ (calcd for C_19_H_13_F_3_N_2_O_2_, 359.0986).

##### (E)-3–(3,5-bis(trifluoromethyl)benzylidene)-2,3-dihydropyrrolo[2,1-b]quinazolin-9(1H)-one (4)

Yield: 76%; yellow solid; ^1^H NMR (400 MHz, CDCl_3_) *δ*: 8.33 (d, *J* = 8.0 Hz, 1H), 7.98 (s, 2H), 7.93 (s, 1H), 7.87 (s, 1H), 7.78 (d, *J* = 2.1 Hz, 2H), 7.50 (m, 1H), 4.36 (t, *J* = 7.1 Hz, 2H), 3.35 (t, *J* = 7.5 Hz, 2H). ^13 ^C NMR (100 MHz, CDCl_3_) *δ*: 161.05, 137.46, 135.82, 134.46, 132.58, 132.24, 129.03, 129.01, 127.57, 127.18, 126.81, 126.51, 122.06, 121.73, 121.11, 44.01, 25.46. Purity 99.0% by HPLC. HRESIMS *m/z* 411.0854 [M + H]^+^ (calcd for C_20_H_12_F_6_N_2_O, 411.0877).

##### (E)-6-fluoro-3-(3,4,5-trimethoxybenzylidene)-2,3-dihydropyrrolo[2,1-b]quinazolin-9(1H)-one (5)

Yield: 85%; yellow solid; ^1^H NMR (400 MHz, CDCl_3_) *δ*: 8.28 (dd, *J* = 8.8, 6.2 Hz, 1H), 7.77 (d, *J* = 2.9 Hz, 1H), 7.36 (dd, *J* = 9.9, 2.5 Hz, 1H), 7.14 (m, 1H), 6.80 (s, 2H), 4.30 (t, *J* = 7.3 Hz, 2H), 3.92 (d, *J* = 2.4 Hz, 9H), 3.38 (t, *J* = 7.3 Hz, 2H).^13^C NMR (100 MHz, CDCl_3_) *δ*: 160.54, 156.77, 153.38, 139.40, 131.54, 130.85, 130.14, 128.96 (d, *J* = 10.7 Hz), 117.58, 114.87 (d, *J* = 8.4 Hz), 114.68, 112.36 (d, *J* = 21.7 Hz), 112.07, 107.41, 61.01, 56.30, 44.07, 25.42. Purity 94.0% by HPLC. HRESIMS *m/z* 383.1329 [M + H]^+^ (calcd for C_21_H_19_FN_2_O_4_, 383.1378).

##### (E)-3-(3,4-difluorobenzylidene)-6-fluoro-2,3-dihydropyrrolo[2,1-b]quinazolin-9(1H)-one (6)

Yield: 83%; yellow solid; ^1^H NMR (400 MHz, CDCl_3_) *δ*: 8.31 (dd, *J* = 8.8, 6.2 Hz, 1H), 7.77 (d, *J* = 2.6 Hz, 1H), 7.38 (dd, *J* = 9.7, 2.5 Hz, 1H), 7.31 (m, 1H), 7.26 (m, 1H), 7.17 (m, 1H), 4.31 (t, *J* = 7.3 Hz, 2H), 3.29 (t, *J* = 7.3 Hz, 2H).^13^C NMR (100 MHz, CDCl_3_) *δ*: 160.45, 158.60, 156.27, 151.84 (d, *J* = 13.6 Hz), 151.65, 129.09 (d, *J* = 4.5 Hz), 128.96, 126.53, 126.47, 118.12 (d, *J* = 18.8 Hz), 117.98, 117.75 (d, *J* = 9.9 Hz), 115.24, 115.01, 112.66, 112.44, 44.04, 25.34. Purity 100.0% by HPLC. HRESIMS *m/z* 329.0823 [M + H]^+^ (calcd for C_18_H_11_F_3_N_2_O, 329.0700).

##### (E)-3-(3,5-bis(trifluoromethyl)benzylidene)-6-fluoro-2,3-dihydropyrrolo[2,1-b]quinazolin-9(1H)-one (7)

Yield: 80%; yellow solid; ^1^H NMR (400 MHz, CDCl_3_) *δ*: 8.31 (dd, *J* = 8.8, 6.1 Hz, 1H), 7.98 (s, 2H), 7.92 (t, *J* = 3.0 Hz, 1H), 7.88 (s, 1H), 7.39 (dd, *J* = 9.7, 2.5 Hz, 1H), 7.19 (t, *J* = 8.5 Hz, 1H), 4.35 (t, *J* = 7.1 Hz, 2H), 3.35 (t, J = 7.2 Hz, 2H). ^13 ^C NMR (100 MHz, CDCl_3_) *δ*: 167.85, 165.32, 160.33, 155.61, 151.66 (d, *J* = 13.1 Hz), 137.27, 135.48, 132.45 (d, *J* = 33.6 Hz), 129.57–128.55 (m), 127.86, 124.41, 122.23 (d, *J* = 4.3 Hz), 121.70, 117.82, 115.51 (d, *J* = 23.7 Hz), 112.75 (d, *J* = 22.0 Hz), 44.06, 25.43. Purity 98.2% by HPLC. HRESIMS *m/z* 429.0760 [M + H]^+^ (calcd for C_20_H_11_F_7_N_2_O, 429.0565).

##### (E)-6,7-difluoro-3–(4-(trifluoromethoxy)benzylidene)-2,3-dihydropyrrolo[2,1-b]quinazolin-9(1H)-one (8)

Yield: 72%; yellow solid; ^1^H NMR (400 MHz, CDCl_3_) *δ*: 8.04 (dd, *J* = 10.0, 8.5 Hz, 1H), 7.81 (t, *J* = 2.9 Hz, 1H), 7.68–7.55 (m, 2H), 7.51 (dd, *J* = 10.9, 7.0 Hz, 1H), 7.31 (d, *J* = 8.3 Hz, 2H), 4.63–4.07 (m, 2H), 3.30 (t, J = 7.3 Hz, 2H). ^13 ^C NMR (100 MHz, CDCl_3_) *δ*: 159.78, 156.27, 155.98, 149.44, 133.84, 131.92, 131.19, 129.72, 121.69, 121.17, 117.81, 115.01, 114.84, 113.86, 113.68, 44.17, 25.39. Purity 98.6% by HPLC. HRESIMS *m/z* 395.0741 [M + H]^+^ (calcd for C_19_H_11_F_5_N_2_O_2_, 395.0612).

##### (E)-3–(3,5-bis(trifluoromethyl)benzylidene)-6,7-difluoro-2,3-dihydropyrrolo[2,1-b]quinazolin-9(1H)-one (9)

Yield: 79%; yellow solid; ^1^H NMR (400 MHz, CDCl_3_) *δ*: 1H NMR (400 MHz, Chloroform-d) δ 8.06 (dd, *J* = 10.0, 8.5 Hz, 1H), 7.98 (s, 2H), 7.89 (s, 1H), 7.52 (dd, *J* = 10.8, 7.0 Hz, 1H), 4.35 (t, *J* = 7.1 Hz, 2H), 3.36 (t, *J* = 7.3 Hz, 2H). ^13 ^C NMR (100 MHz, CDCl_3_) *δ*: 159.66, 156.26 (d, *J* = 14.8 Hz), 155.15 (d, *J* = 2.5 Hz), 153.70 (d, *J* = 14.6 Hz), 150.92 (d, *J* = 14.4 Hz), 148.41 (d, *J* = 14.4 Hz), 147.23 (d, *J* = 9.7 Hz), 137.20, 135.25, 132.48 (d, *J* = 33.6 Hz), 129.06, 127.86, 124.40, 122.57–121.98 (m), 121.69, 118.01 (d, *J* = 6.4 Hz), 115.17 (d, *J* = 17.9 Hz), 113.87 (dd, *J* = 19.3, 2.5 Hz), 44.17, 25.39. Purity 99.5% by HPLC. HRESIMS *m/z* 447.0665 [M + H]^+^ (calcd for C_20_H_10_F_8_N_2_O, 447.0503).

##### (E)-7-fluoro-3-(4-hydroxy-3,5-dimethoxybenzylidene)-2,3-dihydropyrrolo[2,1-b]quinazolin-9(1H)-one (10)

Yield: 85%; yellow solid; ^1^H NMR (400 MHz, CDCl_3_) *δ*: 7.91 (dd, *J* = 8.5, 3.0 Hz, 1H), 7.82–7.61 (m, 2H), 7.45 (m, 1H), 6.82 (s, 2H), 4.30 (t, *J* = 7.4 Hz, 2H), 3.95 (s, 6H), 3.47–3.21 (m, 2H). ^13 ^C NMR (100 MHz, CDCl_3_) *δ*: 161.69, 160.56, 159.23, 155.27, 147.20, 146.46, 136.29, 131.11, 129.29, 128.64, 127.03, 122.79 (d, *J* = 24.2 Hz), 121.90 (d, *J* = 8.6 Hz), 111.29 (d, *J* = 23.7 Hz), 107.10, 56.39, 44.08, 25.43. Purity 99.0% by HPLC. HRESIMS *m/z* 369.1172 [M + H]^+^ (calcd for C_20_H_17_FN_2_O_4_, 369.1040).

##### (E)-3-(benzo[d][1,3]dioxol-5-ylmethylene)-7-fluoro-2,3-dihydropyrrolo[2,1-b]quinazolin-9(1H)-one (11)

Yield: 78%; yellow solid; ^1^H NMR (400 MHz, CDCl_3_) *δ*: 7.92 (dd, *J* = 8.5, 3.0 Hz, 1H), 7.78–7.65 (m, 2H), 7.59–7.39 (m, 2H), 7.09 (d, *J* = 8.5 Hz, 1H), 6.89 (d, *J* = 7.5 Hz, 1H), 6.04 (s, 2H), 4.29 (t, *J* = 7.4 Hz, 2H), 3.34–3.20 (m, 1H). ^13 ^C NMR (100 MHz, CDCl_3_) *δ*: 160.53, 148.32 (d, *J* = 20.7 Hz), 130.57, 129.40 (d, *J* = 8.2 Hz), 129.01 (d, *J* = 7.3 Hz), 125.40, 123.67, 122.90, 111.29 (d, *J* = 23.6 Hz), 110.05, 109.01, 108.80, 108.04, 107.73, 101.57, 101.01, 56.05, 44.08, 25.51. Purity 67.5% by HPLC. HRESIMS *m/z* 337.0910 [M + H]^+^ (calcd for C_19_H_13_FN_2_O_3_, 337.0950).

##### (E)-3–(3,4-difluorobenzylidene)-7-fluoro-2,3-dihydropyrrolo[2,1-b]quinazolin-9(1H)-one (12)

Yield: 84%; yellow solid; ^1^H NMR (400 MHz, CDCl_3_) *δ*: 7.91 (dd, *J* = 8.5, 3.0 Hz, 1H), 7.81–7.66 (m, 2H), 7.47 (m, 1H), 7.37 (dd, *J* = 10.9, 7.6 Hz, 1H), 7.27 (dd, *J* = 13.4, 10.0 Hz, 2H), 4.31 (t, *J* = 7.2 Hz, 2H), 3.27 (t, *J* = 7.4 Hz, 2H). ^13 ^C NMR (100 MHz, CDCl_3_) *δ*: 161.93, 160.37 (d, *J* = 3.5 Hz), 159.46, 154.53, 146.21, 132.52 (d, *J* = 5.6 Hz), 132.25, 129.59 (d, *J* = 8.1 Hz), 128.39, 126.36 (dd, *J* = 6.4, 3.5 Hz), 122.90 (d, *J* = 24.3 Hz), 122.13 (d, *J* = 8.6 Hz), 118.13, 117.95, 117.78, 111.37 (d, *J* = 23.6 Hz), 44.04, 25.35. Purity 97.2% by HPLC. HRESIMS *m/z* 329.0823 [M + H]^+^ (calcd for C_18_H_11_F_3_N_2_O, 329.0889).

##### (E)-3-(benzo[d][1,3]dioxol-5-ylmethylene)-6,7-dimethoxy-2,3-dihydropyrrolo[2,1-b]quinazolin-9(1H)-one (13)

Yield: 72%; yellow solid; ^1^H NMR (400 MHz, CDCl_3_) *δ*: 7.60 (d, *J* = 2.9 Hz, 1H), 7.52 (s, 1H), 7.06 (s, 1H), 7.03–6.93 (m, 2H), 6.81 (d, *J* = 8.1 Hz, 1H), 5.95 (s, 2H), 4.20 (t, *J* = 7.2 Hz, 2H), 3.94 (s, 3H), 3.93 (s, 3H), 3.25–3.13 (m, 2H). ^13 ^C NMR (100 MHz, CDCl_3_) *δ*: 160.56, 154.87, 148.65, 148.19, 145.99, 134.04, 129.99, 129.68, 129.36, 126.63, 125.13, 114.13, 111.31, 108.93, 108.75, 107.58, 105.43, 101.52, 56.29, 44.09, 25.61. HRESIMS *m/z* 379.1216 [M + H]^+^ (calcd for C_21_H_18_N_2_O_5_, 319.1246).

##### (E)-3-(4-fluorobenzylidene)-6,7-dimethoxy-2,3-dihydropyrrolo[2,1-b]quinazolin-9(1H)-one (14)

Yield: 73%; yellow solid; ^1^H NMR (400 MHz, CDCl_3_) *δ*: 7.73 (d, *J* = 3.0 Hz, 1H), 7.60 (s, 1H), 7.53 (dd, *J* = 8.7, 5.5 Hz, 2H), 7.20–7.07 (m, 3H), 4.30 (t, *J* = 7.2 Hz, 2H), 4.02 (s, 3H), 4.00 (s, 3H), 3.36–3.16 (m, 2H). ^13 ^C NMR (100 MHz, CDCl_3_) δ: 163.95, 160.49, 154.91, 154.33, 148.80, 145.87, 131.87 (d, *J* = 3.4 Hz), 131.4, 131.38, 128.33, 115.99 (d, *J* = 21.7 Hz), 114.25, 107.64, 105.43, 56.31, 56.29, 44.06, 25.53. Purity 96.4% by HPLC. HRESIMS *m/z* 353.1223 [M + H]^+^ (calcd for C_20_H_17_FN_2_O_3_, 353.1262).

##### (E)-3–(3,4-difluorobenzylidene)-6,7-dimethoxy-2,3-dihydropyrrolo[2,1-b]quinazolin-9(1H)-one (15)

Yield: 76%; yellow solid; ^1^H NMR (400 MHz, CDCl_3_) *δ*: 7.67 (d, *J* = 2.8 Hz, 1H), 7.61 (s, 1H), 7.35 (m, 1H), 7.32–7.18 (m, 2H), 7.14 (s, 1H), 4.31 (t, *J* = 7.1 Hz, 2H), 4.02 (s, 3H), 4.01 (s, 3H), 3.26 (t, *J* = 7.3 Hz, 2H). ^13 ^C NMR (100 MHz, CDCl_3_) *δ*: 160.41, 154.96, 153.92, 152.24–151.25 (m), 149.19 (d, *J* = 12.2 Hz), 148.96, 145.78, 132.86 (d, *J* = 2.6 Hz), 132.73 (d, *J* = 5.4 Hz), 127.23, 126.18 (dd, *J* = 6.3, 3.3 Hz), 117.94 (d, *J* = 9.2 Hz), 117.76 (d, *J* = 8.9 Hz), 114.34, 107.68, 105.43, 56.32, 56.30, 44.05, 25.47. Purity 98.6% by HPLC. HRESIMS *m/z* 371.1129 [M + H]^+^ (calcd for C_20_H_16_F_2_N_2_O_3_, 371.1169).

##### (E)-6,7-dimethoxy-3-(4-(trifluoromethyl)benzylidene)-2,3-dihydropyrrolo[2,1-b]quinazolin-9(1H)-one (16)

Yield: 81%; yellow solid; ^1^H NMR (400 MHz, CDCl_3_) *δ*: 7.80 (t, *J* = 2.8 Hz, 1H), 7.74–7.60 (m, 5H), 7.17 (s, 1H), 4.33 (t, *J* = 7.2 Hz, 2H), 4.03 (s, 3H), 4.02 (s, 3H), 3.32 (t, *J* = 7.2 Hz, 2H). ^13 ^C NMR (100 MHz, CDCl_3_) *δ*: 160.44, 155.01, 153.86, 149.09, 145.78, 138.97, 134.56, 129.61, 127.84, 125.79, 125.75, 114.45, 107.77, 105.47, 56.35, 56.32, 53.40, 44.07, 25.72. Purity 94.4% by HPLC. HRESIMS *m/z* 403.1191 [M + H]^+^ (calcd for C_21_H_17_F_3_N_2_O_3_, 403.1145).

##### (E)-6-(4-hydroxy-3,5-dimethoxybenzylidene)-6,7,8,9-tetrahydro-11H-pyrido[2,1-b]quinazolin-11-one (17)

Yield: 79%; yellow solid; ^1^H NMR (400 MHz, CDCl_3_) *δ*: 8.36–8.14 (m, 1H), 8.08 (d, *J* = 2.1 Hz, 1H), 7.71–7.52 (m, 2H), 7.35 (m, 1H), 6.68 (s, 2H), 5.70 (s, 1H), 4.25–4.03 (m, 2H), 3.86 (s, 6H), 2.91 (t, *J* = 6.6 Hz, 2H), 1.98 (t, *J* = 10.5 Hz, 2H). ^13 ^C NMR (100 MHz, CDCl_3_) *δ*: 161.14, 150.95, 146.60, 145.86, 134.80, 134.46, 133.11, 127.16, 126.64, 126.19, 125.66, 125.03, 119.03, 106.39, 55.40, 41.06, 24.99, 21.12. Purity 97.0% by HPLC. HRESIMS *m/z* 365.1423 [M + H]^+^ (calcd for C_21_H_20_N_2_O_4_, 365.1433).

##### (E)-6-(3,4,5-trimethoxybenzylidene)-6,7,8,9-tetrahydro-11H-pyrido[2,1-b]quinazolin-11-one (18)

Yield: 83%; yellow solid; ^1^H NMR (400 MHz, CDCl_3_) *δ*: 8.26–8.15 (m, 1H), 8.10 (d, *J* = 2.1 Hz, 1H), 7.66 (dd, *J* = 6.1, 1.6 Hz, 2H), 7.36 (dd, *J* = 8.2, 6.0 Hz, 1H), 6.64 (s, 2H), 4.19–4.02 (m, 2H), 3.83 (s, 3H), 3.82 (s, 6H), 2.91 (m, 2H), 1.98 (m, 2H). ^13 ^C NMR (100 MHz, CDCl_3_) *δ*: 161.15, 152.02, 150.68, 146.53, 137.43, 134.48, 133.14, 130.76, 128.41, 126.26, 125.66, 125.16, 119.11, 106.49, 59.96, 55.23, 41.19, 24.89, 21.10. Purity 83.1% by HPLC. HRESIMS *m/z* 379.1580 [M + H]^+^ (calcd for C_22_H_22_N_2_O_4_, 379.1610).

##### (E)-6-(benzo[d][1,3]dioxol-5-ylmethylene)-6,7,8,9-tetrahydro-11H-pyrido[2,1-b]quinazolin-11-one (19)

Yield: 80%; yellow solid; ^1^H NMR (400 MHz, CDCl_3_) *δ*: 8.27 (d, *J* = 8.0 Hz, 1H), 8.12 (d, *J* = 2.2 Hz, 1H), 7.72 (dd, *J* = 6.7, 1.7 Hz, 2H), 7.51–7.38 (m, 1H), 7.00 (d, *J* = 7.1 Hz, 2H), 6.87 (d, *J* = 8.3 Hz, 1H), 6.02 (s, 2H), 4.25–4.00 (m, 2H), 2.94 (m, 2H), 2.04 (m, 2H). ^13 ^C NMR (100 MHz, CDCl_3_) *δ*: 162.14, 151.98, 147.74, 147.70, 147.61, 135.26, 134.11, 130.42, 128.48, 127.25, 126.66, 126.05, 125.09, 120.08, 109.82, 108.41, 101.35, 42.07, 25.94, 22.11. Purity 91.3% by HPLC. HRESIMS *m/z* 333.1161 [M + H]^+^ (calcd for C_20_H_16_N_2_O_3_, 333.1252).

##### (E)-6-(4-fluorobenzylidene)-6,7,8,9-tetrahydro-11H-pyrido[2,1-b]quinazolin-11-one (20)

Yield: 85%; yellow solid; ^1^H NMR (400 MHz, CDCl_3_) *δ*: 8.40 8.23 (m, 1H), 8.18 (d, *J* = 2.2 Hz, 1H), 7.73 (dd, *J* = 7.0, 1.7 Hz, 2H), 7.45 (td, *J* = 8.5, 5.7 Hz, 3H), 7.12 (t, *J* = 8.7 Hz, 2H), 4.32–3.93 (m, 2H), 3.03–2.71 (m, 2H), 2.12–1.90 (m, 2H). ^13 ^C NMR (100 MHz, CDCl_3_) *δ*: 163.65, 162.12, 151.63, 147.51, 134.19 (d, *J* = 4.6 Hz), 132.37 (d, *J* = 3.4 Hz), 131.80 (d, *J* = 8.1 Hz), 129.84 (d, *J* = 1.7 Hz), 127.30, 126.68, 126.24, 120.17, 115.61, 115.40, 42.19, 25.71, 22.06. Purity 98.5% by HPLC. HRESIMS *m/z* 307.1168 [M + H]^+^ (calcd for C_19_H_15_FN_2_O, 307.1299).

##### (E)-2-fluoro-6-(4-hydroxy-3,5-dimethoxybenzylidene)-6,7,8,9-tetrahydro-11H-pyrido[2,1-b]quinazolin-11-one (21)

Yield: 76%; yellow solid; ^1^H NMR (400 MHz, CDCl_3_) *δ*: 8.12 (s, 1H), 7.99–7.83 (m, 1H), 7.71 (dd, *J* = 9.0, 4.8 Hz, 1H), 7.52–7.41 (m, 1H), 6.74 (s, 2H), 5.74 (s, 1H), 4.17 (t, *J* = 5.9 Hz, 2H), 3.93 (s, 6H), 2.99 (t, *J* = 6.6 Hz, 2H), 2.12–2.01 (m, 2H). ^13 ^C NMR (100 MHz, CDCl_3_) *δ*: 161.60 (d, *J* = 18.5 Hz), 151.33, 146.89, 144.36, 135.80, 135.53, 129.61 (d, *J* = 8.1 Hz), 127.94, 127.57, 123.04, 122.80, 121.06 (d, *J* = 8.8 Hz), 111.36 (d, *J* = 23.4 Hz), 107.40, 56.43, 42.25, 25.95, 22.08. Purity 98.2% by HPLC. HRESIMS *m/z* 383.1329 [M + H]^+^ (calcd for C_21_H_19_FN_2_O_4_, 383.1324).

##### (E)-2-fluoro-6-(3,4,5-trimethoxybenzylidene)-6,7,8,9-tetrahydro-11H-pyrido[2,1-b]quinazolin-11-one (22)

Yield: 82%; yellow solid; ^1^H NMR (400 MHz, CDCl_3_) *δ*: 8.14 (d, *J* = 2.2 Hz, 1H), 7.90 (dd, *J* = 8.5, 3.0 Hz, 1H), 7.72 (dd, *J* = 9.0, 4.9 Hz, 1H), 7.46 (d, *J* = 3.0 Hz, 1H), 6.71 (s, 2H), 4.29–4.07 (m, 2H), 3.91 (s, 3H), 3.90 (s, 6H), 2.98 (td, *J* = 6.6, 2.1 Hz, 2H), 2.05 (t, *J* = 6.2 Hz, 2H). ^13 ^C NMR (100 MHz, CDCl_3_) *δ*: 161.76, 161.49, 159.29, 153.06, 151.05 (d, *J* = 2.3 Hz), 144.28, 138.52, 135.48, 131.67, 129.67 (d, *J* = 8.0 Hz), 129.17, 122.93 (d, *J* = 24.3 Hz), 121.15 (d, *J* = 8.6 Hz), 111.36 (d, *J* = 23.5 Hz), 107.51, 60.98, 56.26, 42.38, 25.85, 22.04. Purity 77.8% by HPLC. HRESIMS *m/z* 397.1485 [M + H]^+^ (calcd for C_22_H_21_FN_2_O_4_, 397.1504).

##### (E)-6-(benzo[d][1,3]dioxol-5-methylene)-2-fluoro-6,7,8,9-tetrahydro-11H-pyrido[2,1-b]quinazolin-11-one (23)

Yield: 84%; yellow solid; ^1^H NMR (400 MHz, CDCl_3_) *δ*: 8.11 (s, 1H), 7.89 (dd, *J* = 8.6, 3.1 Hz, 1H), 7.75 (d, *J* = 7.9 Hz, 1H), 7.54–7.42 (m, 1H), 7.01 (d, *J* = 8.9 Hz, 2H), 6.88 (d, *J* = 8.0 Hz, 1H), 6.02 (s, 2H), 4.16 (t, *J* = 5.9 Hz, 2H), 3.07–2.84 (m, 2H), 2.11–1.91 (m, 2H). ^13 ^C NMR (100 MHz, CDCl_3_) *δ*: 161.73, 161.43, 159.26, 151.45, 147.82, 147.78, 135.54, 130.27, 129.57, 129.47, 125.20, 122.96 (d, *J* = 24.3 Hz), 121.04 (d, *J* = 8.7 Hz), 111.38 (d, *J* = 23.5 Hz), 109.81, 108.45, 101.39, 42.24, 25.88, 22.02. HRESIMS *m/z* 351.1067 [M + H]^+^ (calcd for C_20_H_15_FN_2_O_3_, 351.1123).

##### (E)-2-fluoro-6-(4-fluorobenzylidene)-6,7,8,9-tetrahydro-11H-pyrido[2,1-b]quinazolin-11-one (24)

Yield: 75%; yellow solid; ^1^H NMR (400 MHz, CDCl_3_) *δ*: 8.16 (s, 1H), 7.90 (dd, *J* = 8.5, 3.0 Hz, 1H), 7.72 (dd, *J* = 9.0, 4.9 Hz, 1H), 7.51–7.41 (m, 3H), 7.12 (t, *J* = 8.6 Hz, 2H), 4.20–4.12 (m, 2H), 2.97–2.88 (m, 2H), 2.11–1.91 (m, 2H). ^13 ^C NMR (100 MHz, CDCl_3_) *δ*: 163.70, 161.80, 161.43 (d, *J* = 3.6 Hz), 161.22, 159.34, 151.01 (d, *J* = 2.4 Hz), 144.15, 134.30, 132.26 (d, *J* = 3.5 Hz), 131.80 (d, *J* = 8.2 Hz), 129.66 (d, *J* = 8.1 Hz), 129.49, 122.96 (d, *J* = 24.4 Hz), 121.19 (d, *J* = 8.7 Hz), 115.54 (d, *J* = 21.5 Hz), 111.38 (d, *J* = 23.5 Hz), 42.37, 25.65, 21.98. Purity 95.0% by HPLC. HRESIMS *m/z* 325.1074 [M + H]^+^ (calcd for C_19_H_14_F_2_N_2_O, 325.1153).

##### (E)-3–(3,4-difluorobenzylidene)-7-fluoro-6-(4-methylpiperazin-1-yl)-2,3-dihydropyrrolo[2,1-b]quinazolin-9(1H)-one (25)

Yield: 69%; yellow solid; ^1^H NMR (400 MHz, CDCl_3_) *δ*: 7.76 (d, *J* = 12.9 Hz, 1H), 7.61 (d, *J* = 3.1 Hz, 1H), 7.32–7.24 (m, 1H), 7.23–7.12 (m, 2H), 7.08 (d, *J* = 7.8 Hz, 1H), 4.20 (t, *J* = 7.1 Hz, 2H), 3.24 (t, *J* = 4.8 Hz, 4H), 3.17 (d, *J* = 9.8 Hz, 2H), 2.56 (t, *J* = 4.9 Hz, 4H), 2.31 (s, 3H). ^13 ^C NMR (100 MHz, CDCl_3_) *δ*: 159.11, 154.14 (d, *J* = 44.5 Hz), 151.88, 150.65 (d, *J* = 12.7 Hz), 148.16 (d, *J* = 16.1 Hz), 146.51, 145.35 (d, *J* = 10.0 Hz), 131.67, 126.89, 125.27, 117.06, 116.89, 116.73, 114.17 (d, *J* = 3.6 Hz), 113.51 (d, *J* = 8.4 Hz), 111.04 (d, *J* = 23.7 Hz), 53.89, 48.91 (d, *J* = 4.7 Hz), 45.09, 42.93, 24.38. HRESIMS *m/z* 427.1667 Purity 99.3% by HPLC. [M + H]^+^ (calcd for C_23_H_21_F_3_N_4_O, 427.1769).

##### (E)-7-fluoro-6-(4-methylpiperazin-1-yl)-3–(4-(trifluoromethyl)benzylidene)-2,3-dihydropyrrolo[2,1-b]quinazolin-9(1H)-one (26)

Yield: 78%; white solid; ^1^H NMR (400 MHz, CDCl_3_) *δ*: 7.83–7.69 (m, 2H), 7.65–7.49 (m, 4H), 7.09 (d, *J* = 7.8 Hz, 1H), 4.21 (t, *J* = 7.1 Hz, 2H), 3.23 (m, 6H), 2.56 (t, *J* = 4.8 Hz, 4H), 2.31 (s, 3H). ^13 ^C NMR (100 MHz, CDCl_3_) *δ*: 159.07 (d, J = 3.2 Hz), 154.42, 153.82 (d, *J* = 2.2 Hz), 151.94, 146.49, 145.37 (d, *J* = 10.1 Hz), 137.82, 133.38, 129.40 (d, *J* = 32.8 Hz), 128.66, 127.43, 124.76 (q, *J* = 3.8 Hz), 114.24 (d, *J* = 3.6 Hz), 113.57 (d, *J* = 8.5 Hz), 111.06 (d, *J* = 23.8 Hz), 53.89, 48.91 (d, *J* = 4.7 Hz), 45.08, 42.94, 24.61. Purity 99.7% by HPLC. HRESIMS *m/z* 459.1730 [M + H]^+^ (calcd for C_24_H_22_F_4_N_4_O, 459.1257).

### Cell lines and cell culture

Human cancer cell lines HCT116, AGS, PANC-1 cells were purchased from the ATCC (USA). SMMZ-7721 and normal gastric cell line GES-1 were obtained from School of Pharmacy, Lanzhou University. All cells except PANC-1 and GES-1 were cultured in 1640 medium (1640, Solarbio Invitrogen Corp., Beijing, China). PANC-1 and GES-1 cell lines were cultured in high glucose Dulbecco’s modified eagle medium (Solarbio Invitrogen Corp., Beijing, China). The cells were all supplemented with 10% foetal bovine serum (FBS), 100.0 U/ml penicillin, and 100.0 mg/ml streptomycin at 37 °C with 5% CO_2_.

### MTT assay

Cells were cultured in 96-well plates (1 × 10^4^ cells/100 μl) and pre-incubated at 37 °C for 12 h to make them attached. The cells were incubated with the compounds at different doses for 48 h. After treatment, each well was added with 10.0 μl MTT solution (5.0 mg/ml) and continued to incubate at 37 °C for 4 h. The medium was discarded and 100 μl DMSO were added into each well. The IC_50_ value of the derivatives were then determined by a microplate reader (SpectraMax190, USA) through reading the OD value of each well at wavelength 490 nm.

### Cell cycle analysis

For cell-cycle analysis, AGS cells (7 × 10^5^/well) were seeded in 6-well plates and treated with DMSO or different concentrations of Compound **6**. The cells were then harvested, washed with cold PBS, fixed with 70% ethanol at 4 °C, and washed with PBS three times again. After that the cells were re-suspended in 100 μl RNaseA and incubated at 37 °C for 30 min to be stained with 400 μl propidium iodide. At last, DNA content was analysed by a flow cytometer. BD LSRFortessa (USA) was used to analyse the obtained data.

### Transwell assay

To analyse the invasion activity, a transwell apparatus possessing 8 μm pore membrane was applied. Cells were cultured and suspended in serum-free RPMI-1640 medium at a density of 3 × 10^4^ cells/100 μl. The suspension of the cells was seeded into the upper chamber of the apparatus containing Solarbio Matrigel (8-μm pore size; Corning), followed by 600.0 μl of 20% FBS medium. After culturing for 48 h, the embedded cells were fixed and stained with 4% paraformaldehyde and 0.1% crystal violet for 40 min and 10 min, respectively. The membrane was photographed using an optical microscope.

### Hoechst 33258 staining

AGS cells were seeded on chamber slides in 24-well plates and treated with Compound **6** at different concentrations for 48 h. According to the manufacturer’s instructions (Solarbio life sciences, China), fixed with 4% paraformaldehyde for 30 min at RT, the cells were then washed with PBS for three times and incubated with the 100:1 diluted Hoechst 33258 (10.0 μg/ml) for 10 min at RT. Slides were washed again and examined under a fluorescence microscope (Zeiss, Germany).

### Annexin V-FITC apoptosis assay

AGS cells were seeded on six-well plates and incubated with Compound **6** (2.0, 3.0, 4.0, and 5.0 μM) for 48 h. The cells were then suspended with 100 μl diluted binding buffer adjusting the cell concentration to 1 × 10^6^ cell/ml and 5 μl Annexin-V-fluorescein was added. After incubating the mixture in the dark for 5 min at 25 °C, 10 μl Propidium iodide and 400 μl PBS were added. Measurements were conducted with a flow cytometer (BD LSRFortessa, USA).

### MMP measurement

MMP was determined using the JC-10 kit (Solarbio Invitrogen Corp., Beijing, China). AGS cells were seeded on cover glasses in 24-well plates and incubated for 24 h. The cells were treated with different concentrations of Compound **6** (2.0, 3.0, 4.0, and 5.0 μM) for another 24 h and then were fixed with 4% paraformaldehyde. After washing with PBS for three times, the cells were stained with JC-10 for 20 min at 37 °C. Cells treated with carbonyl cyanide-3-chlorophenylhydrazone (CCCP, 10.0 μM) were used as positive control. The results were obtained by a laser confocal fluorescence microscopy (Zeiss, Germany).

### Docking study

The three-dimensional structures of the PI3Kα (PDB: 6gvf), AKT1 (PDB: 6hhf), mTOR (PDB: 4jt6) and other related proteins were obtained from RCSB PDB database was used for molecular modelling. The Schrodinger 10.2 was employed for docking calculations. Pymol 2.4 was used for graphic display.

### *In vitro* inhibitory activity against AKT1

The *in vitro* AKT1 kinase inhibitory activities of Compound **6** was analysed with Kinase-Glo luminescent kinase assay kits (Promega, USA) in different concentration according to the working manual.

### Western blot

To get cells lysates containing the total proteins, the AGS cells were lysed with RIPA lysis solution. The concentration of the proteins was quantified using the Bradford assay. The samples were applied to 10% SDS-PAGE (sodium dodecyl sulphate-polyacrylamide gel electrophoresis), transferred to a PVDE (poly vinylidene difluoride) membrane. The membrane was then immersed in TBST (Tris-buffered saline and Tween) containing 5% defatted milk for 1 h with gentle agitation and incubated overnight at 4 °C with the primary antibody (GAPDH #60004-Ig purchased from Proteintech; AKT #YT0185; p-AKT #YP0006; mTOR #YT2913 purchased from ImmunoWay; PI3K# YP0176 purchased from Affinity; Bcl-2 #bs-0032R; Bax #bs-0127R; Caspase-3# bs-0081R purchased from Bioss). After that, the membrane was incubated with HRP-labeled (horseradish peroxidase) secondary antibodies for 2 h at room temperature and detected on photographic film. Finally, the bands of the proteins were analysed with ImageJ software.

## Supplementary Material

Supplemental Material

## Supplementary material

Supplementary material includes ^1^H NMR, ^13 ^C NMR, HRESIMS, and HPLC Spectra of the derivatives and molecular docking analysis of Compound **6**.
