# Supplementary material for: Design, synthesis, and cytotoxic activities of isaindigotone derivatives as potential anti-gastric cancer agents
Source: J Enzyme Inhib Med Chem. 2022 Apr 21;37(1):1212–26. doi: 10.1080/14756366.2022.2065672 (PMC9037217; doi:10.1080/14756366.2022.2065672)
Supplement: Supplemental Material [file IENZ_A_2065672_SM9945.pdf]

Supporting Information

Design, synthesis and anti-cancer activity of isaindigotone derivatives as potential antitumor agents

Kangjia Du, Wantong Ma, Chengjie Yang, Zhongkun Zhou, Shujian Hu, Yanan Tian, Hao Zhang, Yunhao Ma, Xinrong Jiang, Hongmei Zhu, Huanxiang Liu, Peng Chen *, Yingqian Liu*

School of Pharmacy, Lanzhou University, 199 Donggang West Road, Lanzhou, 730000, PR China.

^{1}H NMR, ^{13}C NMR, HRESIMS and HPLC Spectra of the Compounds

Molecular docking analysis

---

* Correspondence and requests for materials should be addressed to Peng Chen (email: chenpeng@lzu.edu.cn) or Yingqian Liu (email: yqliu@lzu.edu.cn). Tel. & fax: +86 931 8915686.

1H and 13C NMR spectra of the representative compounds

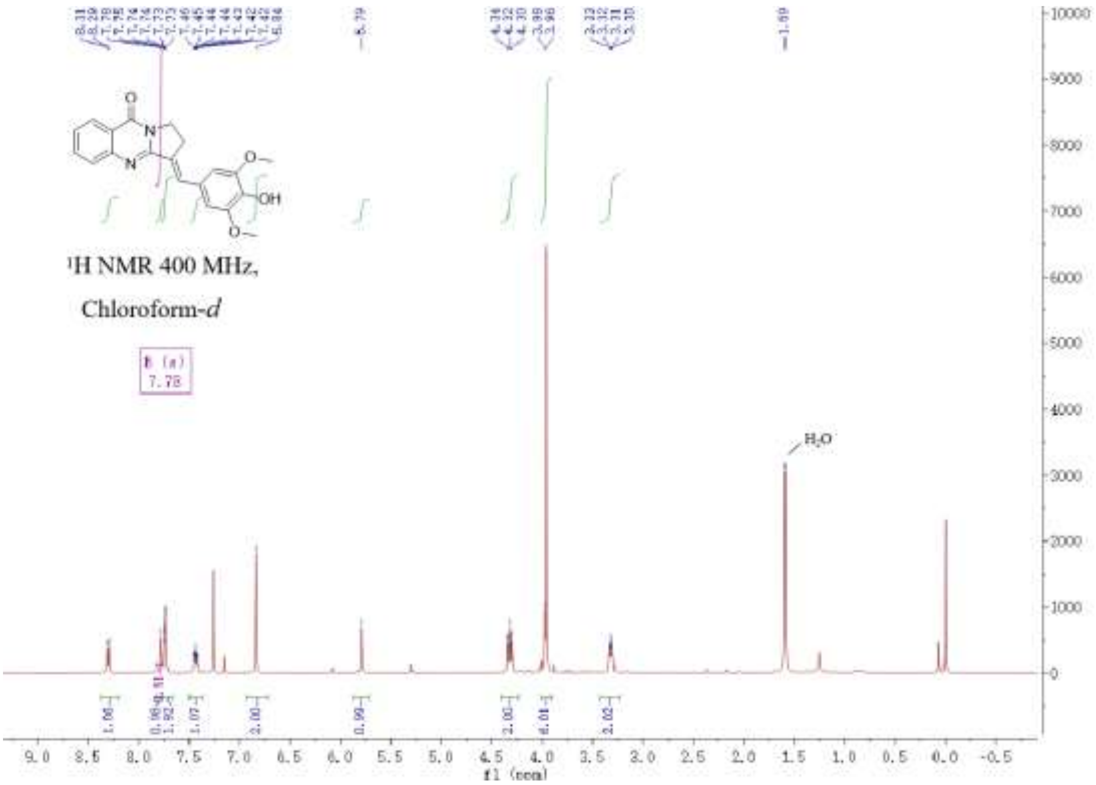

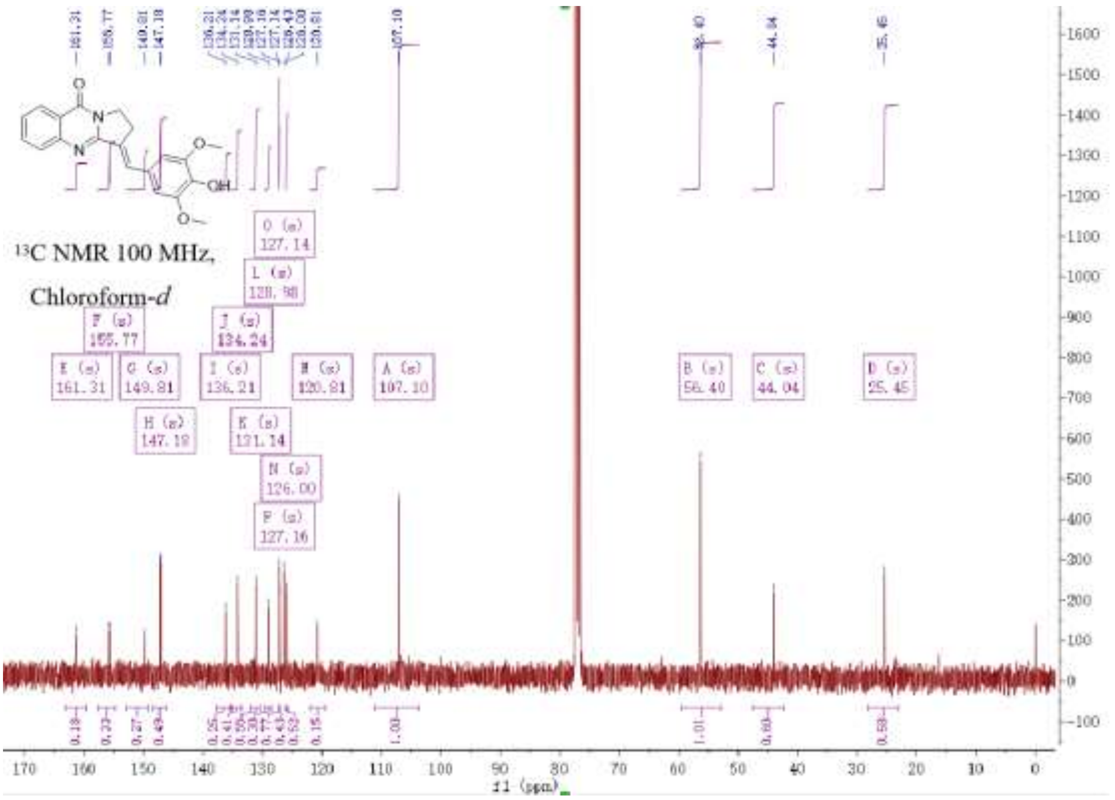

Generic Display Report

Analysis Info

Analysis Name	D:\Data\yangy\new\YANGCHENGJIE210510_1_7_01_39109.d	Acquisition Date	5/10/2021 12:24:36 PM
Method	POS_100-1200_For LC.m	Operator	LZU
Sample Name	YANGCHENGJIE210510_1	Instrument	micrOTOF
Comment			

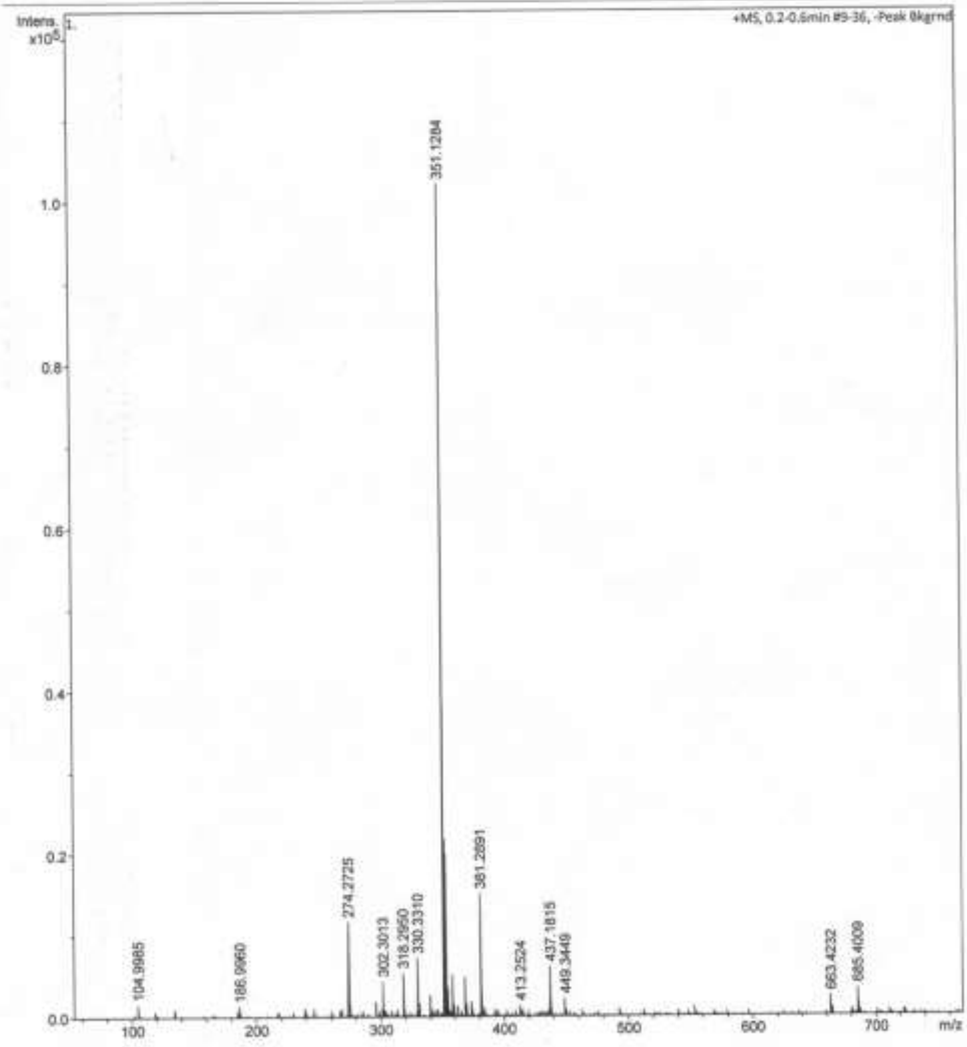

Bruker Compass DataAnalysis 4.1 printed: 5/10/2021 4:58:53 PM by: LZU Page 1 of 1

23

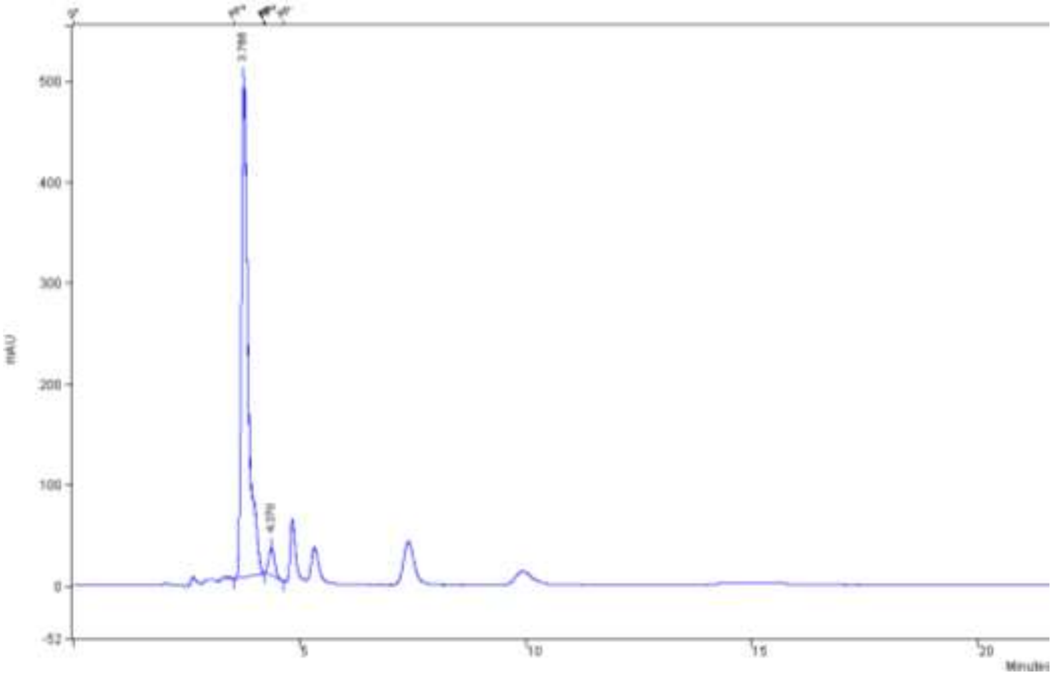

Peak No.	Peak Name	Result ()	Ret. Time (min)	Time Offset (min)	Area (counts)	Sep. Code	Width 1/2 (sec)	Status Codes
1		95.4719	3.768	0.000	51601348	BB	8.5	
2		4.5281	4.370	0.000	2447408	BB	8.1	
	Totals:	100.0000		0.000	54048756			

Figure S1. ^{1}H NMR, ^{13}C NMR, HRESIMS, HPLC spectra of compound **1**.

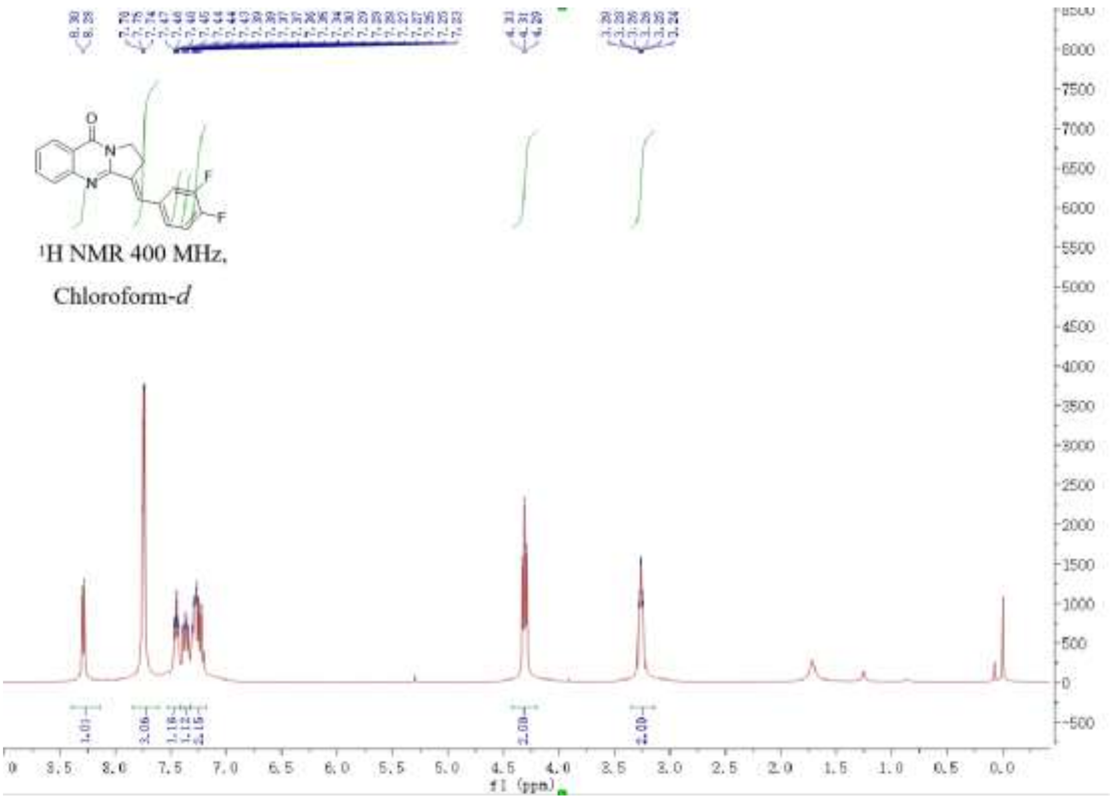

1H NMR 400 MHz,
Chloroform-d
f1 (ppm)

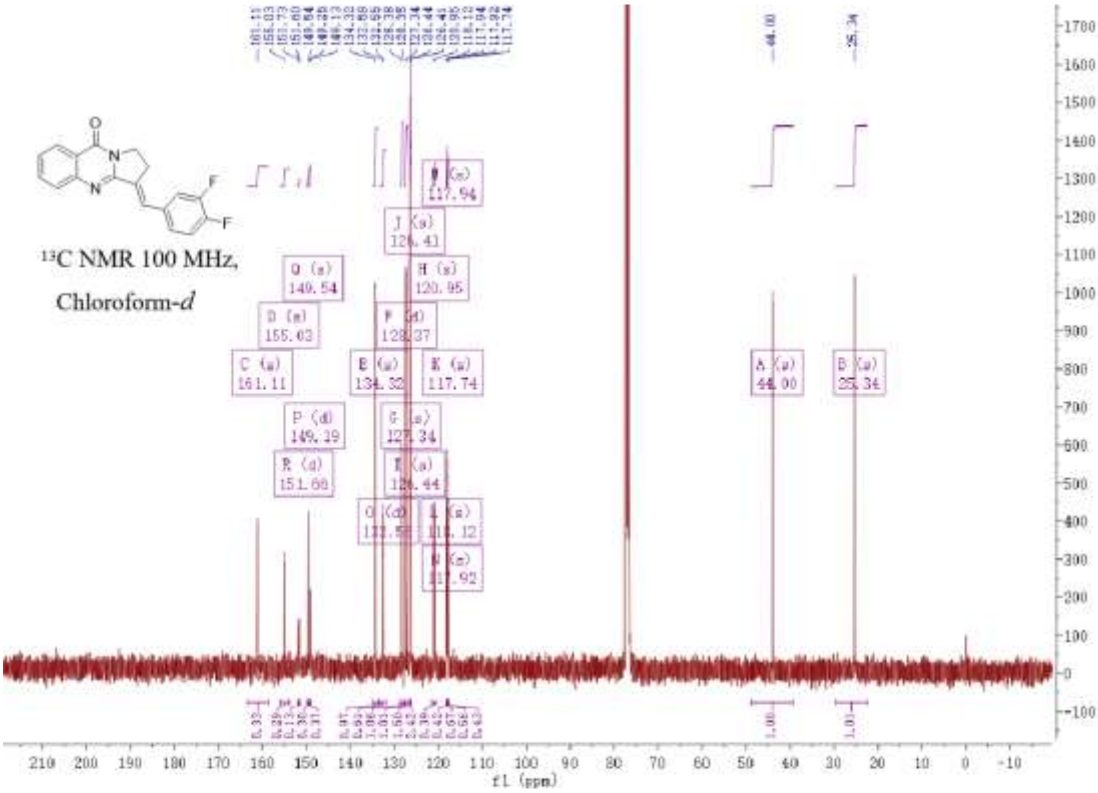

13C NMR 100 MHz,
Chloroform-d
f1 (ppm)

Generic Display Report

**Analysis Info**		Acquisition Date	5/10/2021 12:27:39 PM
Analysis Name	D:\Data\yangy\new\YANGCHENGJIE210510_2_8_01_39110.d		
Method	POS_100-1200_For LC.m	Operator	LZU
Sample Name	YANGCHENGJIE210510_2	Instrument	micrOTOF
Comment			

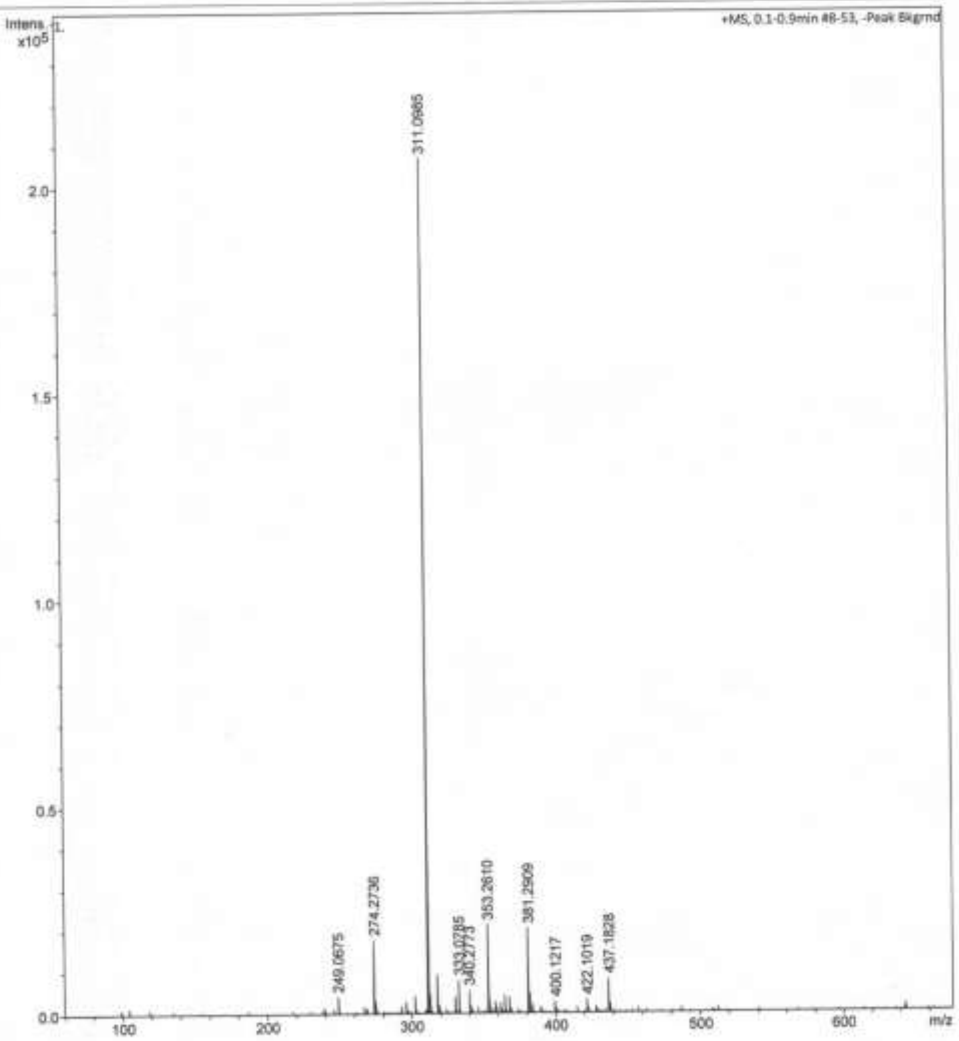

Bruker Compass DataAnalysis 4.1 printed: 5/10/2021 4:59:05 PM by: LZU Page 1 of 1

32

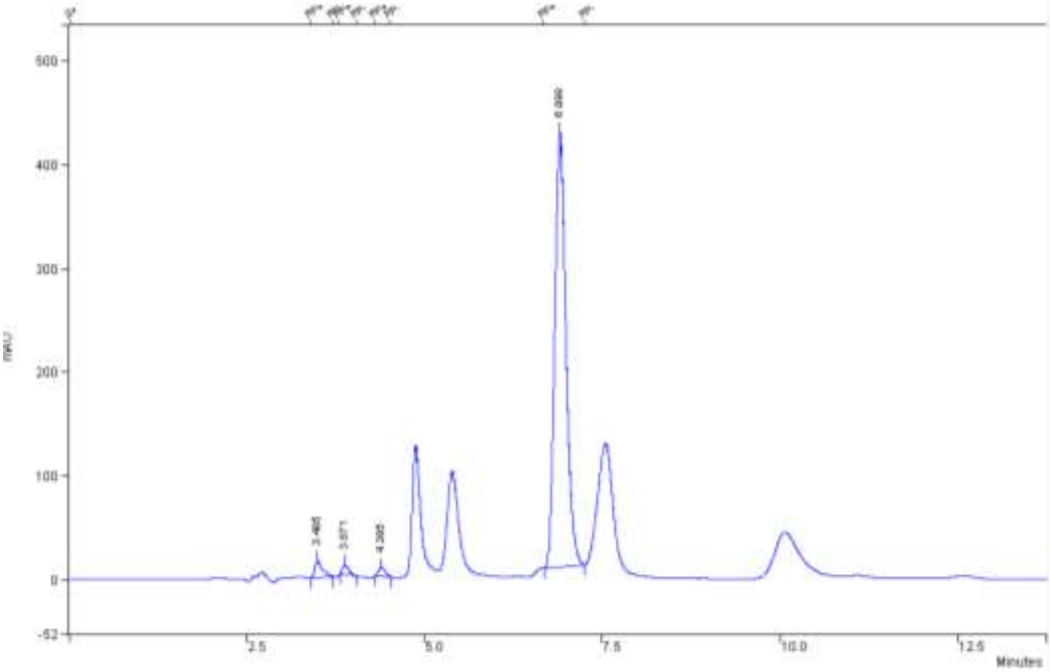

Peak No.	Peak Name	Result ()	Ret. Time (min)	Time Offset (min)	Area (counts)	Sep. Code	Width 1/2 (sec)	Status Codes
1		2.4466	3.485	0.000	1172639	BB	8.9	
2		1.2281	3.871	0.000	588590	BD	6.0	
3		1.1594	4.385	0.000	555669	BB	6.6	
4		95.1659	6.896	0.000	45611736	BD	10.0	
	Totals:	100.0000		0.000	47928634			

Figure S2. ^{1}H NMR, ^{13}C NMR, HRESIMS, HPLC spectra of compound **2**.

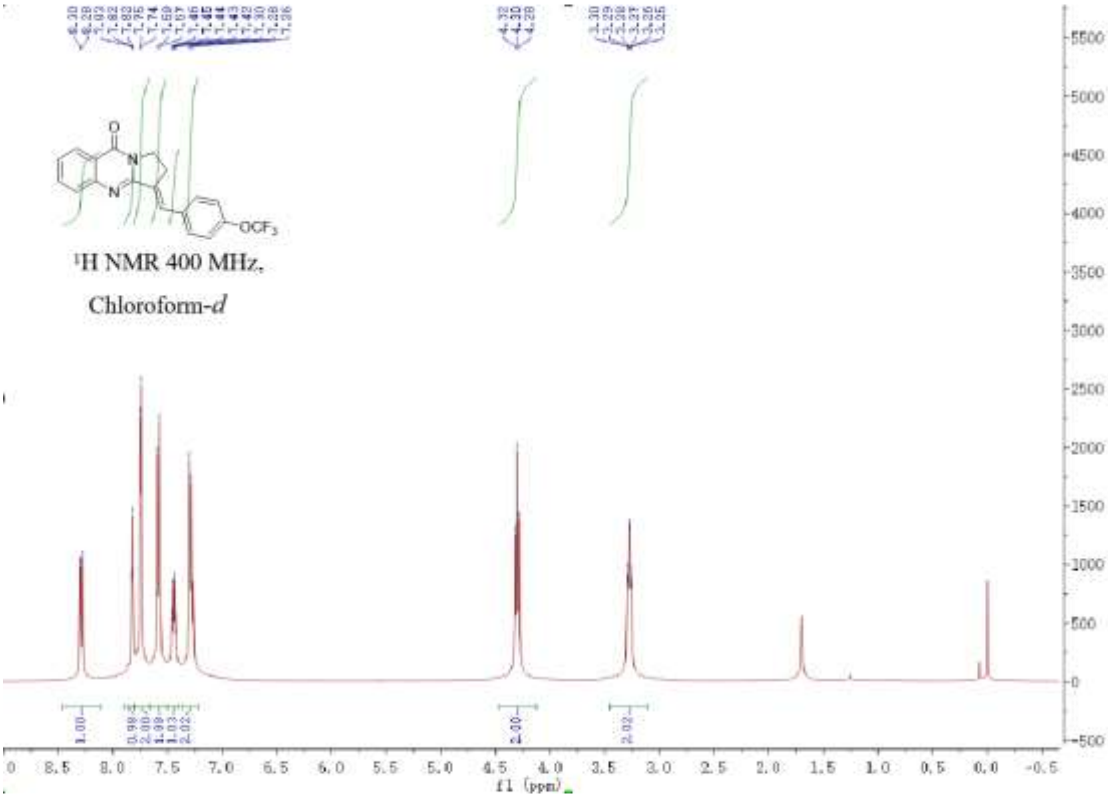

38

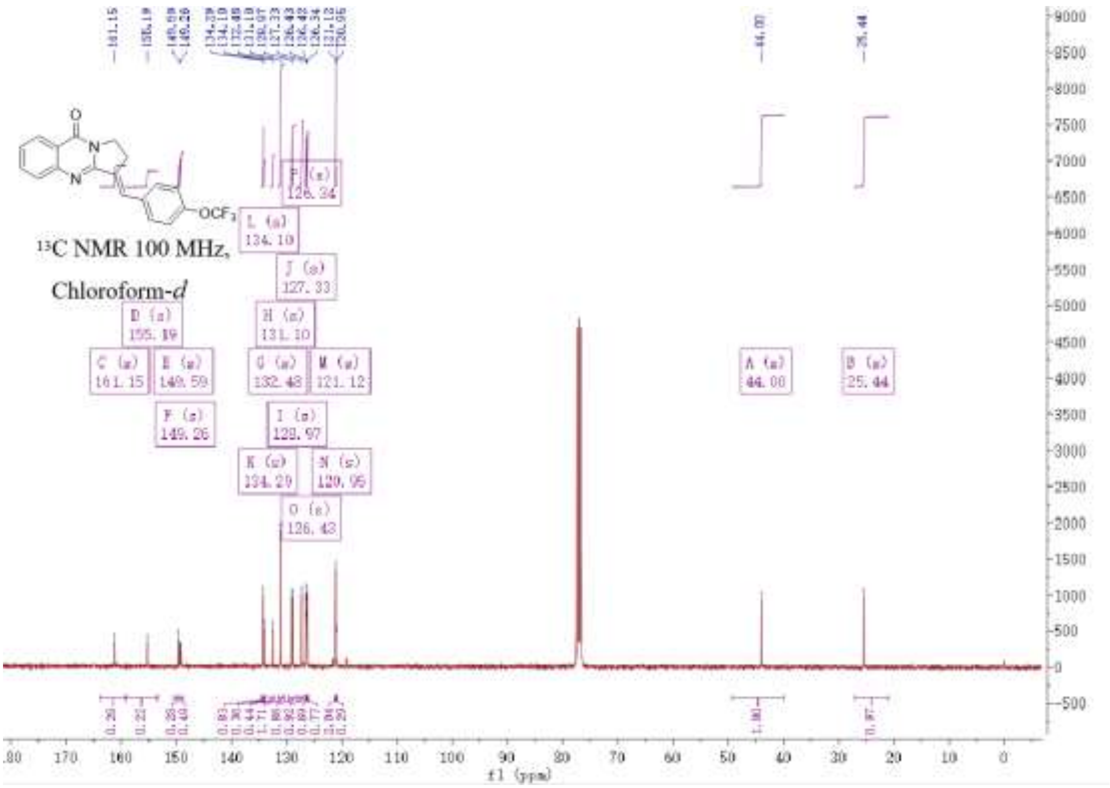

161.15
155.19
149.59
149.26
134.29
134.10
132.48
131.10
128.97
127.33
126.43
126.42
126.34
121.12
120.95
44.00
25.44
OCF3
13C NMR 100 MHz,
Chloroform-d
f1 (ppm)

Generic Display Report

Analysis Info			
Analysis Name	D:\Data\yangy\new\YANGCHENGJIE210510_3_9_01_39111.d	Acquisition Date	5/10/2021 12:30:45 PM
Method	POS_100-1200_For LC.m	Operator	LZU
Sample Name	YANGCHENGJIE210510_3	Instrument	micrOTOF
Comment			

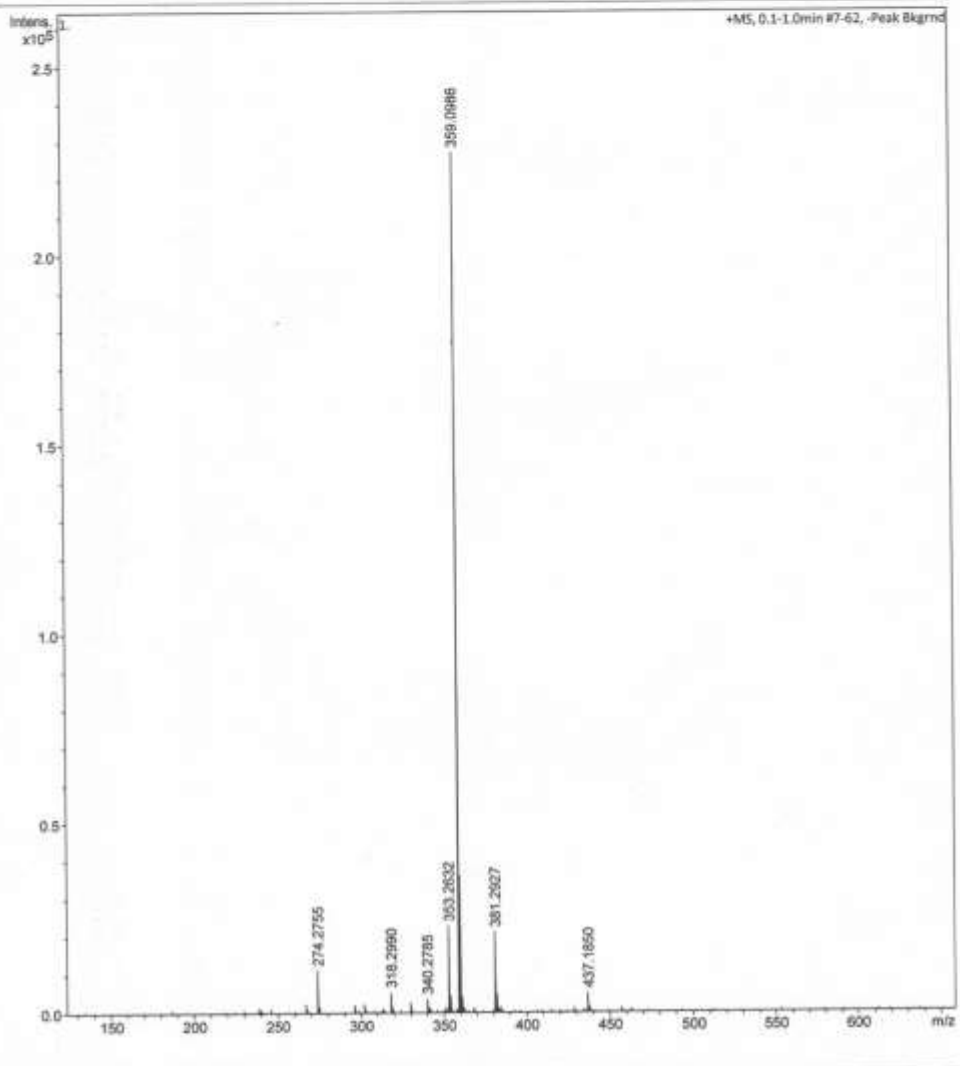

Bruker Compass DataAnalysis 4.1 printed: 5/10/2021 4:59:19 PM by: LZU Page 1 of 1

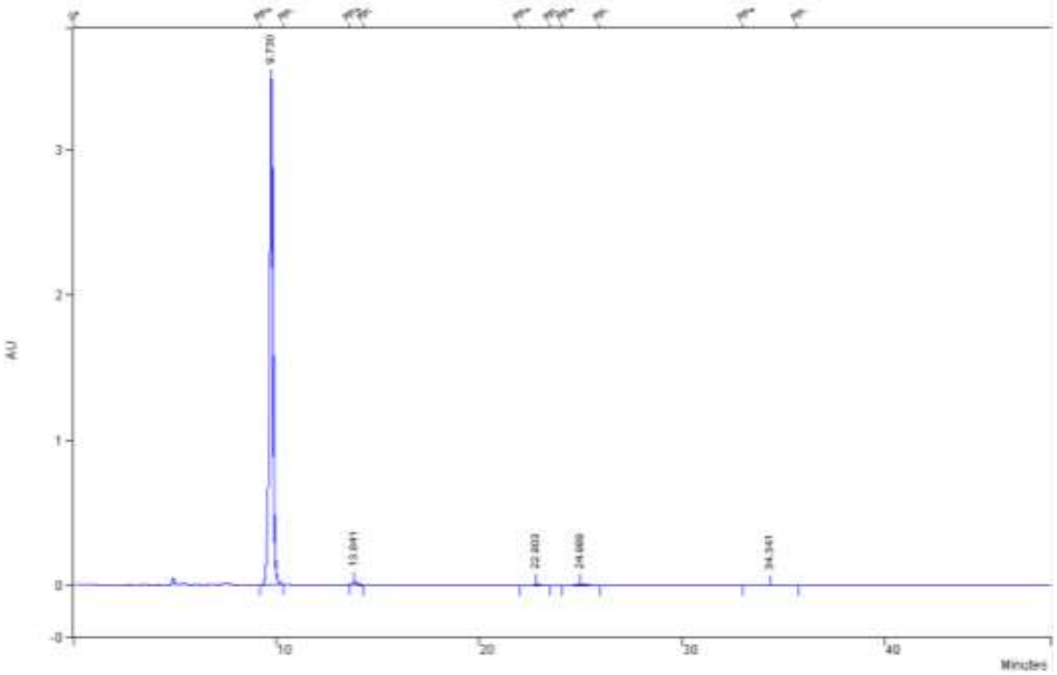

Peak No.	Peak Name	Result ()	Ret. Time (min)	Time Offset (min)	Area (counts)	Sep. Code	Width 1/2 (sec)	Status Codes
1		98.1527	9.730	0.000	397553184	BB	8.8	
2		0.9826	13.841	0.000	3978666	BB	16.6	
3		0.2160	22.803	0.000	875043	BB	31.7	
4		0.5328	24.988	0.000	2157653	BB	33.6	
5		0.1159	34.341	0.000	469528	BB	49.4	
	Totals:	100.0000		0.000	405035474			

Figure S3. ^{1}H NMR, ^{13}C NMR, HRESIMS, HPLC spectra of compound **3**.

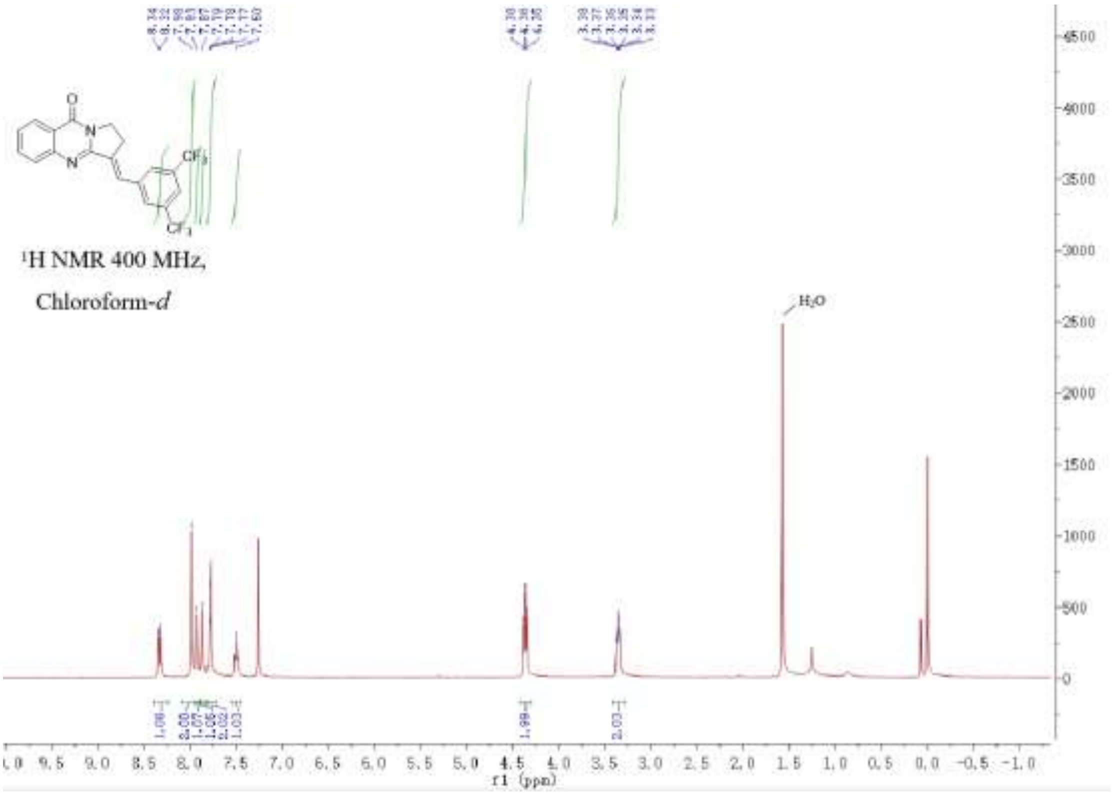

45

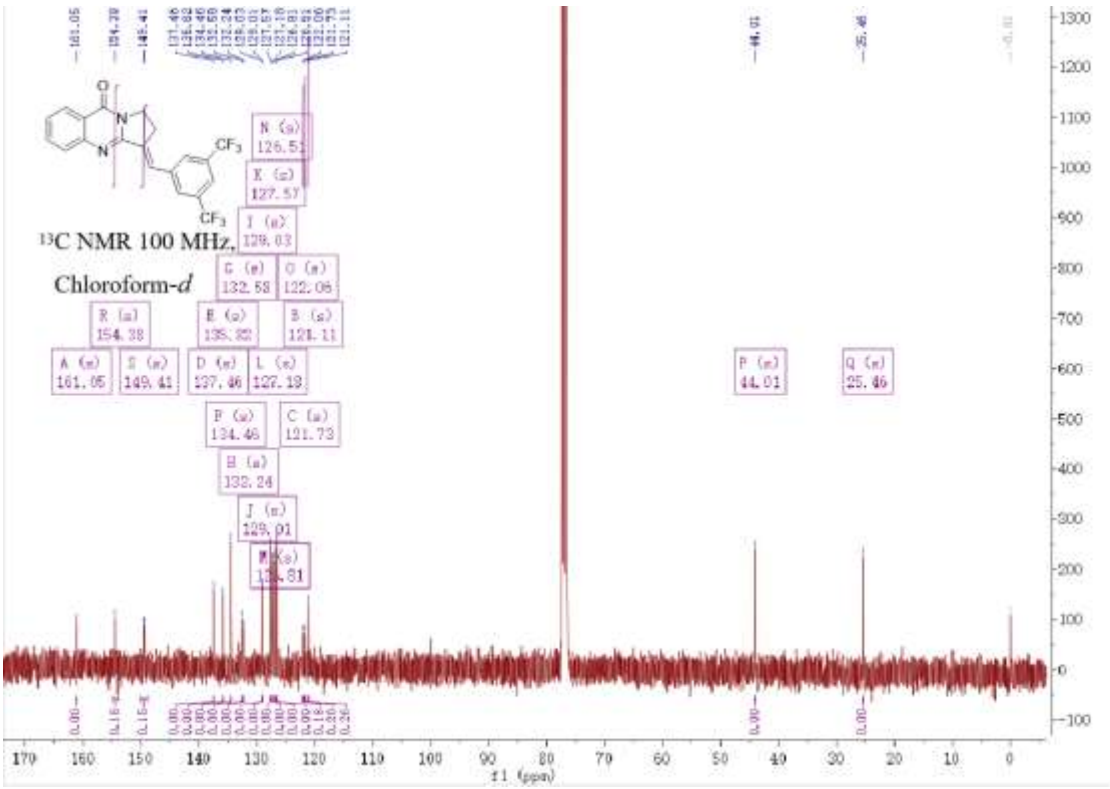

^{13}C NMR 100 MHz,
Chloroform-*d*
A (s) 161.05
R (s) 154.38
S (s) 149.41
D (s) 137.46
E (s) 135.82
F (s) 134.46
G (s) 132.58
H (s) 132.24
I (s) 129.03
J (s) 129.01
K (s) 127.57
L (s) 127.18
N (s) 126.51
O (s) 122.06
B (s) 121.11
C (s) 121.73
P (s) 44.01
Q (s) 25.46
f1 (ppm)

Generic Display Report

**Analysis Info**		Acquisition Date	5/10/2021 12:33:50 PM
Analysis Name	D:\Data\yangy\new\YANGCHENGJIE210510_4_10_01_39112.d		
Method	POS_100-1200_For LC.m	Operator	LZU
Sample Name	YANGCHENGJIE210510_4	Instrument	micrOTOF
Comment			

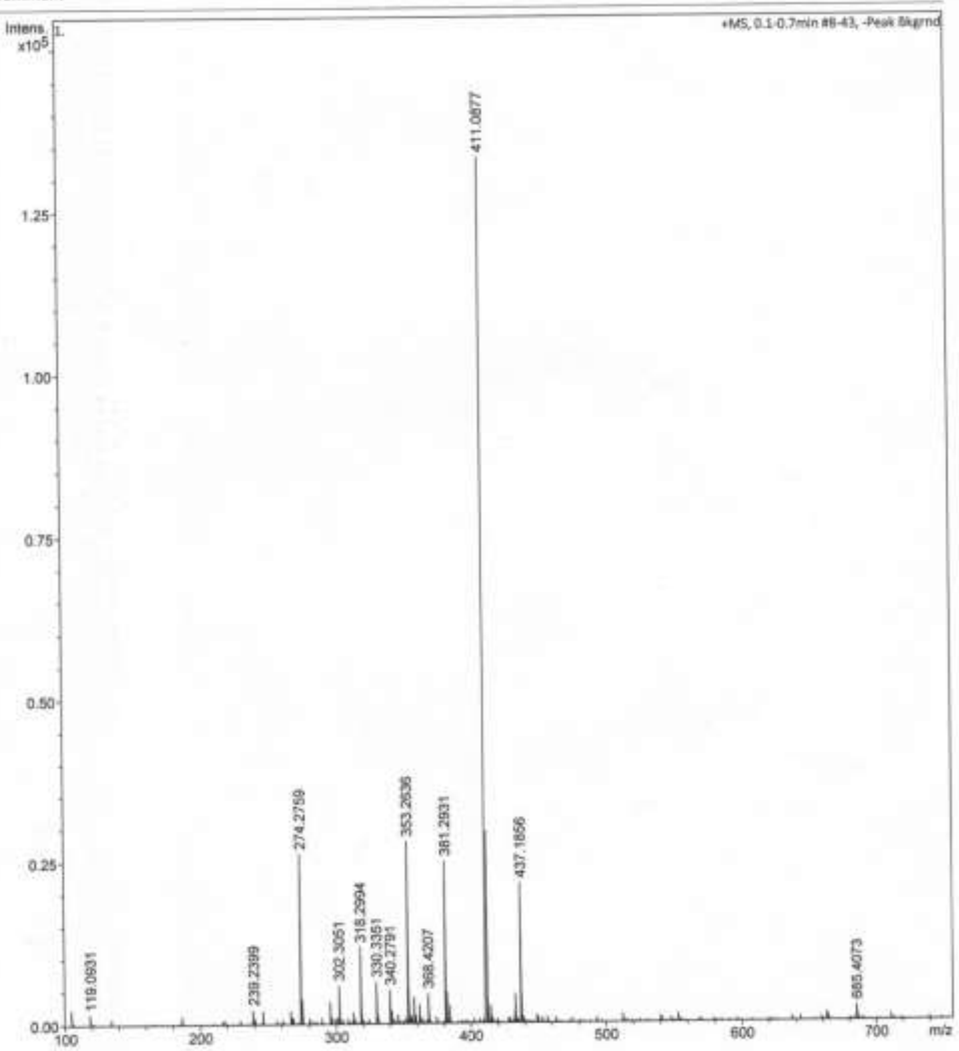

Bruker Compass DataAnalysis 4.1 printed: 5/10/2021 4:59:32 PM by: LZU Page 1 of 1

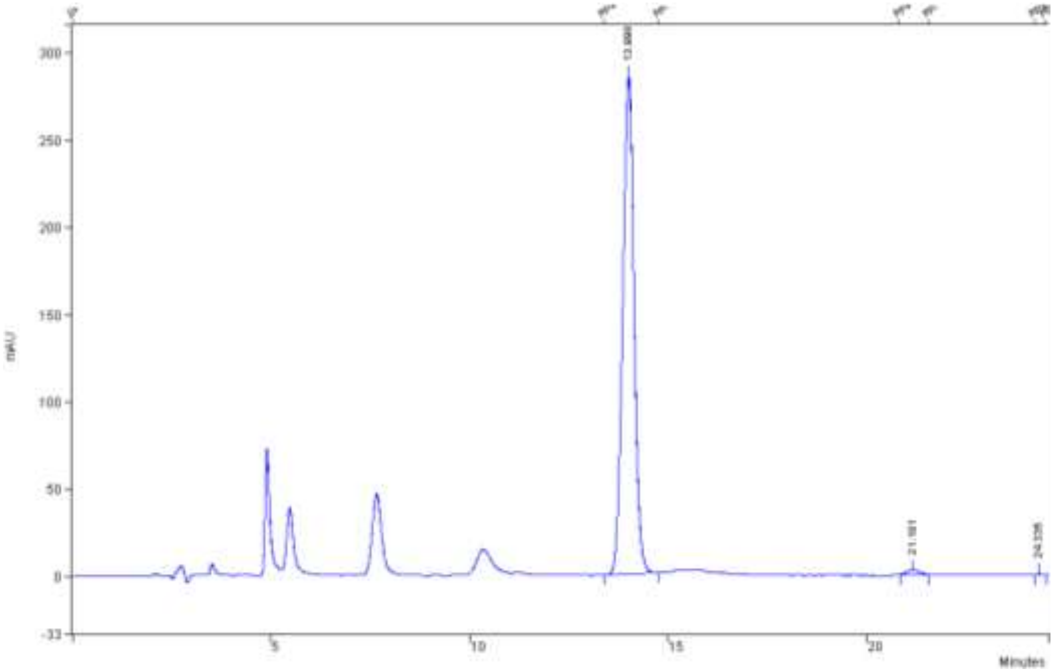

Peak No.	Peak Name	Result ()	Ret. Time (min)	Time Offset (min)	Area (counts)	Sep. Code	Width 1/2 (sec)	Status Codes
1		98.9936	13.998	0.000	56521560	BB	18.2	
2		0.9915	21.181	0.000	566097	BB	21.7	
3		0.0150	24.338	0.000	8548	BB	2.3	
	Totals:	100.0001		0.000	57096206			

Figure S4. ^{1}H NMR, ^{13}C NMR, HRESIMS, HPLC spectra of compound **4**.

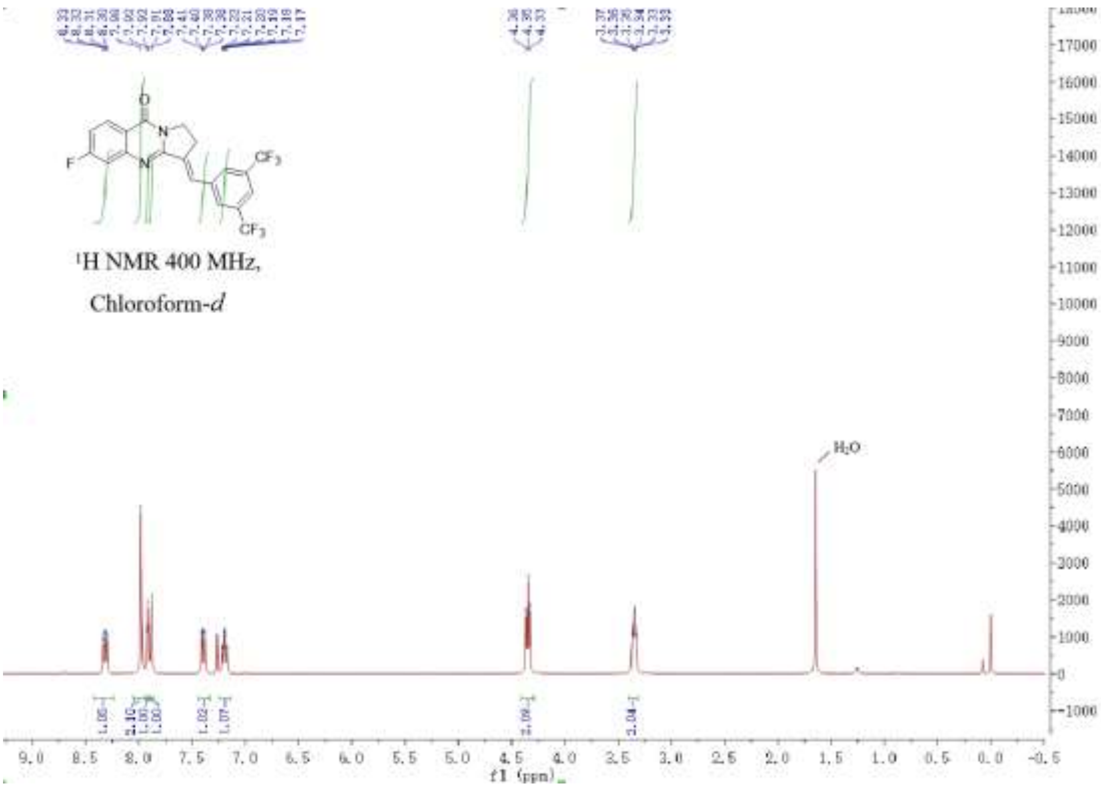

53

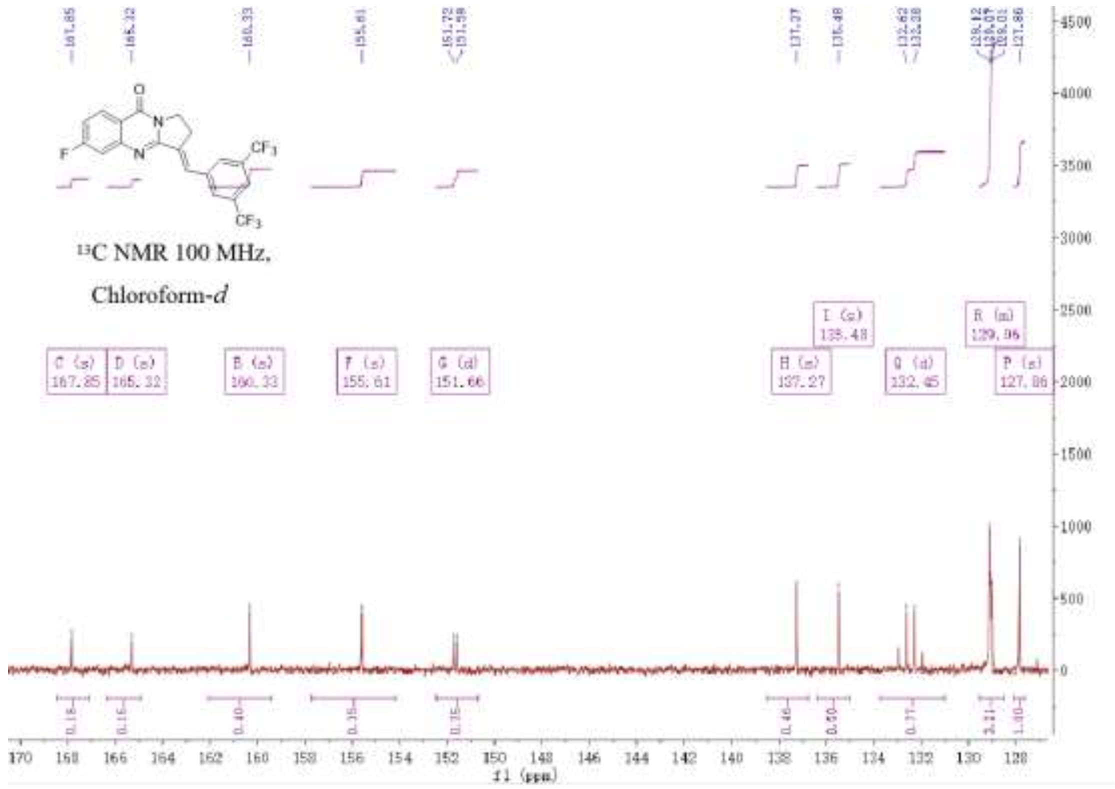

Generic Display Report

Analysis Info

Analysis Name	D:\Data\yangy\new\YANGCHENGJIE210511_2_2_01_39143.d	Acquisition Date	5/11/2021 11:56:10 AM
Method	POS_100-1200_For LC.m	Operator	LZU
Sample Name	YANGCHENGJIE210511_2	Instrument	micrOTOF
Comment			

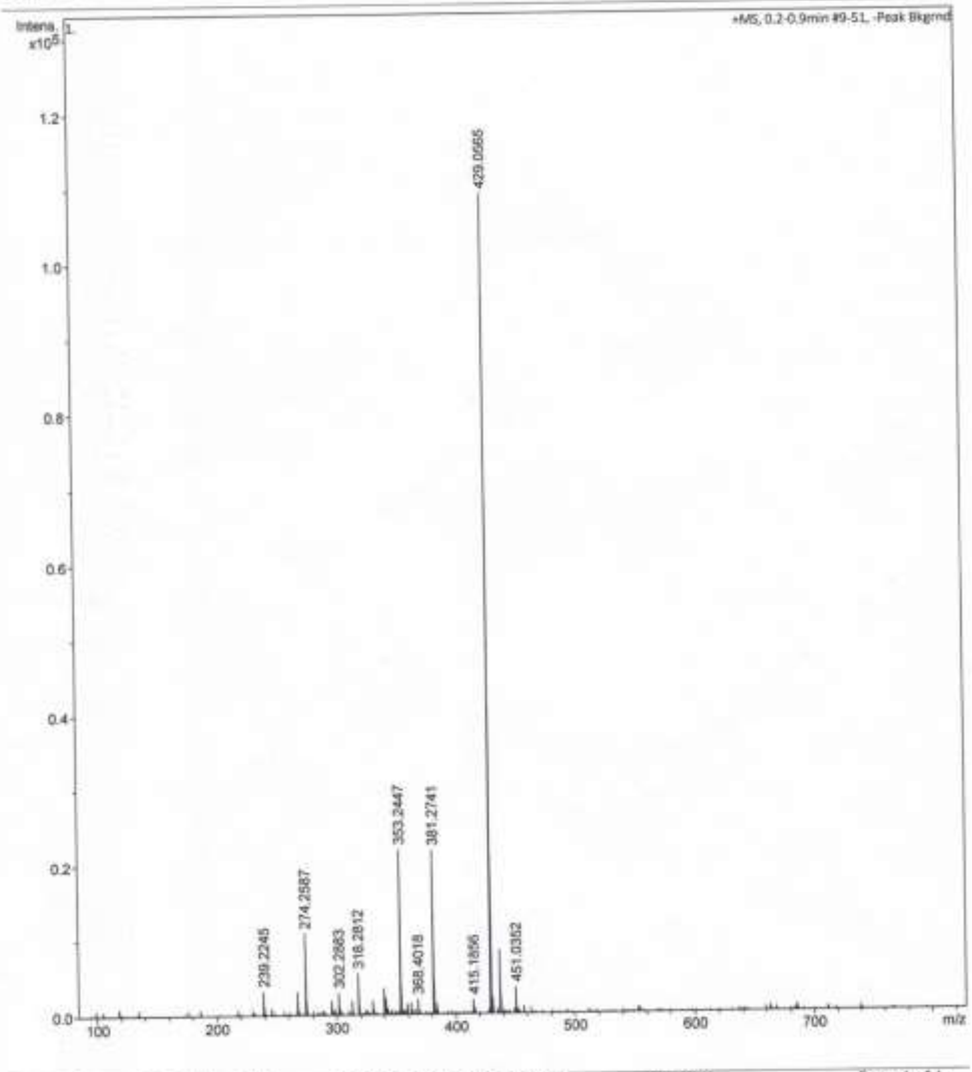

Bruker Compass DataAnalysis 4.1 printed: 5/11/2021 3:17:25 PM by: LZU Page 1 of 1

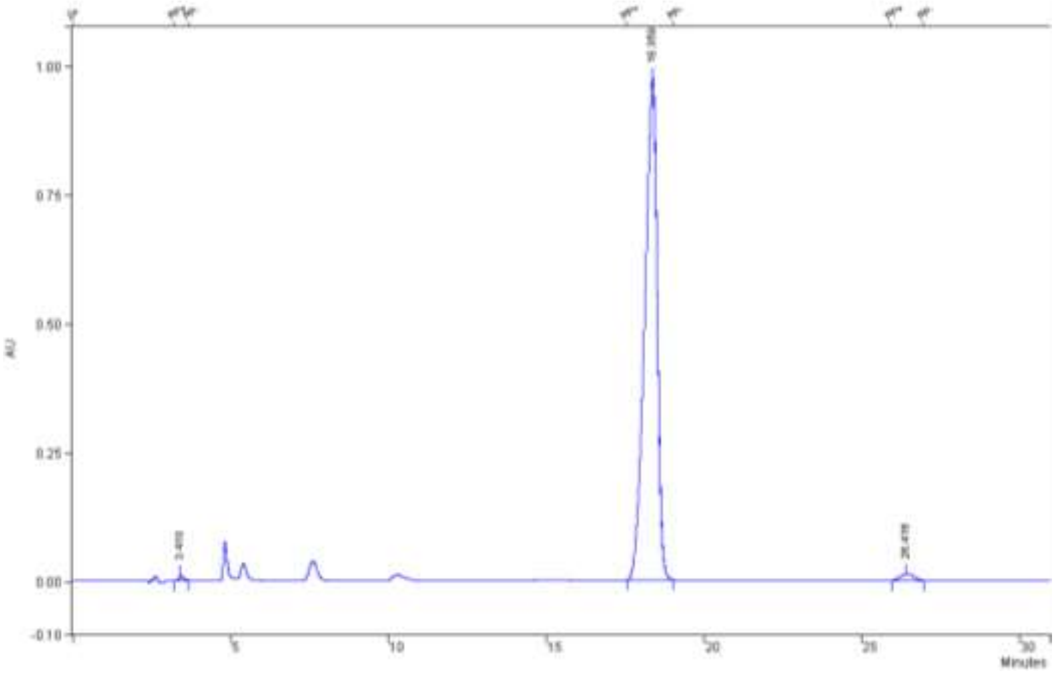

Peak No.	Peak Name	Result ()	Ret. Time (min)	Time Offset (min)	Area (counts)	Sep. Code	Width 1/2 (sec)	Status Codes
1		0.3750	3.410	0.000	1023106	BB	9.1	
2		98.1954	18.359	0.000	267914812	BB	25.6	
3		1.4297	26.416	0.000	3900652	BB	29.0	
	Totals:	100.0001		0.000	272838270			

Figure S7. ^{1}H NMR, ^{13}C NMR, HRESIMS, HPLC spectra of compound **7**.

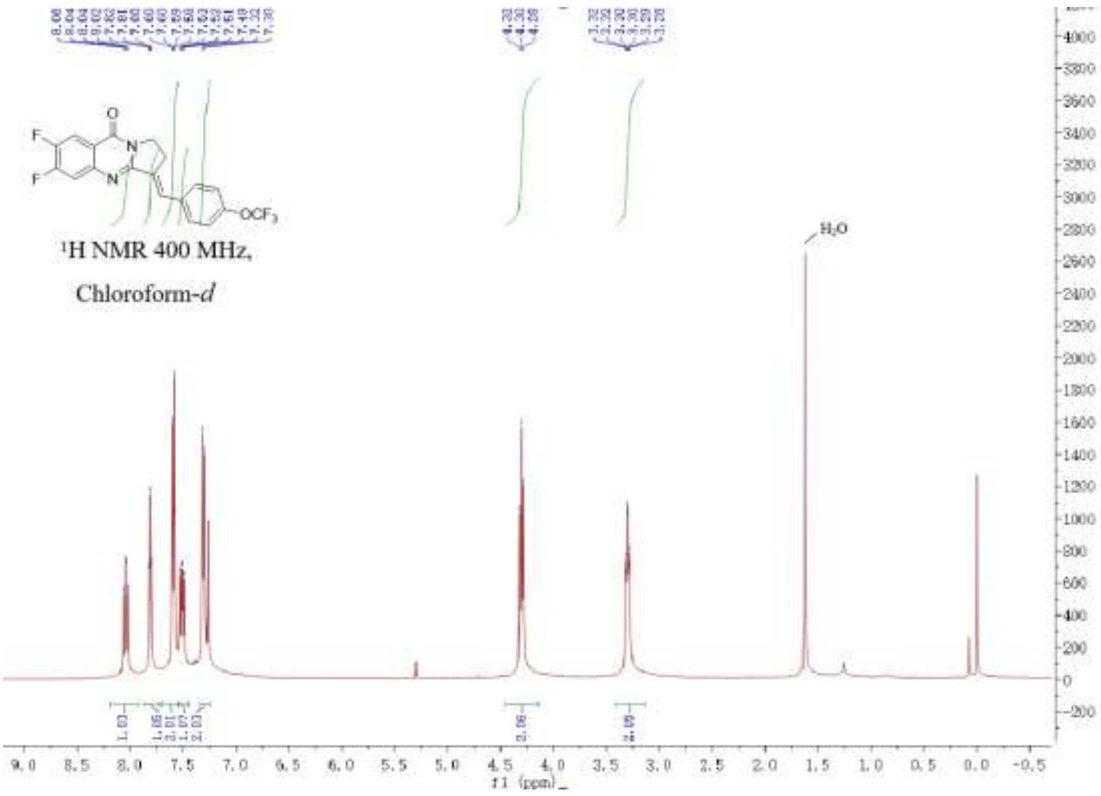

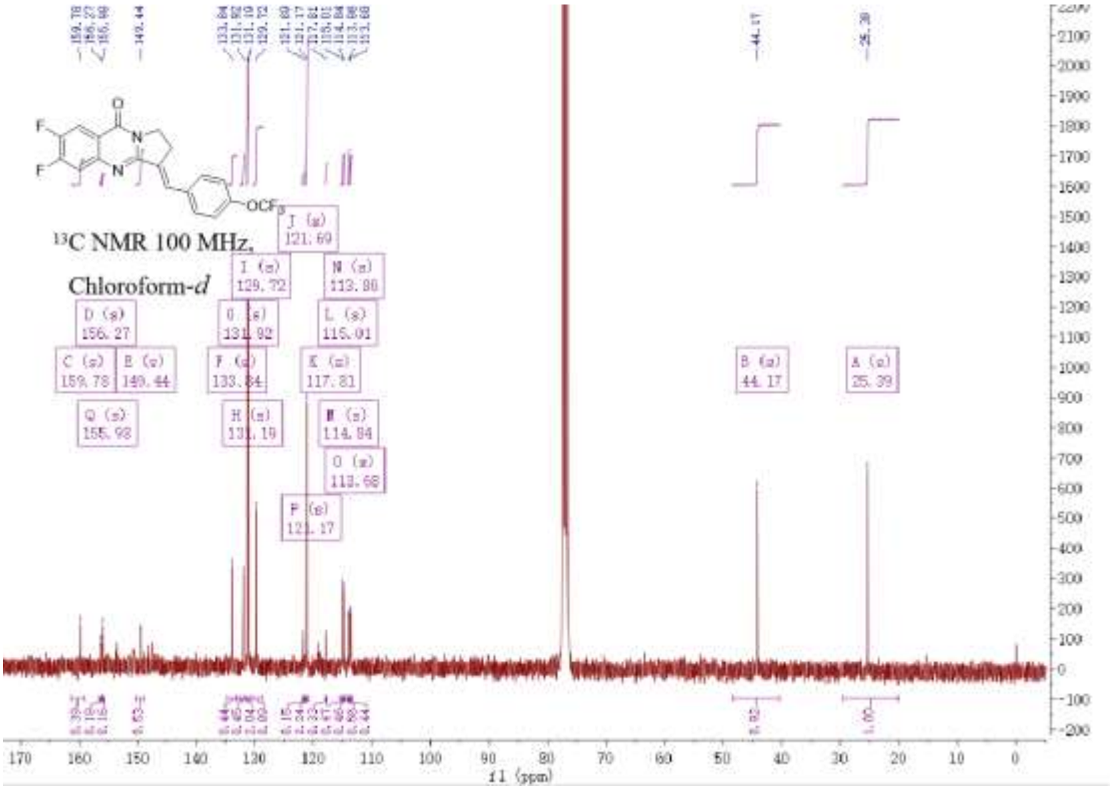

^{13}C NMR 100 MHz,
Chloroform-*d*
f1 (ppm)

Generic Display Report

Analysis Info

Analysis Name	D:\Data\yangy\new\YANGCHENGJIE210511_3_3_01_39144.d	Acquisition Date	5/11/2021 11:59:17 AM
Method	POS_100-1200_For LC.m	Operator	LZU
Sample Name	YANGCHENGJIE210511_3	Instrument	micrOTOF
Comment			

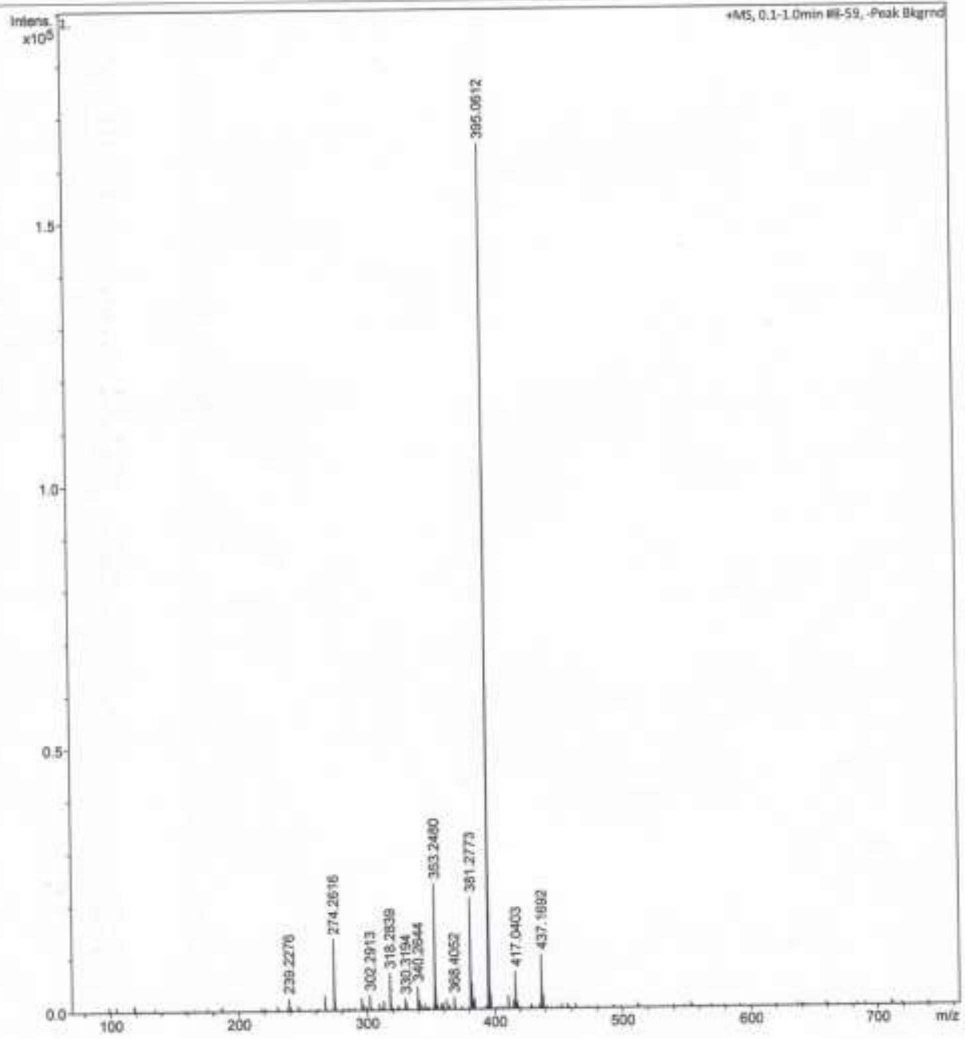

Bruker Compass DataAnalysis 4.1 printed: 5/11/2021 3:17:41 PM by: LZU Page 1 of 1

62

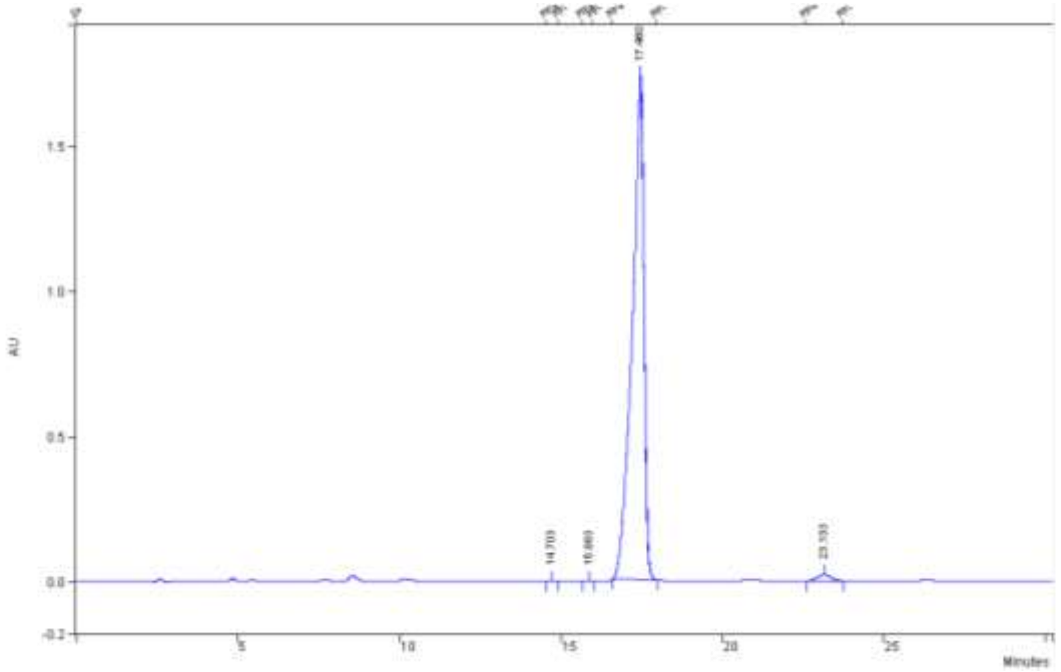

Peak No.	Peak Name	Result ()	Ret. Time (min)	Time Offset (min)	Area (counts)	Sep. Code	Width 1/2 (sec)	Status Codes
1		0.0113	14.703	0.000	49950	BB	13.3	
2		0.0023	15.883	0.000	10163	BB	13.6	
3		98.5738	17.460	0.000	435823776	BB	22.5	
4		1.4126	23.133	0.000	6245554	BB	27.6	
	Totals:	100.0000		0.000	442129443			

Figure S8. ^{1}H NMR, ^{13}C NMR, HRESIMS, HPLC spectra of compound **8**.

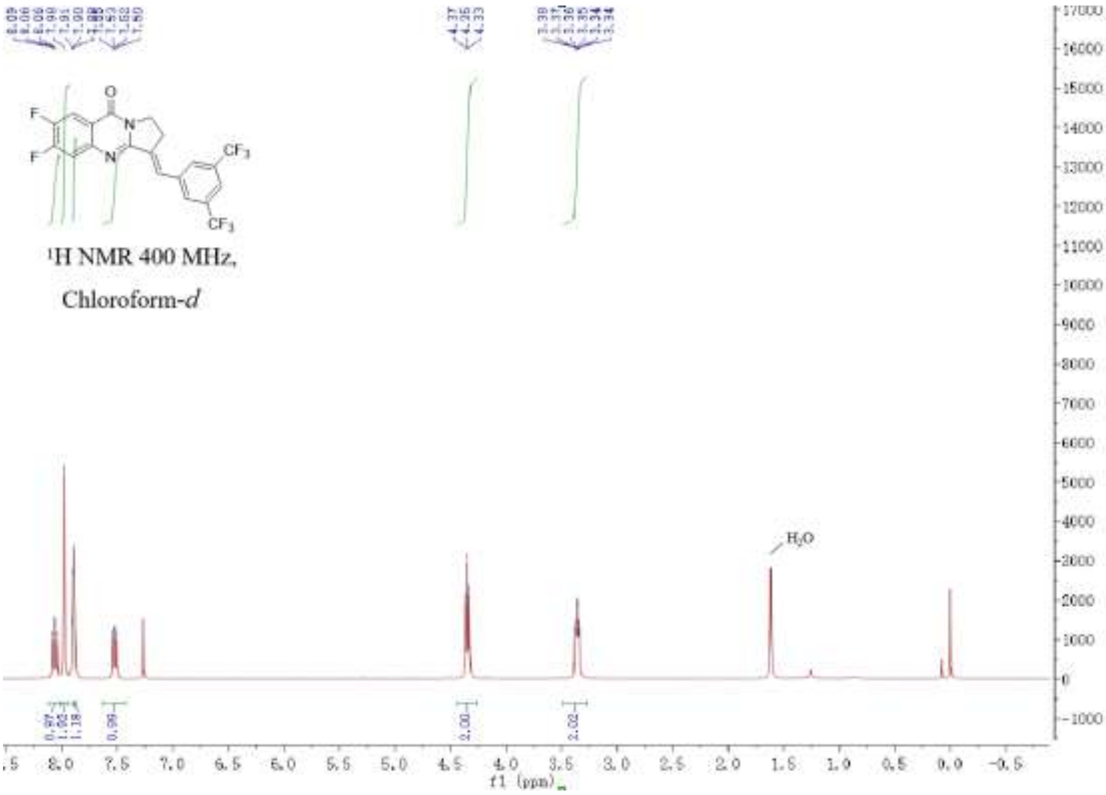

68

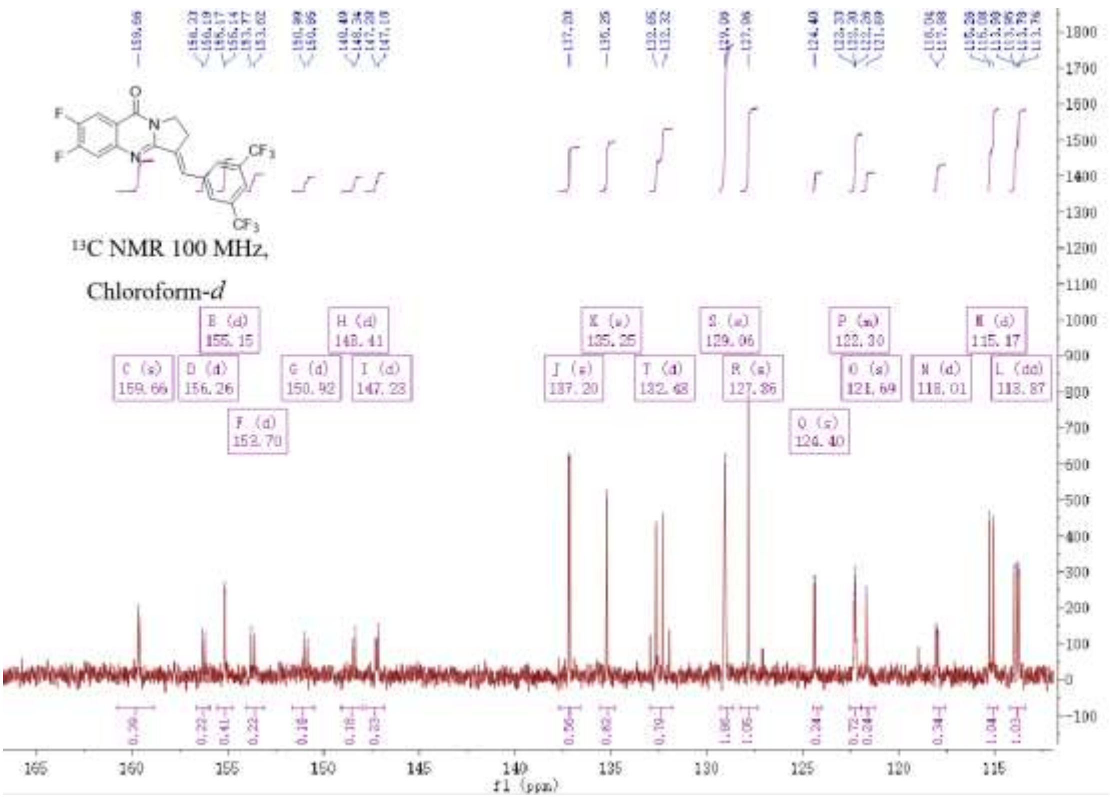

Generic Display Report

**Analysis Info**		Acquisition Date	5/11/2021 12:02:20 PM
Analysis Name	D:\Data\yangy\new\YANGCHENGJIE210511_4_4_01_39145.d		
Method	POS_100-1200_For LC.m	Operator	LZU
Sample Name	YANGCHENGJIE210511_4	Instrument	micrOTOF
Comment			

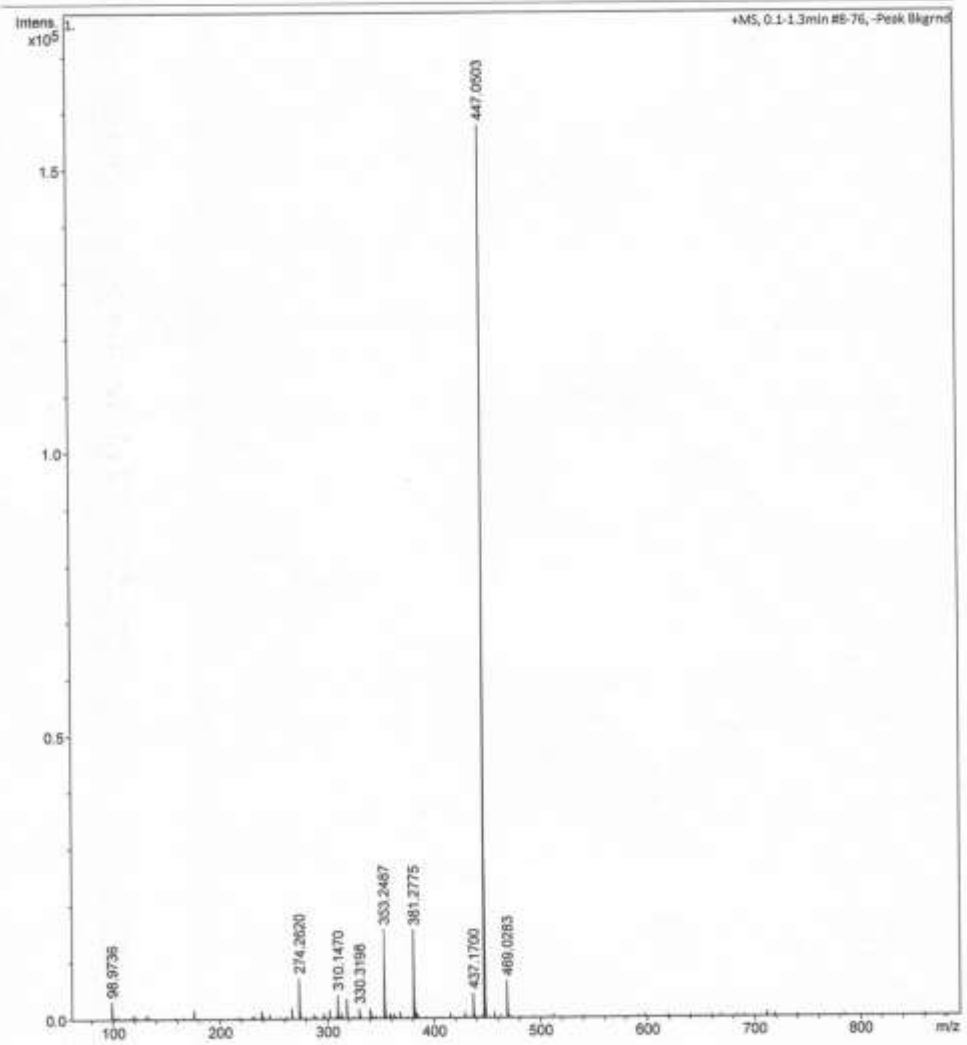

Bruker Compass DataAnalysis 4.1 printed: 5/11/2021 3:17:57 PM by: LZU Page 1 of 1

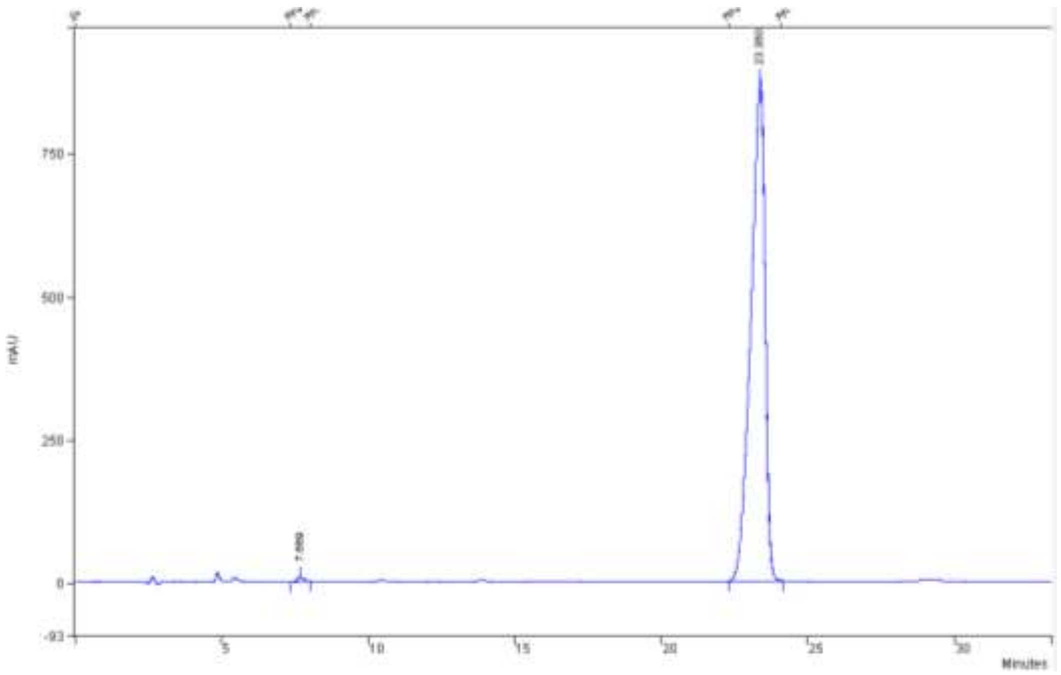

Peak No.	Peak Name	Result ()	Ret. Time (min)	Time Offset (min)	Area (counts)	Sep. Code	Width 1/2 (sec)	Status Codes
1		0.4553	7.669	0.000	1346659	BB	18.6	
2		99.5447	23.360	0.000	294207072	BB	31.3	
	Totals:	100.0000		0.000	295552731			

Figure S9. ^{1}H NMR, ^{13}C NMR, HRESIMS, HPLC spectra of compound **9**.

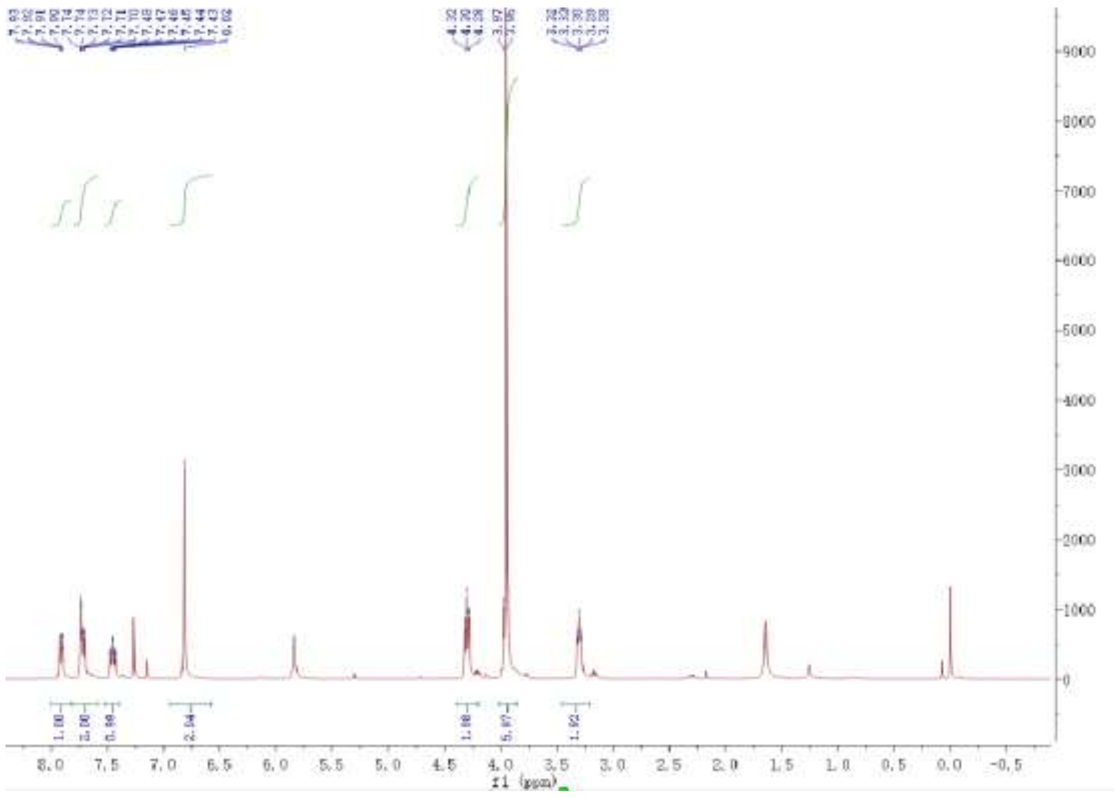

74

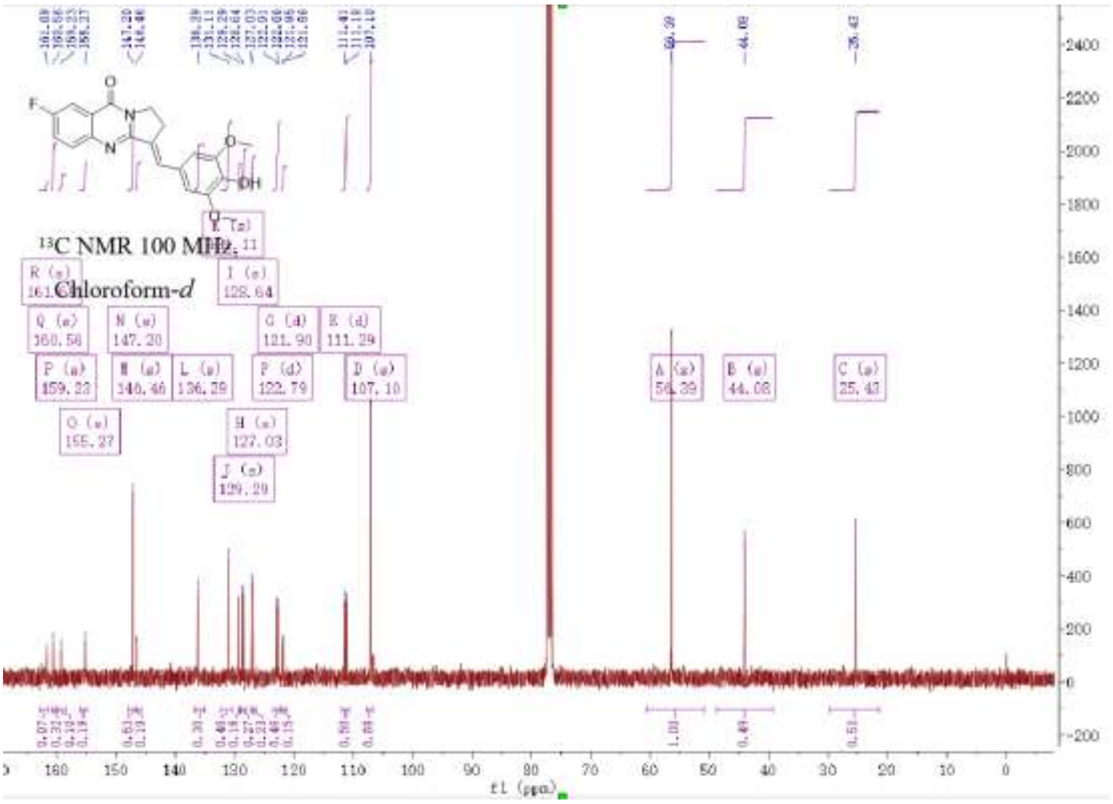

Generic Display Report

Analysis Info

Analysis Name	D:\Data\yangy\new\YANGCHENGJIE210511_5_5_01_39146.d	Acquisition Date	5/11/2021 12:05:27 PM
Method	POS_100-1200_For LC.m	Operator	LZU
Sample Name	YANGCHENGJIE210511_5	Instrument	micrOTOF
Comment			

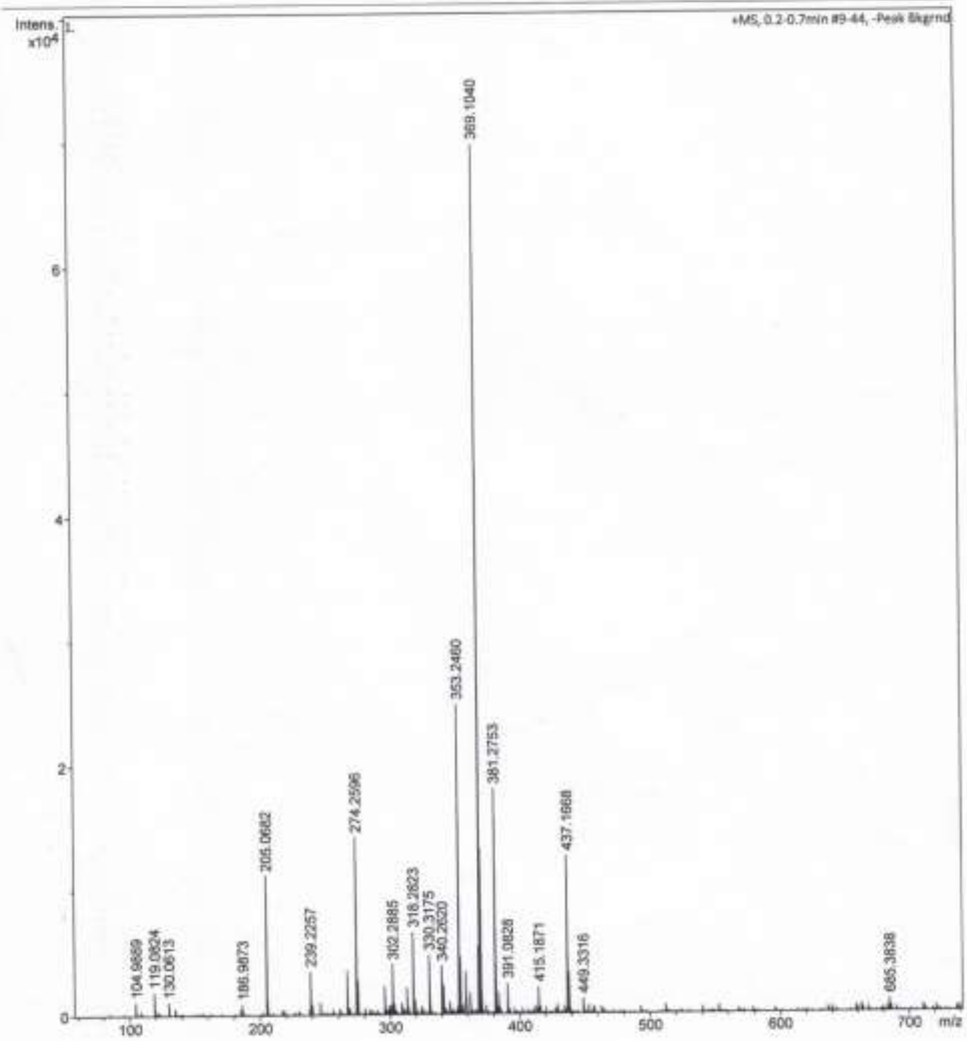

Bruker Compass DataAnalysis 4.1 printed: 5/11/2021 3:18:15 PM by: LZU Page 1 of 1

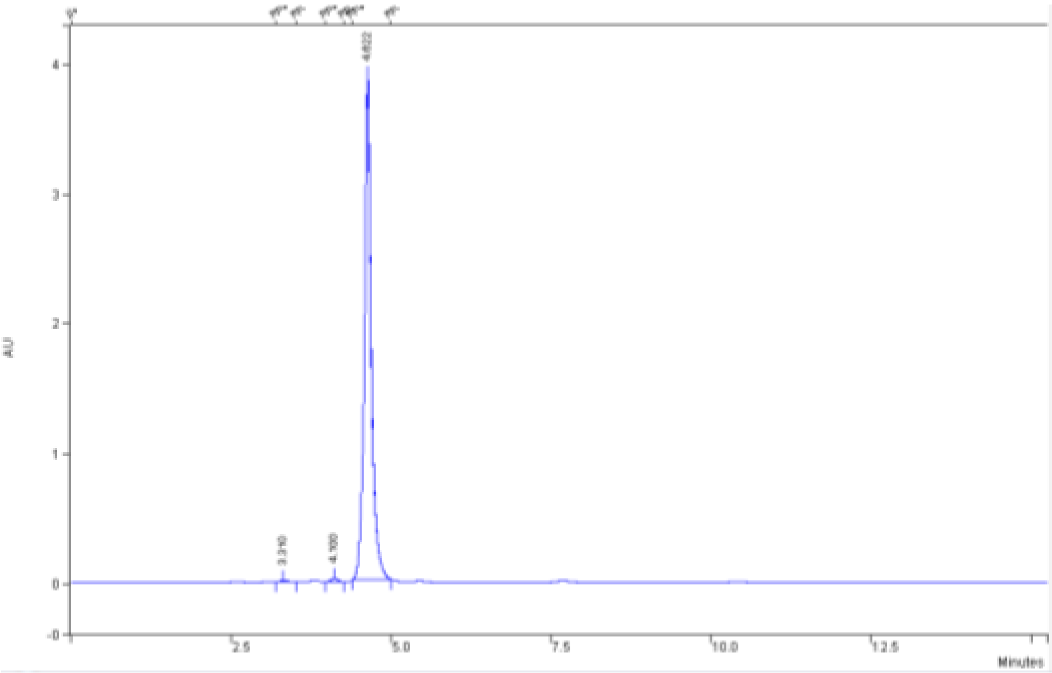

Peak No.	Peak Name	Result ()	Ret. Time (min)	Time Offset (min)	Area (counts)	Sep. Code	Width 1/2 (sec)	Status Codes
1		0.3427	3.310	0.000	1011954	BB	7.9	
2		0.6922	4.100	0.000	2044115	BB	8.1	
3		98.9651	4.622	0.000	292258112	BB	8.8	
	Totals:	100.0000		0.000	295314181			

Figure S10. ^{1}H NMR, ^{13}C NMR, HRESIMS, HPLC spectra of compound **10**.

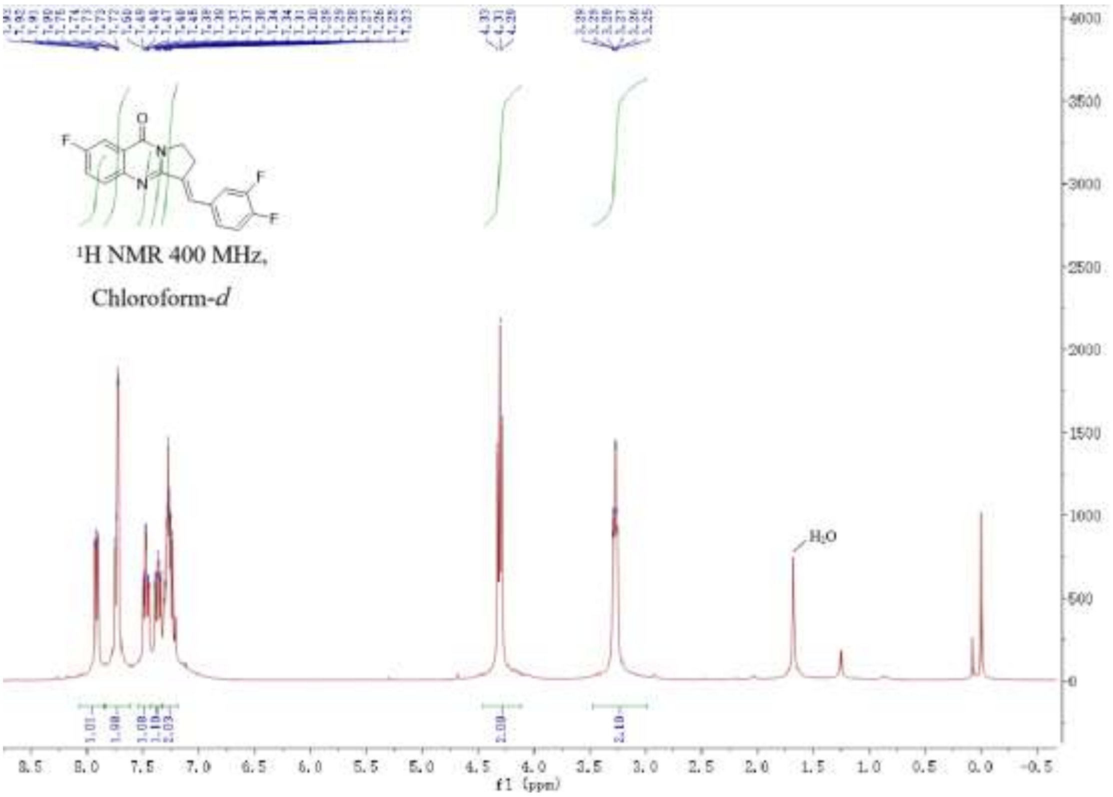

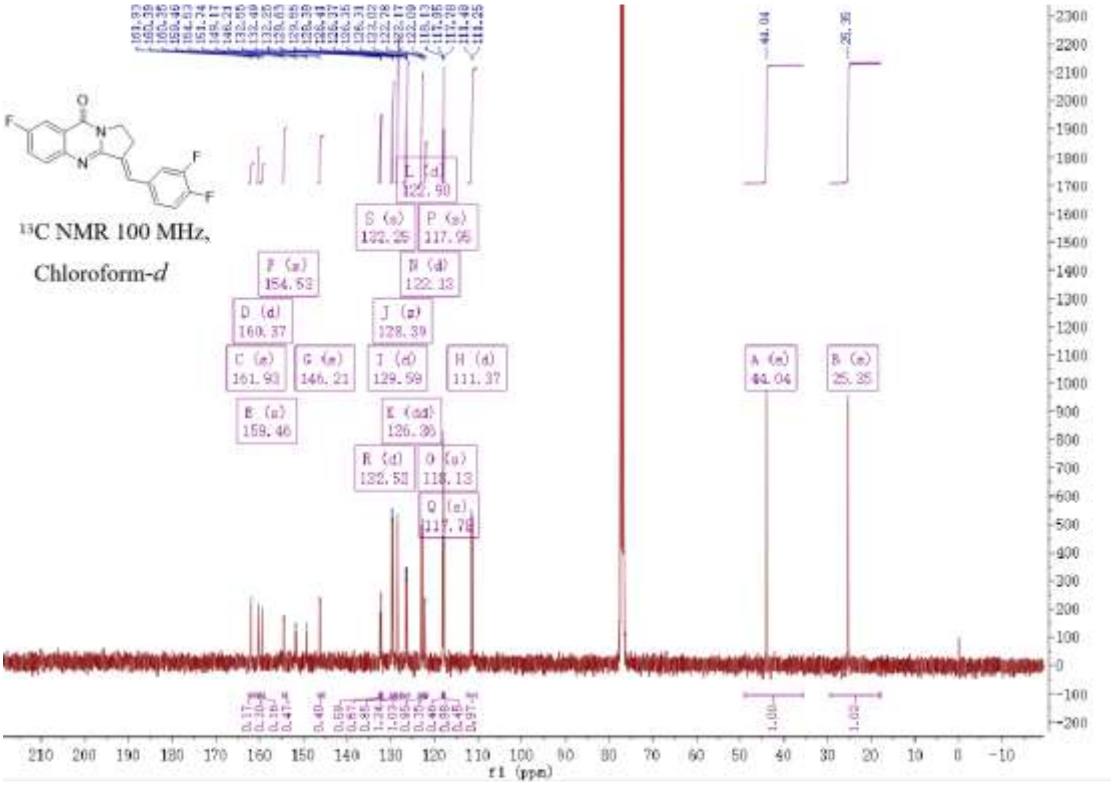
13C NMR 100 MHz,
Chloroform-d
f1 (ppm)

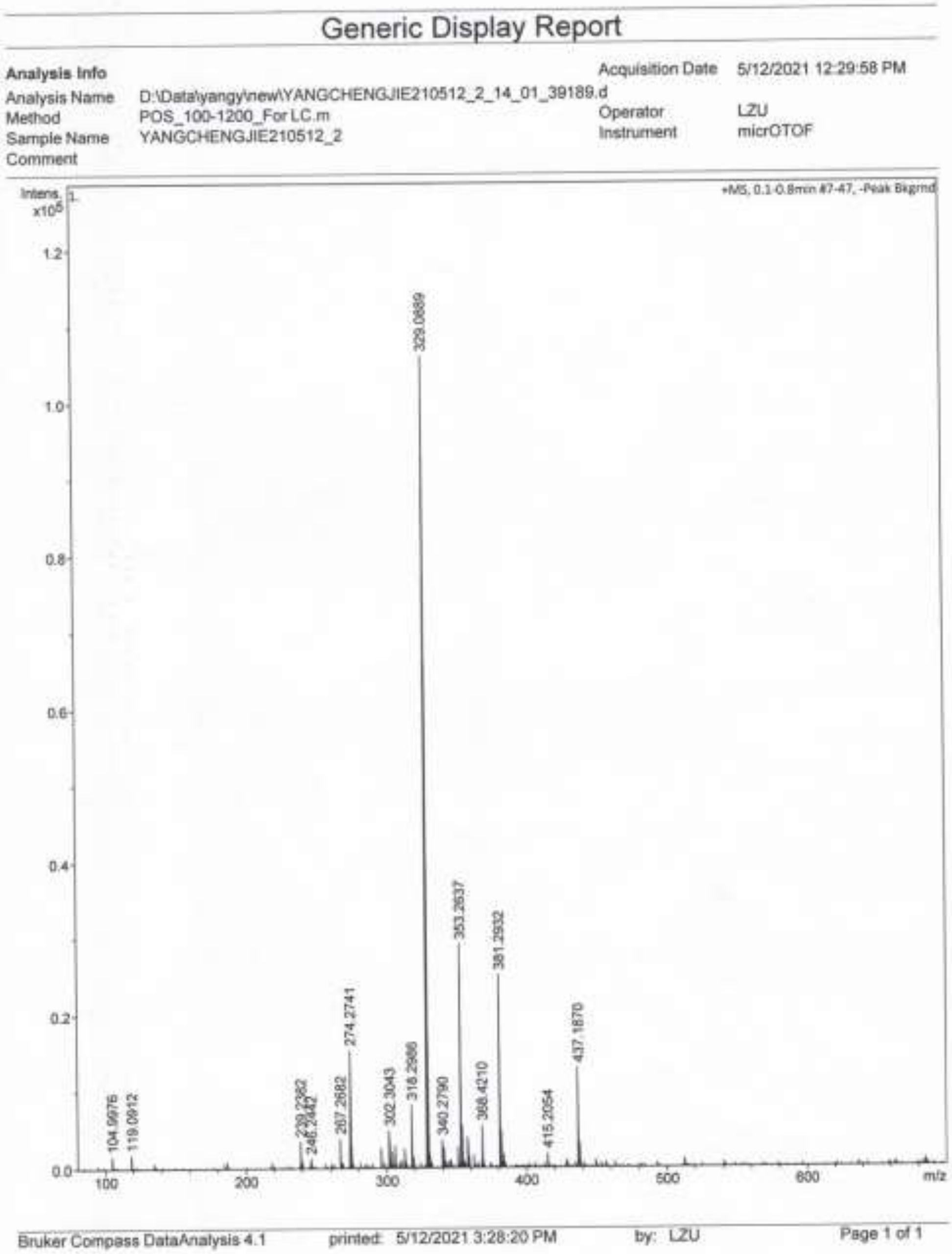
Generic Display Report
Analysis Info
Analysis Name D:\Data\yangy\new\YANGCHENGJIE210512_2_14_01_39189.d
Method POS_100-1200_For LC.m
Sample Name YANGCHENGJIE210512_2
Comment
Acquisition Date 5/12/2021 12:29:58 PM
Operator LZU
Instrument micrOTOF
+MS, 0.1-0.8min #7-47, -Peak Bkgrnd
Intens. x10^5
329.0889
353.2637
381.2932
274.2741
437.1870
318.2986
302.3043
340.2790
368.4210
415.2054
267.2682
239.2382
246.2442
104.9976
119.0912
m/z
Bruker Compass DataAnalysis 4.1 printed: 5/12/2021 3:28:20 PM by: LZU Page 1 of 1

82

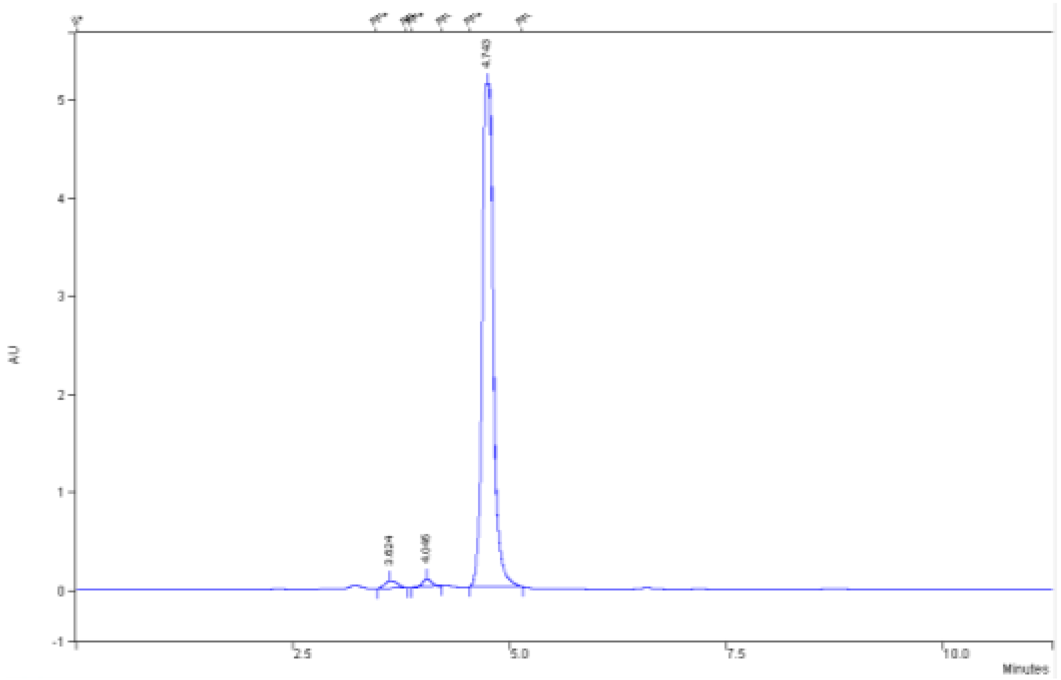

Peak No.	Peak Name	Result ()	Ret. Time (min)	Time Offset (min)	Area (counts)	Sep. Code	Width 1/2 (sec)	Status Codes
1		1.6262	3.624	0.000	7706391	BB	10.0	
2		1.2030	4.045	0.000	5701105	BB	7.0	
3		97.1708	4.743	0.000	460489088	BB	7.9	
	Totals:	100.0000		0.000	473996584			

Figure S12. ^{1}H NMR, ^{13}C NMR, HRESIMS, HPLC spectra of compound **12**.

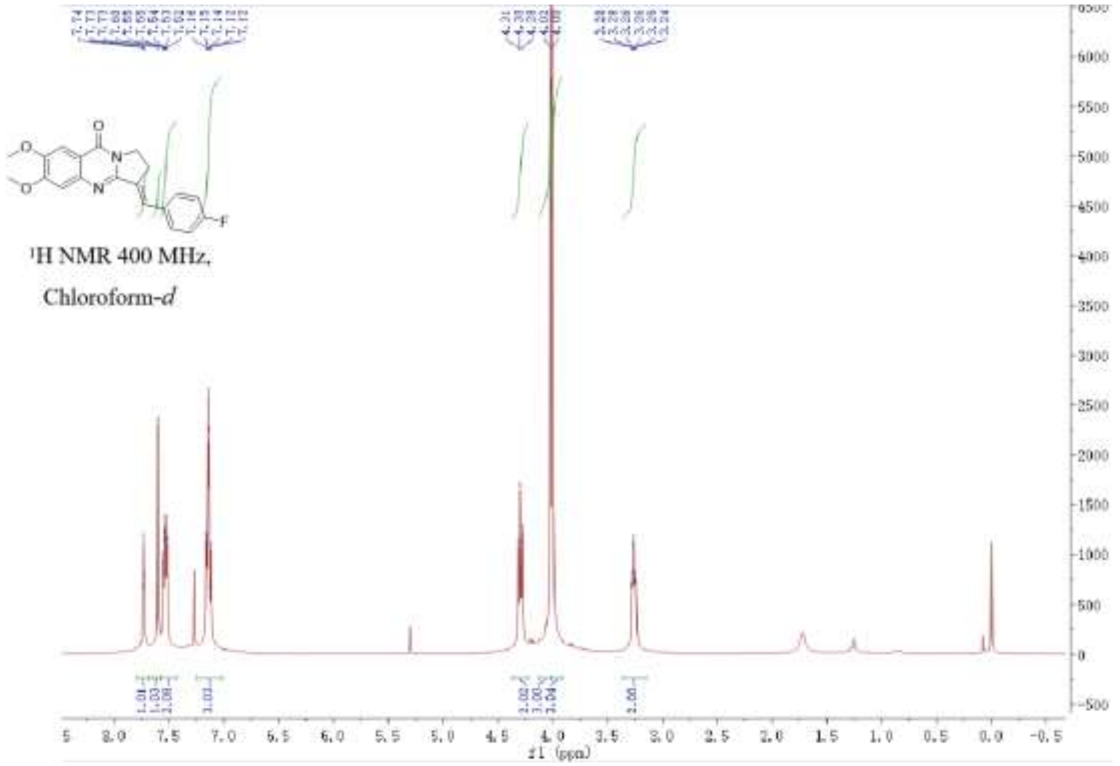

87

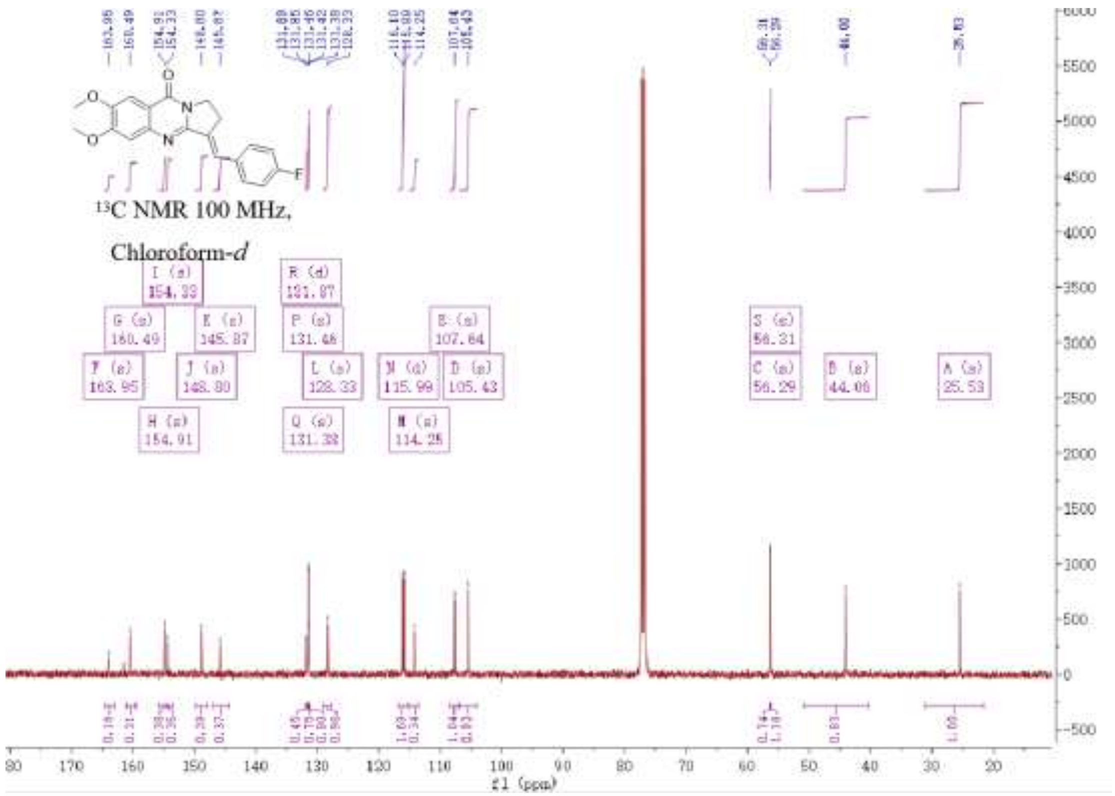

Generic Display Report

**Analysis Info**		Acquisition Date	5/12/2021 12:36:05 PM
Analysis Name	D:\Data\yangy\new\YANGCHENGJIE210512_4_16_01_39191.d		
Method	POS_100-1200_For LC.m	Operator	LZU
Sample Name	YANGCHENGJIE210512_4	Instrument	micrOTOF
Comment			

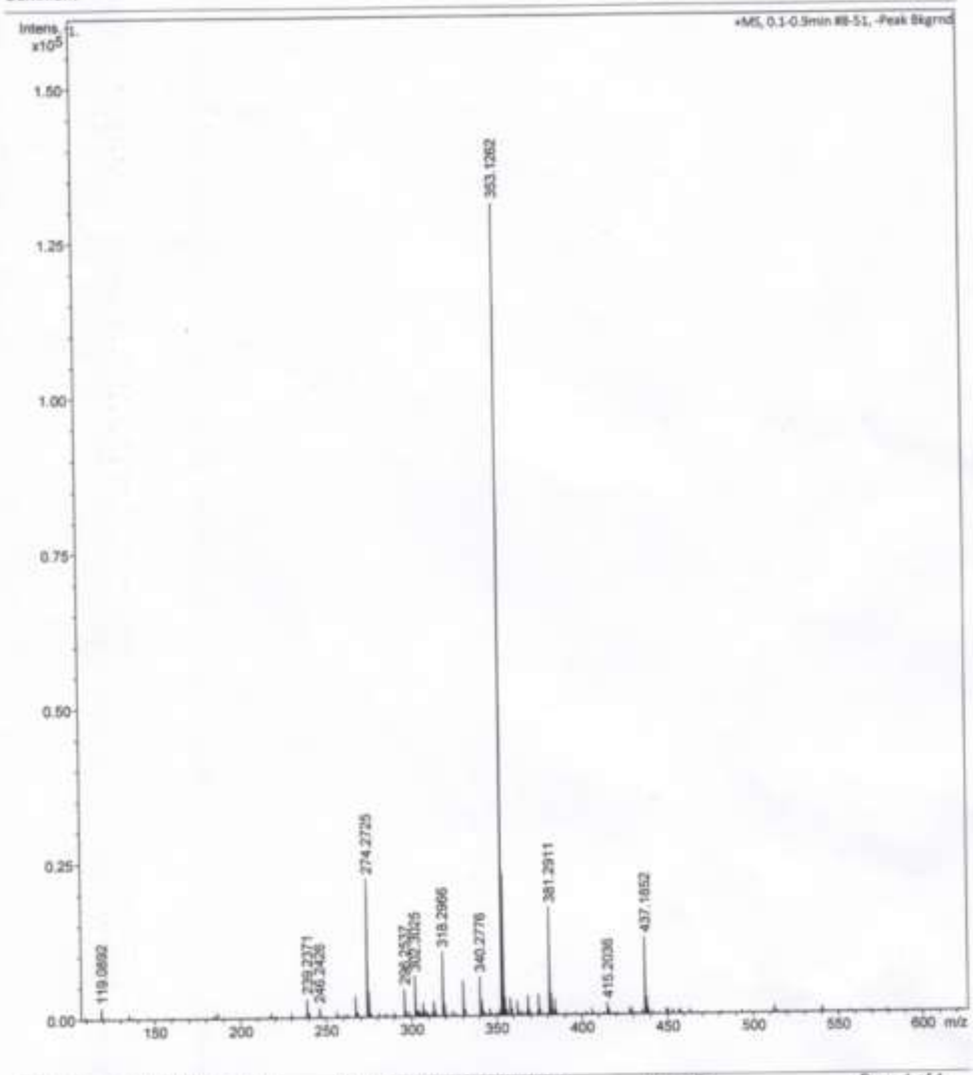

Bruker Compass DataAnalysis 4.1 printed: 5/12/2021 3:28:47 PM by: LZU Page 1 of 1

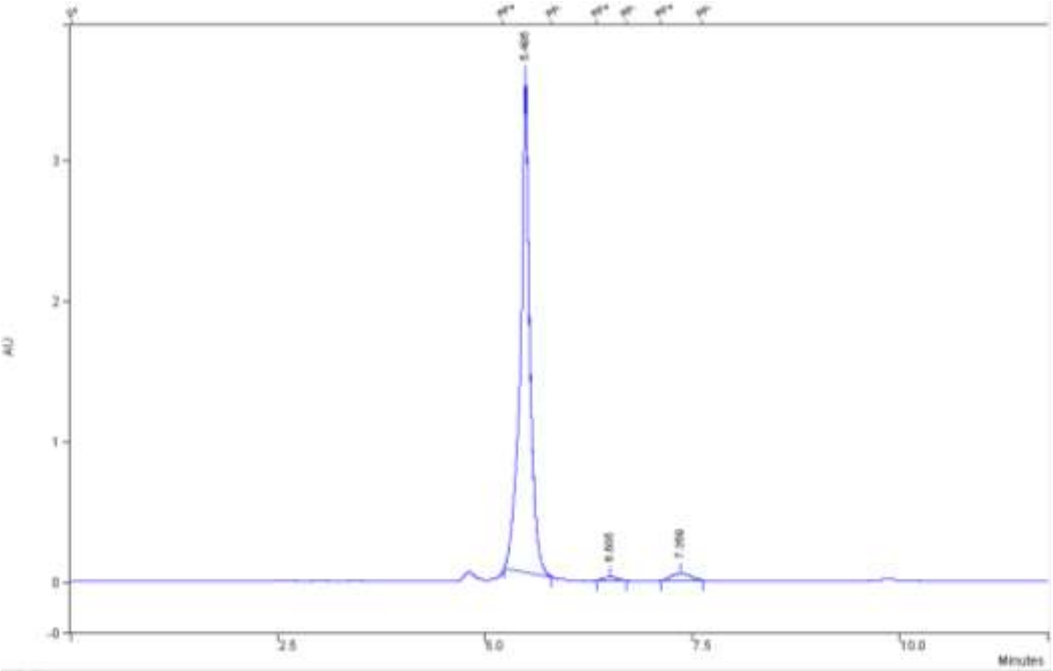

Peak No.	Peak Name	Result ()	Ret. Time (min)	Time Offset (min)	Area (counts)	Sep. Code	Width 1/2 (sec)	Status Codes
1		96.4145	5.468	0.000	281340512	BB	8.6	
2		0.9242	6.505	0.000	2696706	BB	10.8	
3		2.6613	7.359	0.000	7765895	BB	15.6	
	Totals:	100.0000		0.000	291803113			

Figure S14. ^{1}H NMR, ^{13}C NMR, HRESIMS, HPLC spectra of compound **14**.

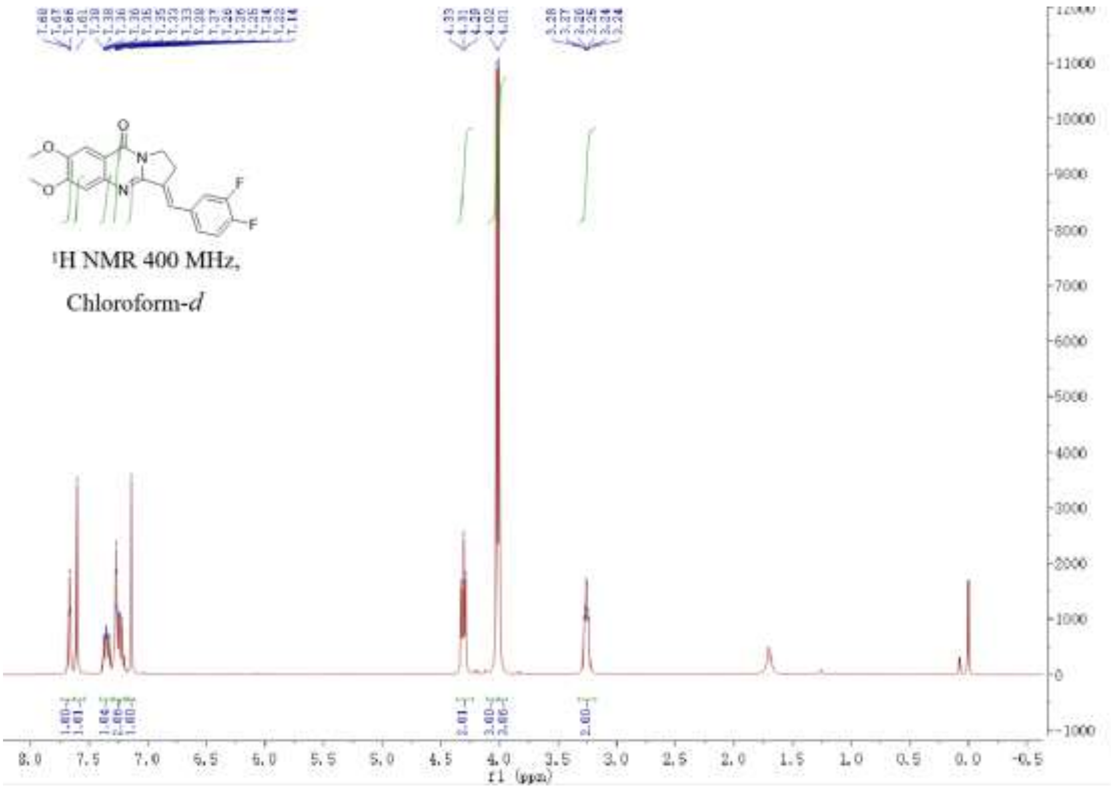

93

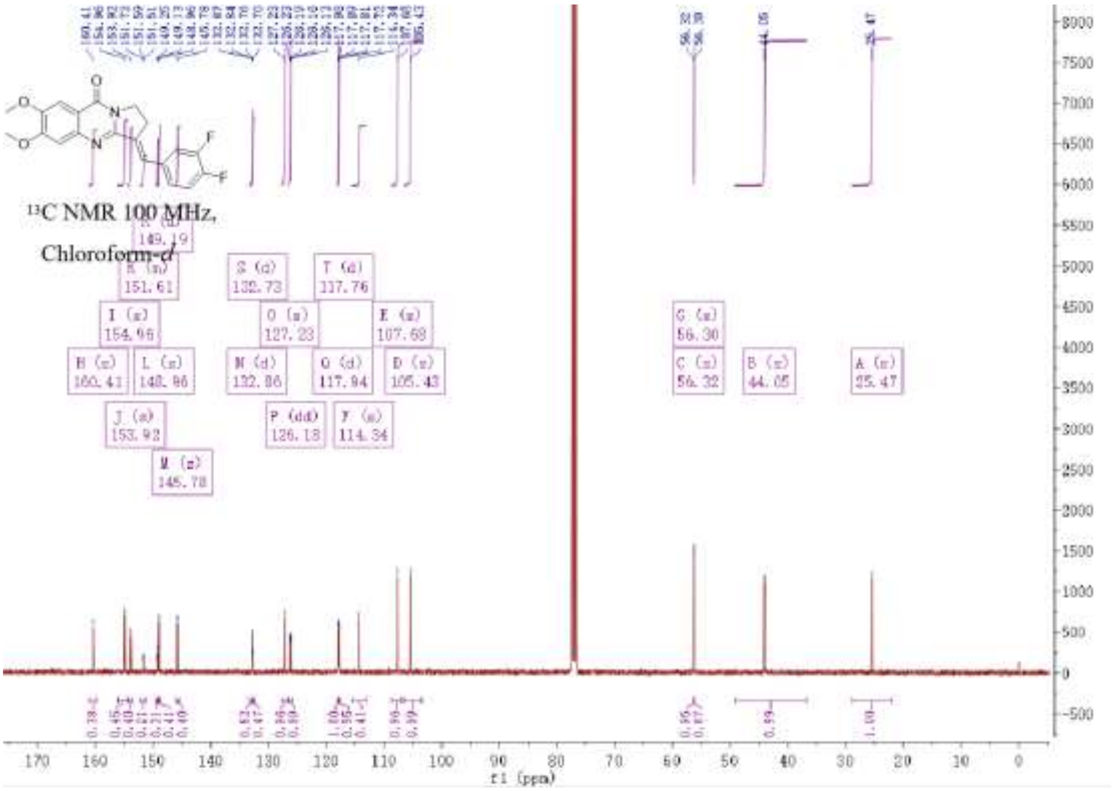

Generic Display Report

**Analysis Info**		Acquisition Date	5/12/2021 12:39:11 PM
Analysis Name	D:\Data\yangy\new\YANGCHENGJIE210512_5_17_01_39192.d		
Method	POS_100-1200_For LC.m	Operator	LZU
Sample Name	YANGCHENGJIE210512_5	Instrument	micrOTOF
Comment			

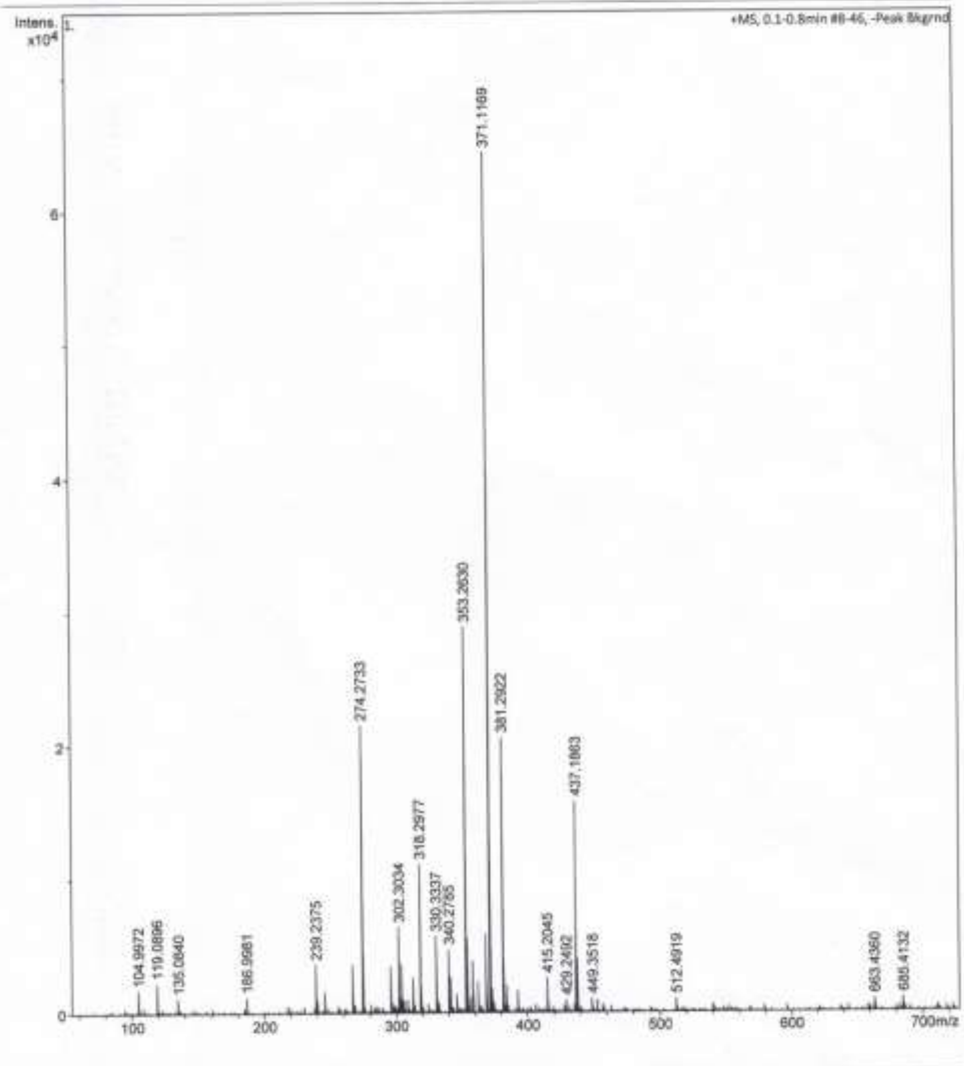

Bruker Compass DataAnalysis 4.1 printed: 5/12/2021 3:29:00 PM by: LZU Page 1 of 1

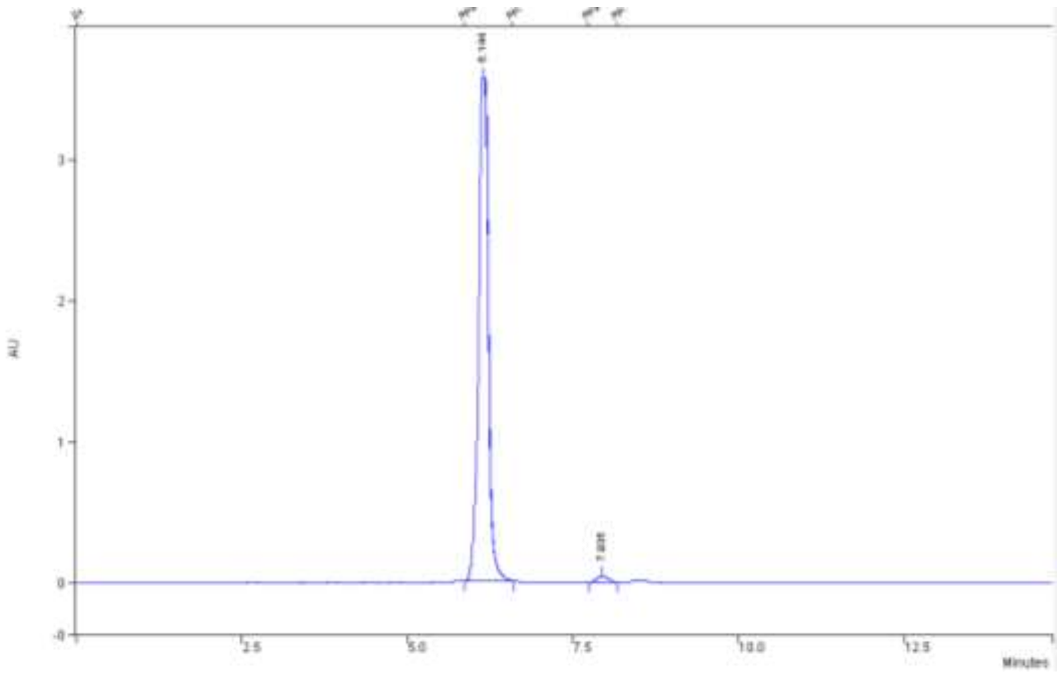

Peak No.	Peak Name	Result ()	Ret. Time (min)	Time Offset (min)	Area (counts)	Sep. Code	Width 1/2 (sec)	Status Codes
1		98.6360	6.144	0.000	371050944	BB	9.0	
2		1.3640	7.935	0.000	5131135	BB	11.5	
	Totals:	100.0000		0.000	376182079			

Figure S15. ^{1}H NMR, ^{13}C NMR, HRESIMS, HPLC spectra of compound **15**.

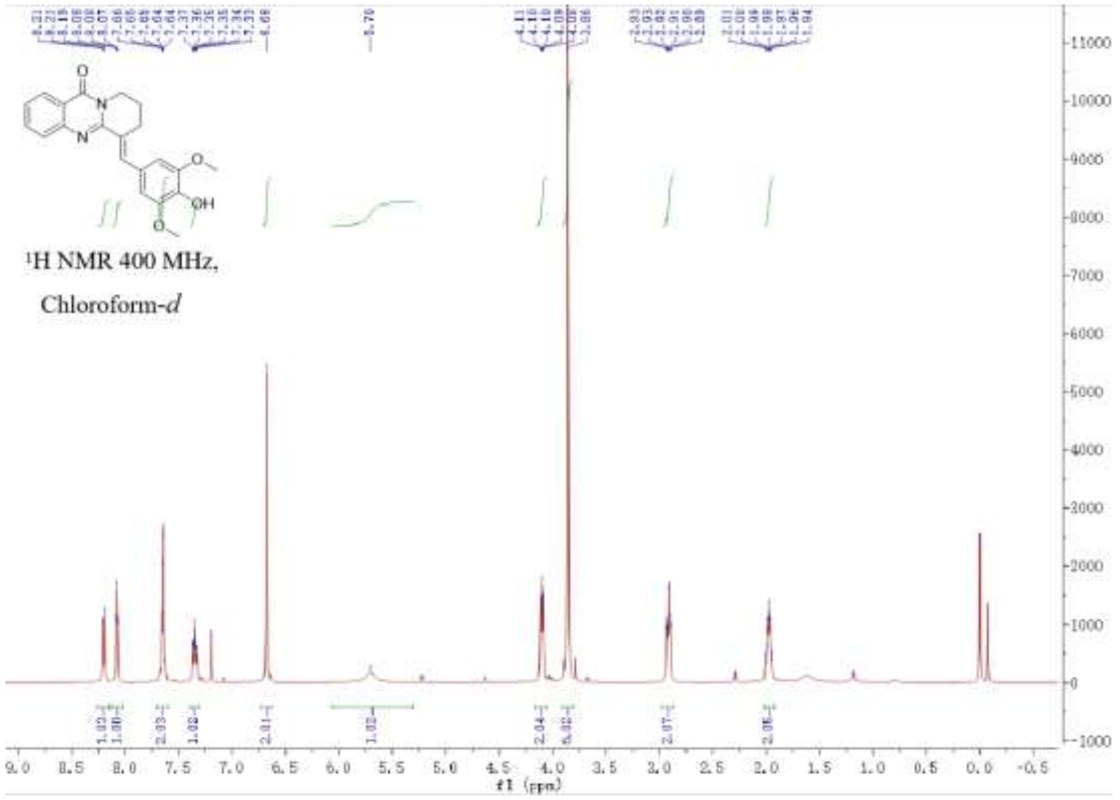

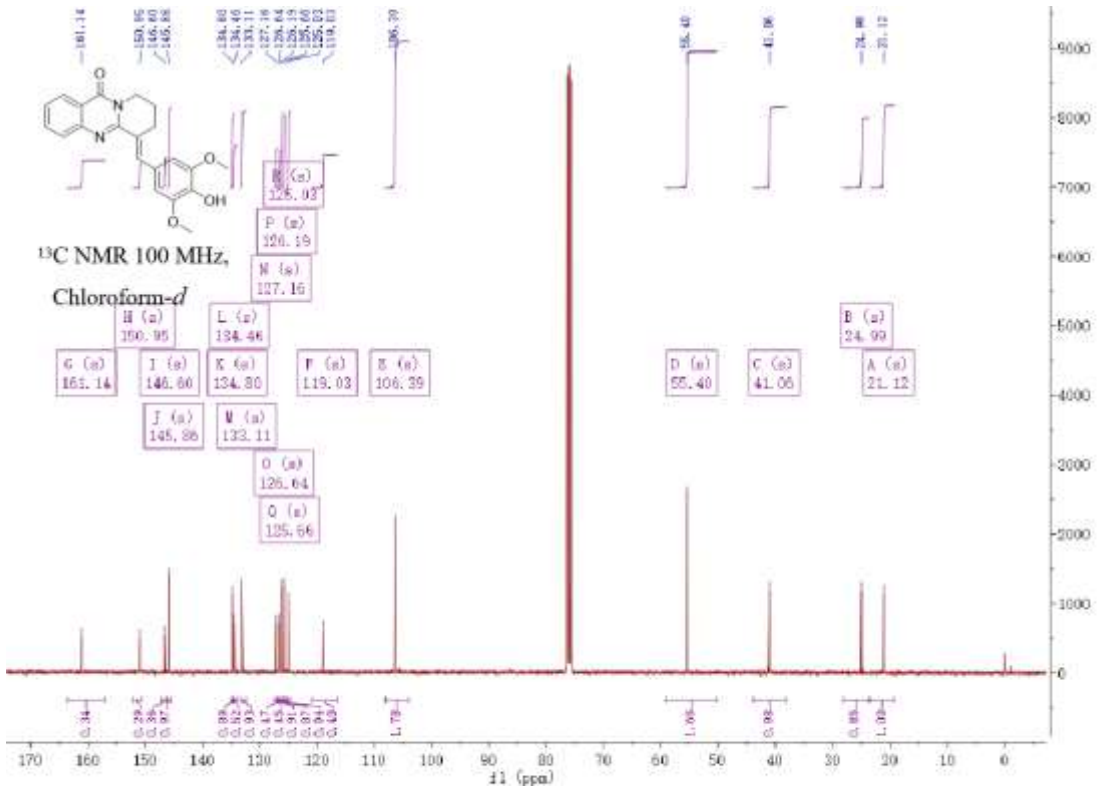
13C NMR 100 MHz,
Chloroform-d
G (s) 161.14
H (s) 150.95
I (s) 146.60
J (s) 145.86
K (s) 134.80
L (s) 134.46
M (s) 133.11
N (s) 127.16
O (s) 126.64
P (s) 126.19
Q (s) 125.66
R (s) 125.03
F (s) 119.03
E (s) 106.39
D (s) 55.40
C (s) 41.06
B (s) 24.99
A (s) 21.12
f1 (ppm)

Generic Display Report

**Analysis Info**		Acquisition Date	5/18/2021 1:05:15 PM
Analysis Name	D:\Data\yangy\new\YANGCHENGJIE210518_2_20_01_39318.d		
Method	POS_100-1200_For LC.m	Operator	LZU
Sample Name	YANGCHENGJIE210518_2	Instrument	micrOTOF
Comment			

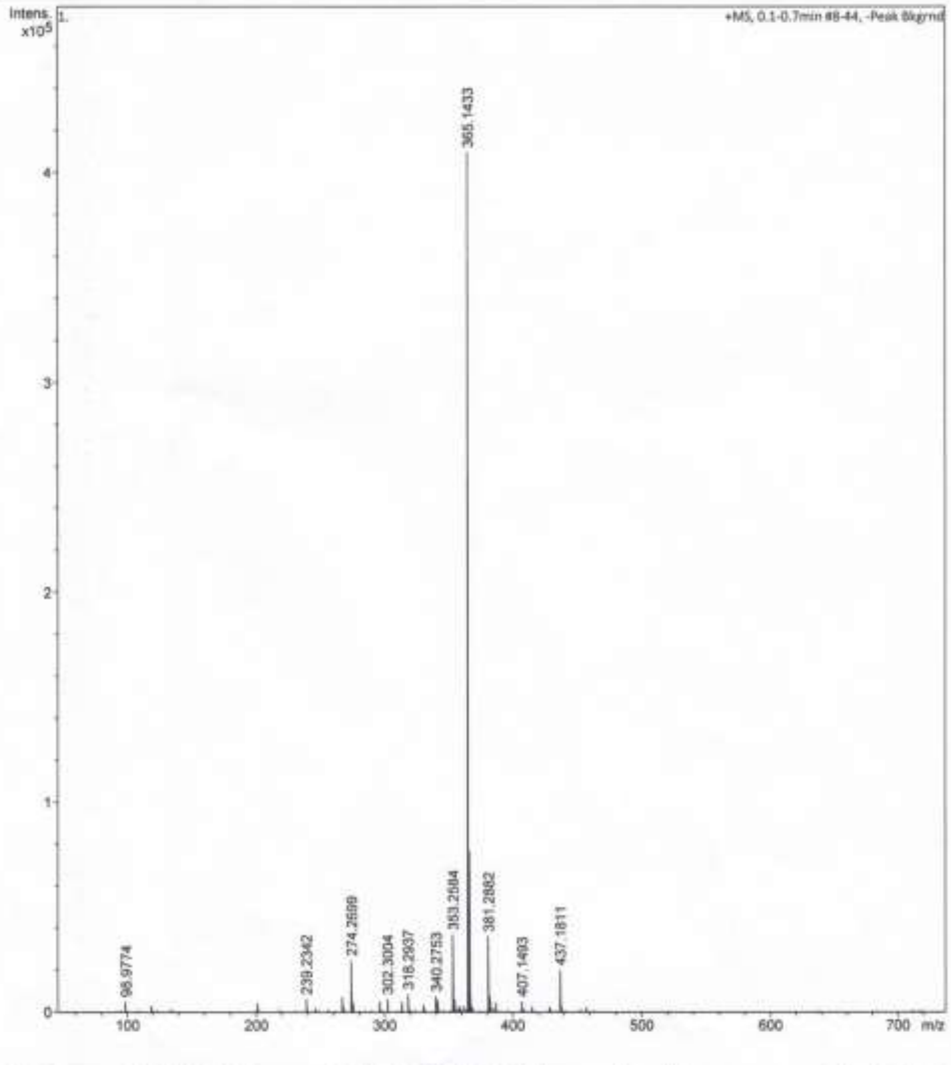

Bruker Compass DataAnalysis 4.1 printed: 5/18/2021 3:35:00 PM by: LZU Page 1 of 1

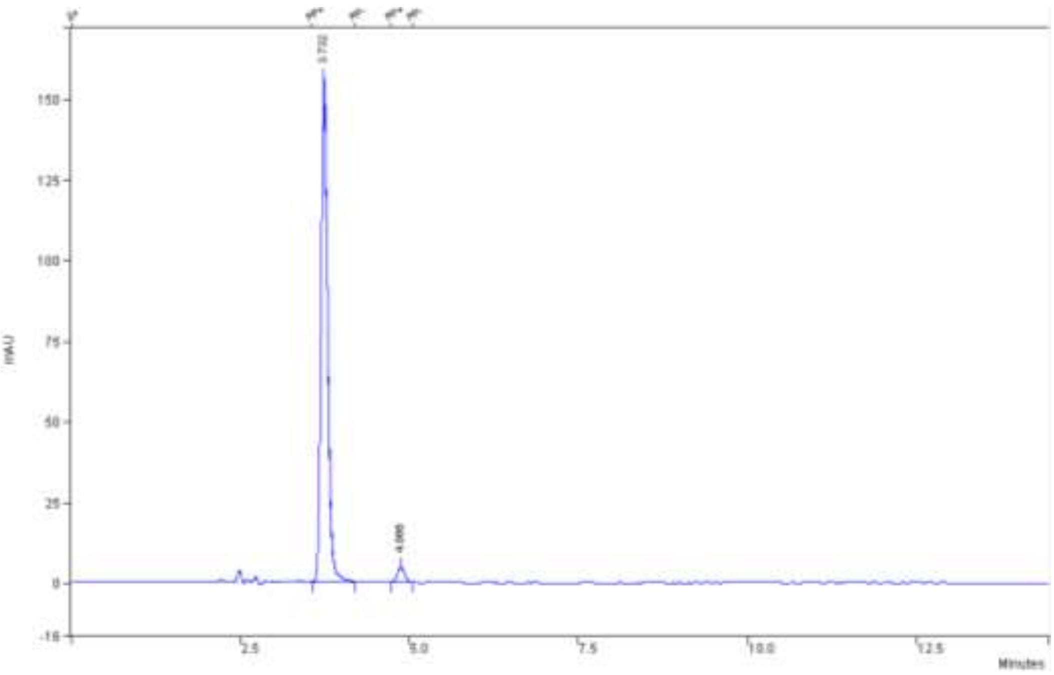

Peak No.	Peak Name	Result ()	Ret. Time (min)	Time Offset (min)	Area (counts)	Sep. Code	Width 1/2 (sec)	Status Codes
1		96.9504	3.732	0.000	10564768	BB	6.1	
2		3.0496	4.866	0.000	332320	BB	7.2	
	Totals:	100.0000		0.000	10897088			

Figure S17. ^{1}H NMR, ^{13}C NMR, HRESIMS, HPLC spectra of compound **17**.

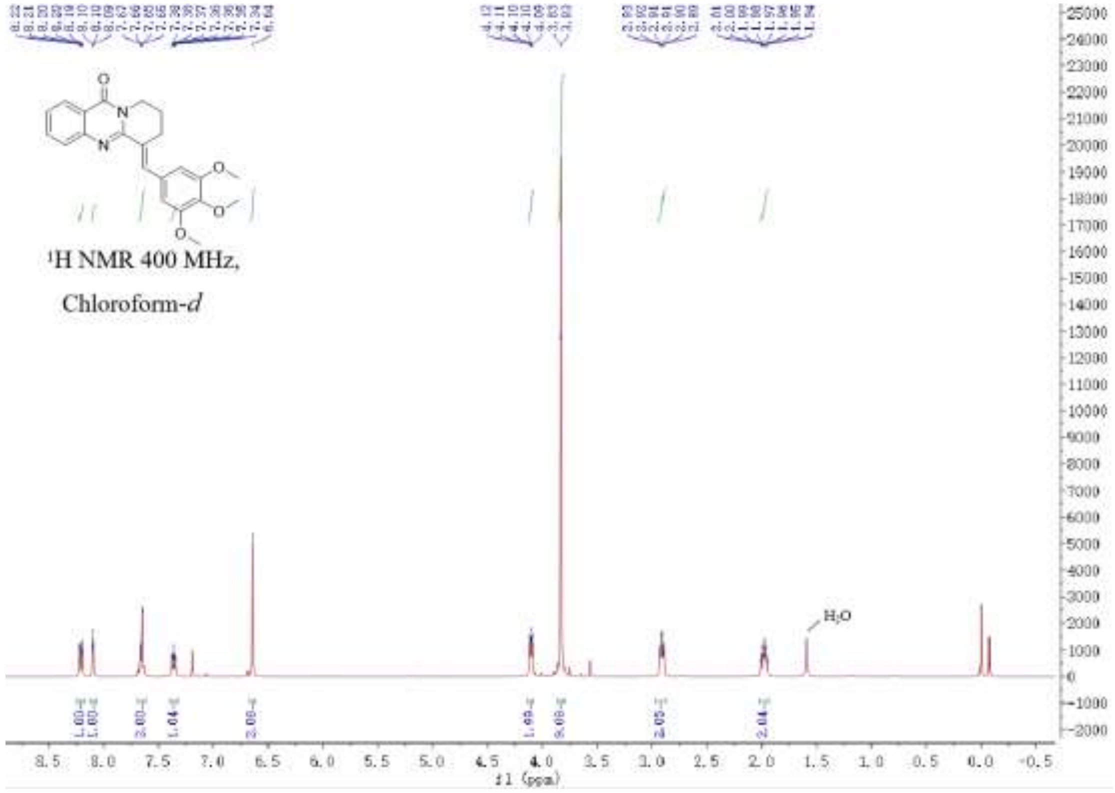

106

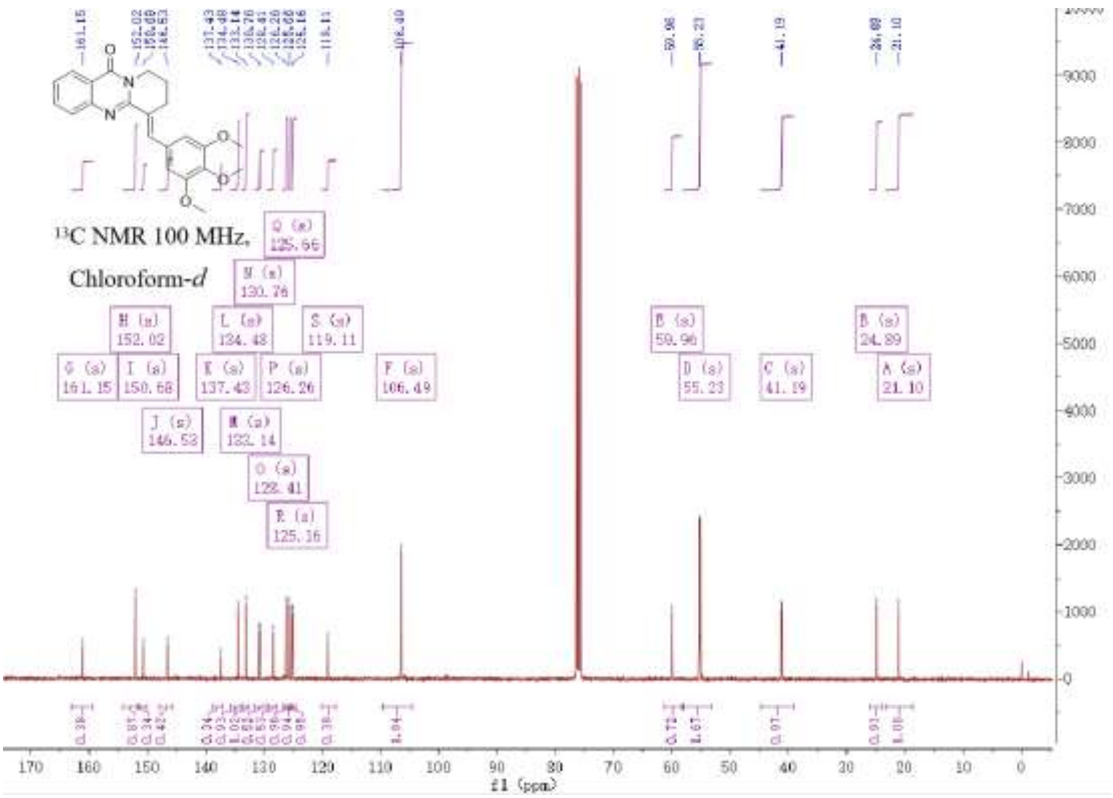

Generic Display Report

**Analysis Info**		Acquisition Date	5/18/2021 1:08:22 PM
Analysis Name	D:\Data\yangy\new\YANGCHENGJIE210518_3_21_01_39319.d		
Method	POS_100-1200_For LC.m	Operator	LZU
Sample Name	YANGCHENGJIE210518_3	Instrument	micrOTOF
Comment			

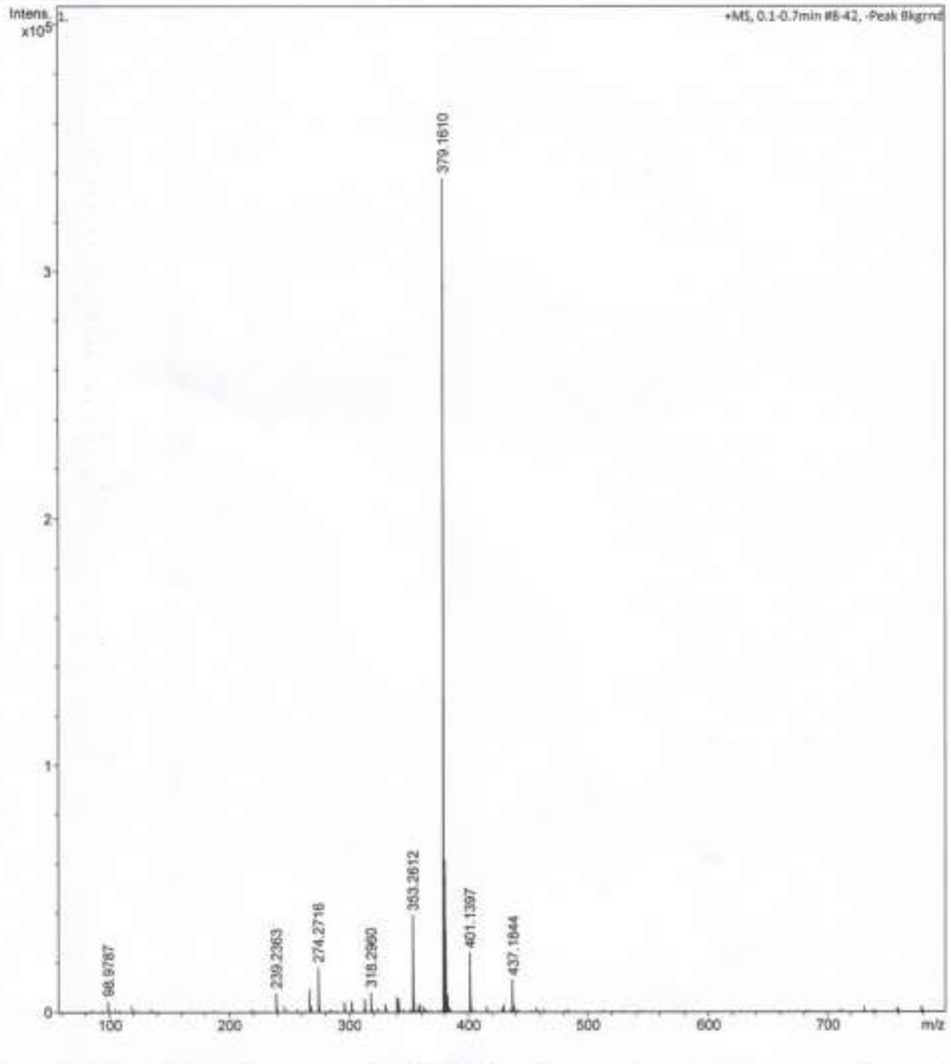

Bruker Compass DataAnalysis 4.1 printed: 5/18/2021 3:35:18 PM by: LZU Page 1 of 1

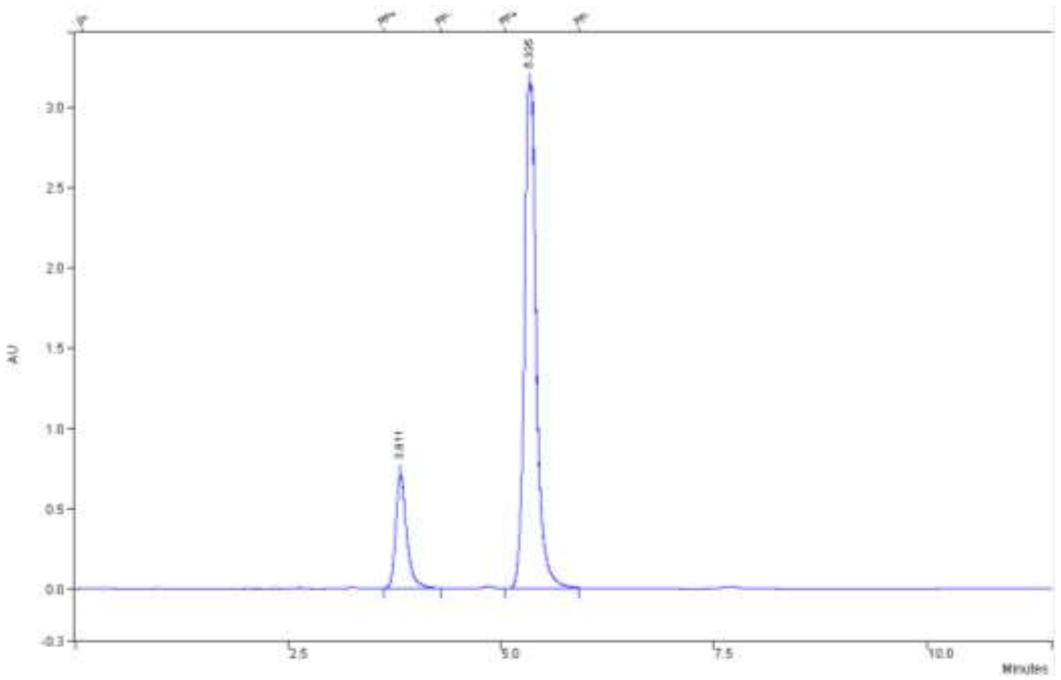

Peak No.	Peak Name	Result ()	Ret. Time (min)	Time Offset (min)	Area (counts)	Sep. Code	Width 1/2 (sec)	Status Codes
1		16.9125	3.811	0.000	62729840	BB	7.7	
2		83.0875	5.336	0.000	308178240	BB	8.2	
	Totals:	100.0000		0.000	370908080			

Figure S18. ^{1}H NMR, ^{13}C NMR, HRESIMS, HPLC spectra of compound **18**.

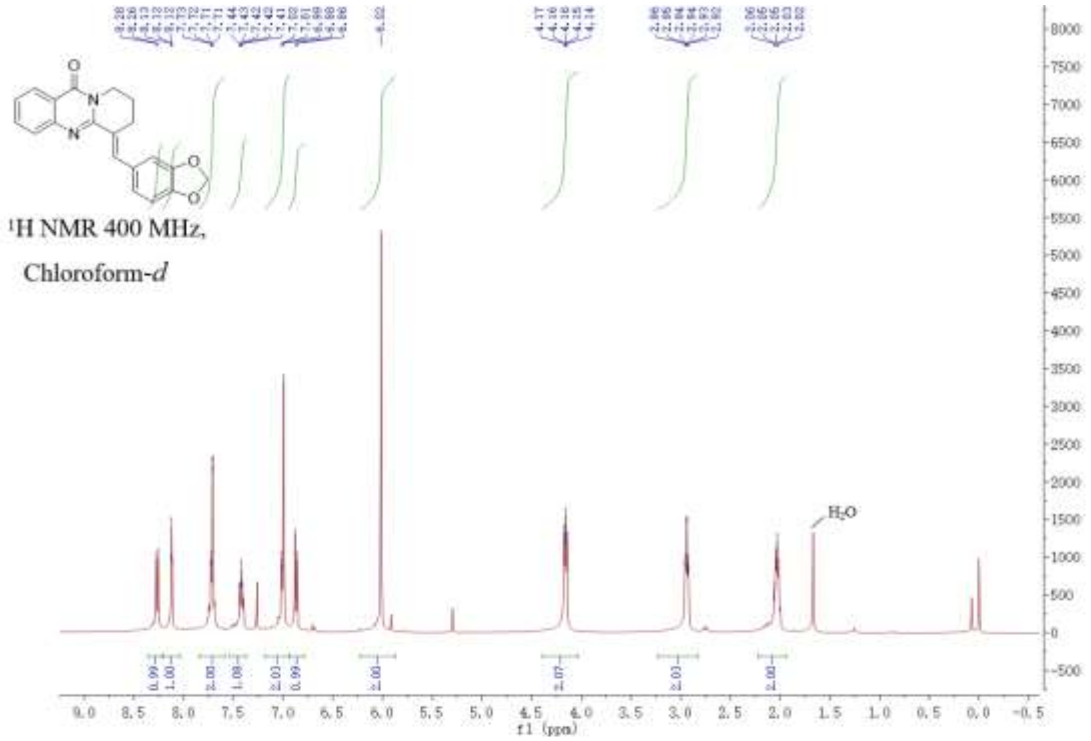

112

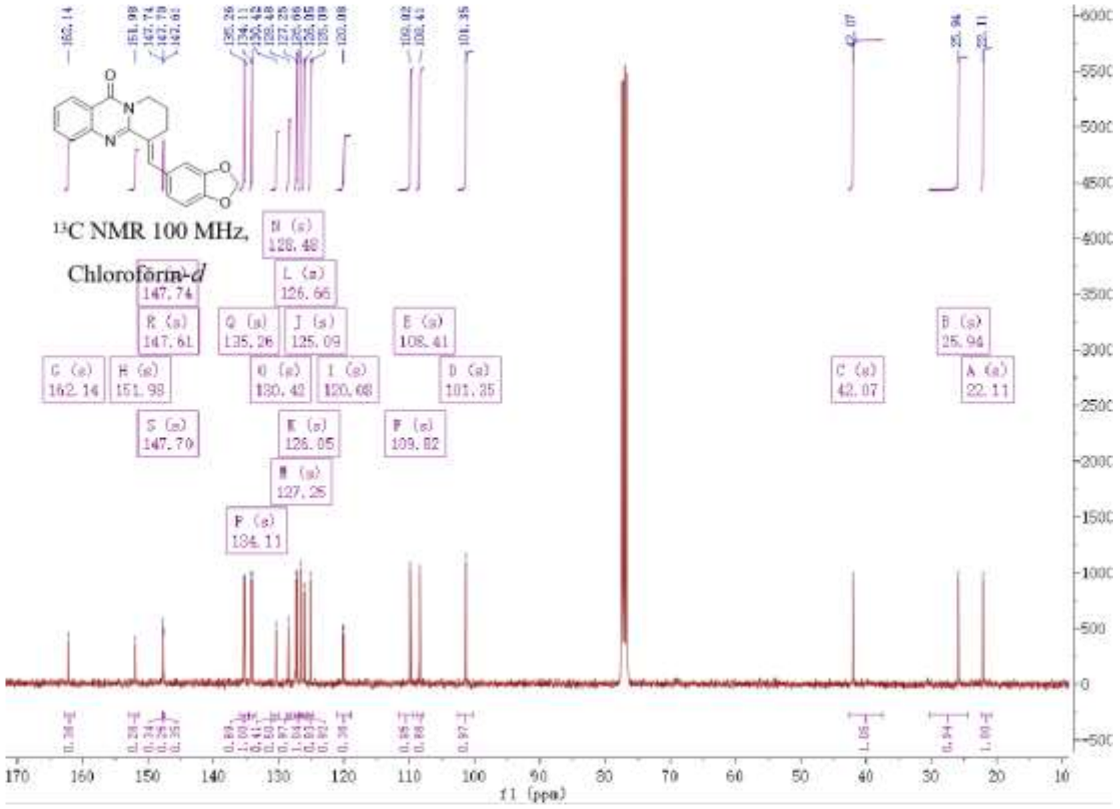

Generic Display Report

**Analysis Info**		Acquisition Date	5/18/2021 1:11:27 PM
Analysis Name	D:\Data\yangy\new\YANGCHENGJIE210518_4_22_01_39320.d		
Method	POS_100-1200_For LC.m	Operator	LZU
Sample Name	YANGCHENGJIE210518_4	Instrument	micrOTOF
Comment			

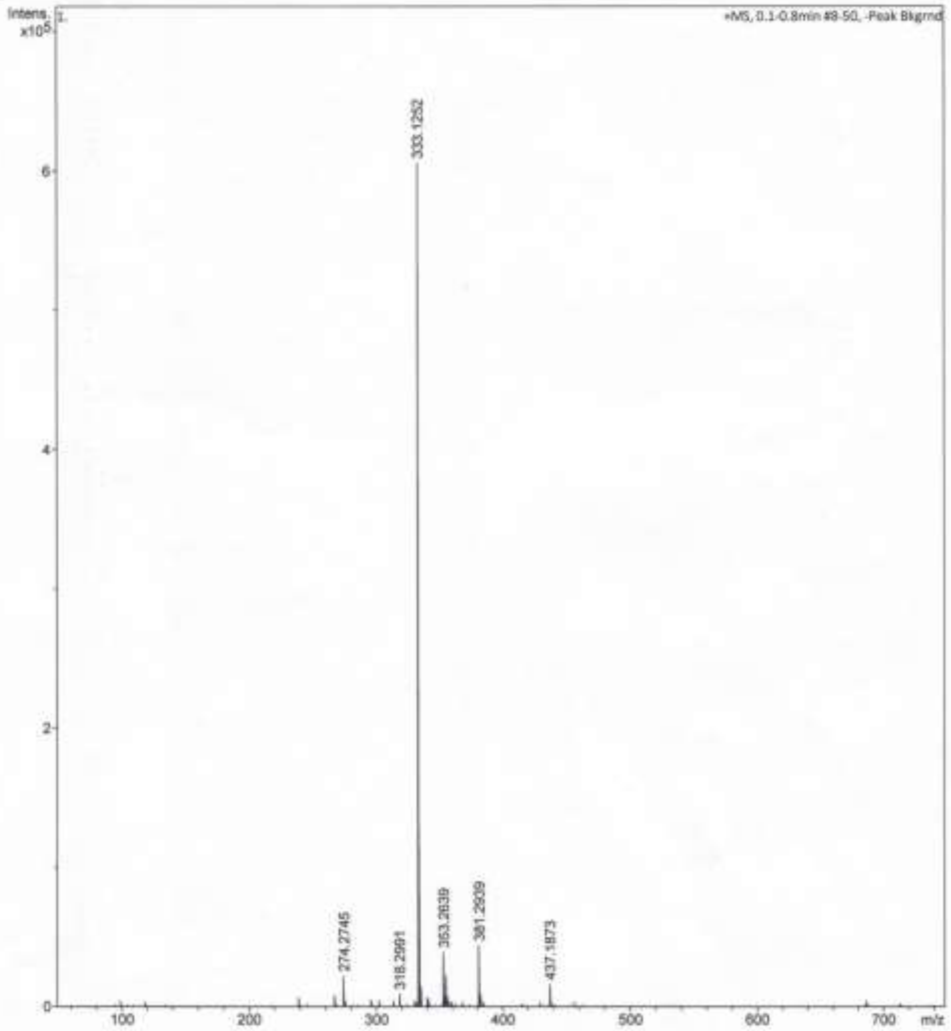

Bruker Compass DataAnalysis 4.1 printed: 5/18/2021 3:35:33 PM by: LZU Page 1 of 1

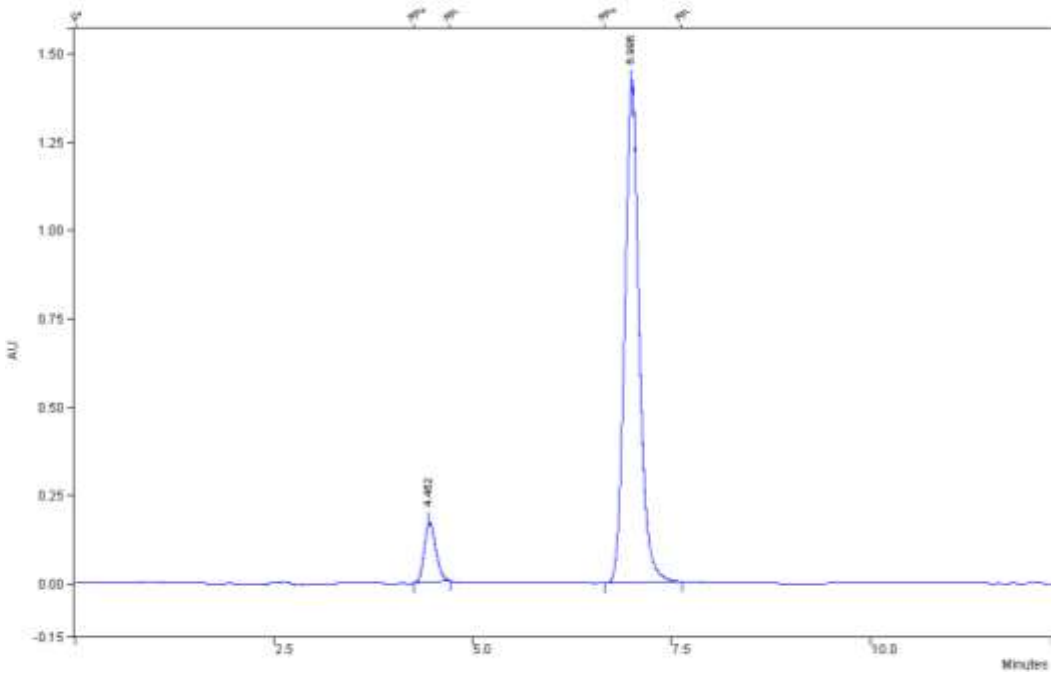

Peak No.	Peak Name	Result ()	Ret. Time (min)	Time Offset (min)	Area (counts)	Sep. Code	Width 1/2 (sec)	Status Codes
1		8.7176	4.452	0.000	17179600	BB	9.1	
2		91.2824	6.996	0.000	179889072	BB	11.2	
	Totals:	100.0000		0.000	197068672			

Figure S19. ^{1}H NMR, ^{13}C NMR, HRESIMS, HPLC spectra of compound **19**.

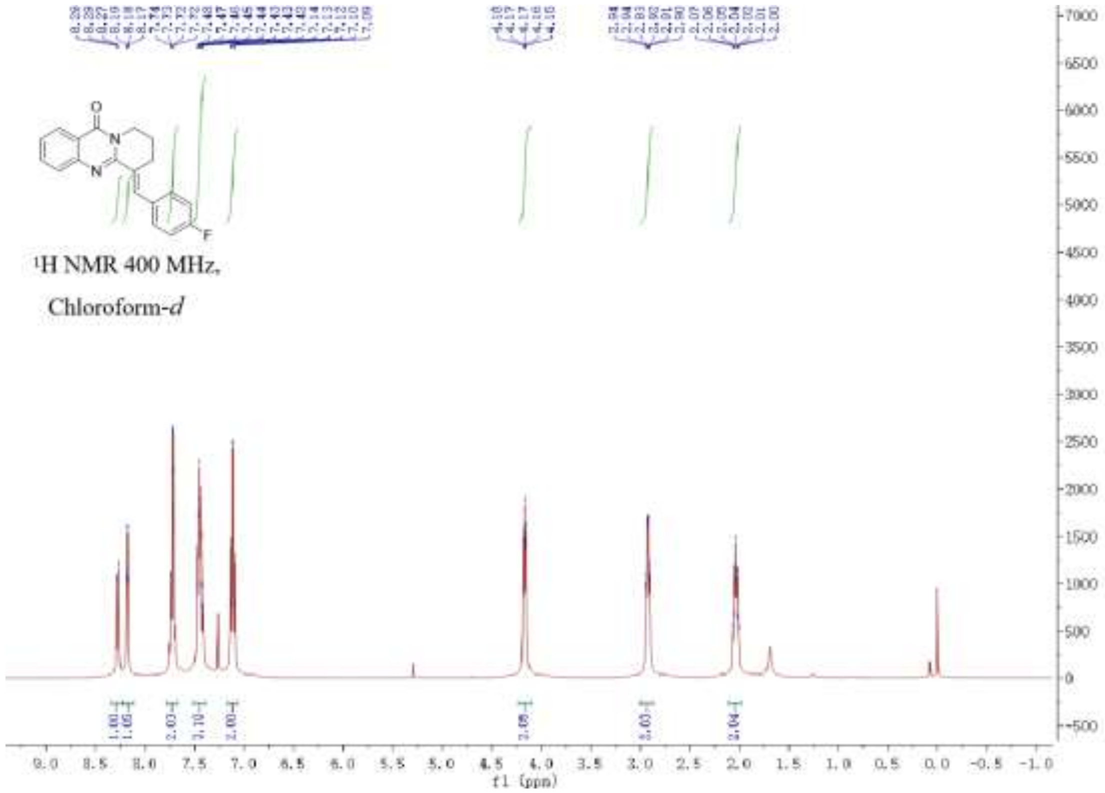

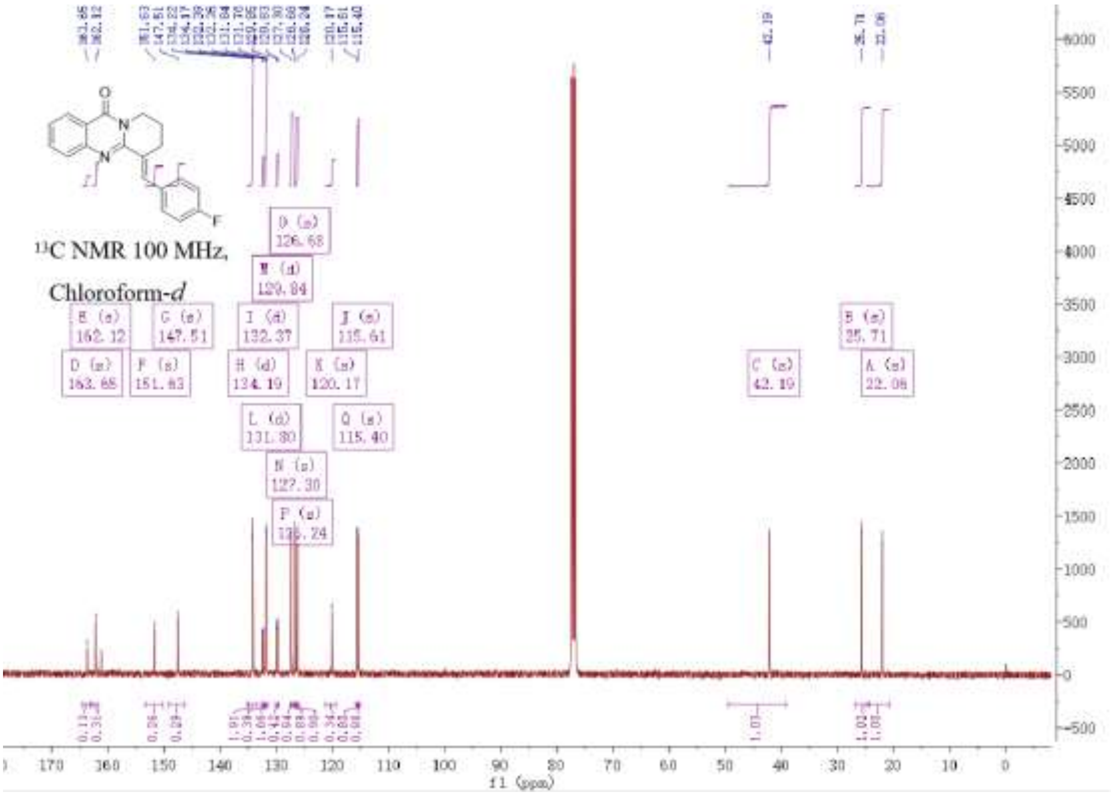

13C NMR 100 MHz,
Chloroform-d
f1 (ppm)

Generic Display Report

**Analysis Info**		Acquisition Date	5/18/2021 1:14:29 PM
Analysis Name	D:\Data\yangy\new\YANGCHENGJIE210518_5_23_01_39321.d		
Method	POS_100-1200_For LC.m	Operator	LZU
Sample Name	YANGCHENGJIE210518_5	Instrument	micrOTOF
Comment			

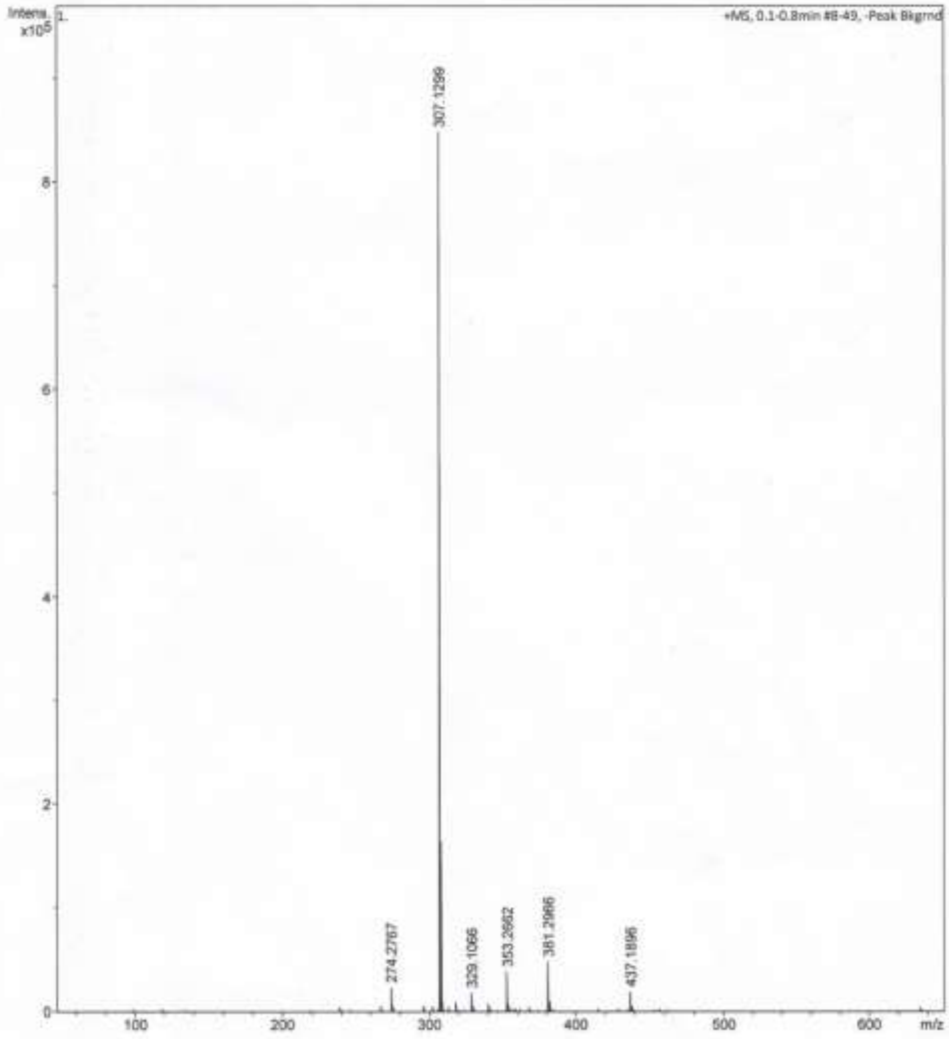

Bruker Compass DataAnalysis 4.1 printed: 5/18/2021 3:35:49 PM by: LZU Page 1 of 1

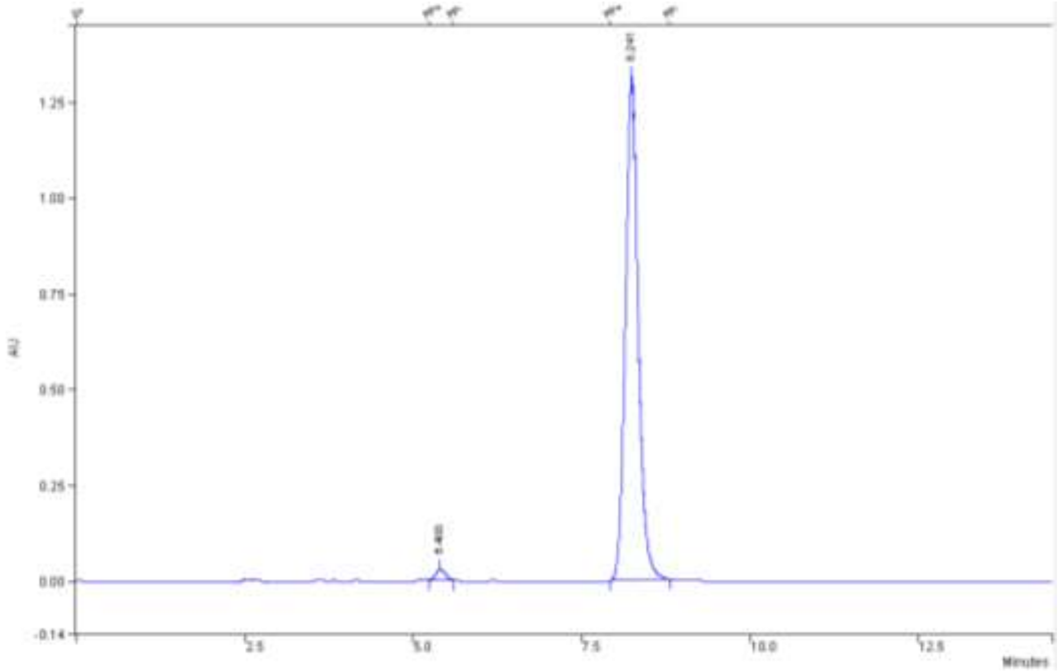

Peak No.	Peak Name	Result ()	Ret. Time (min)	Time Offset (min)	Area (counts)	Sep. Code	Width 1/2 (sec)	Status Codes
1		1.5165	5.400	0.000	2680649	BB	9.1	
2		98.4835	8.241	0.000	174090368	BB	11.8	
	Totals:	100.0000		0.000	176771017			

Figure S20. ^{1}H NMR, ^{13}C NMR, HRESIMS, HPLC spectra of compound **20**.

124

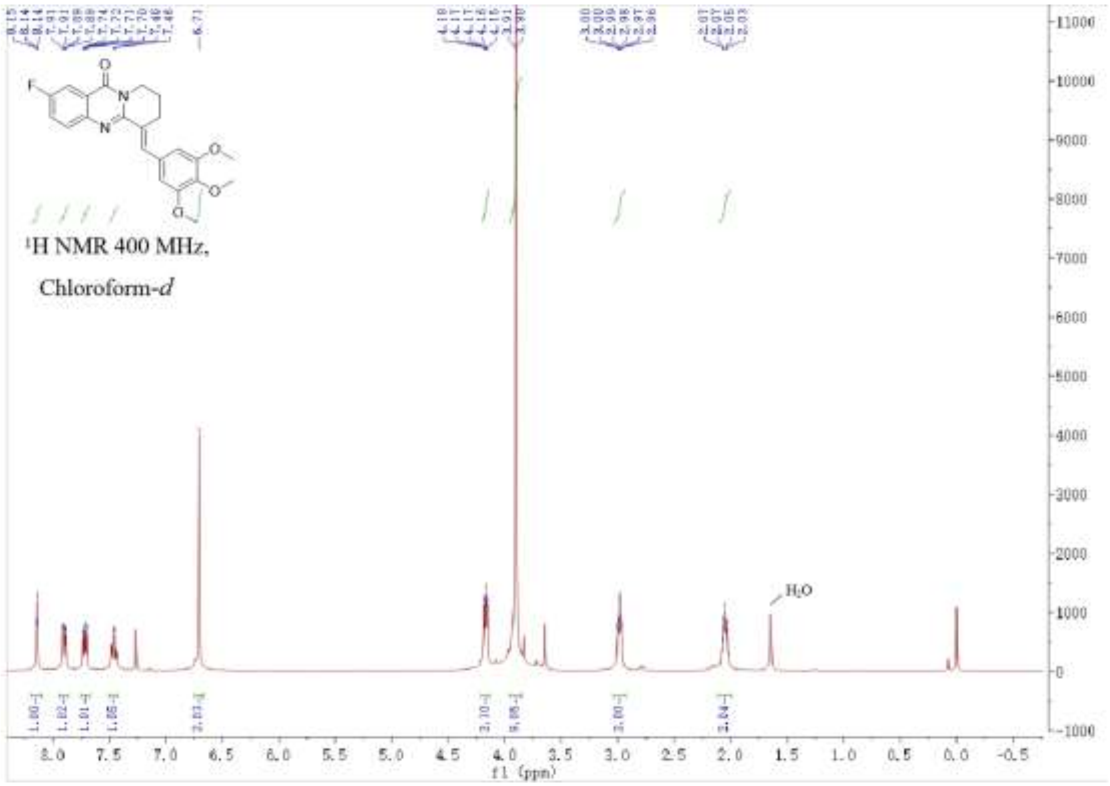

¹H NMR 400 MHz,
Chloroform-d
H₂O
f1 (ppm)

125

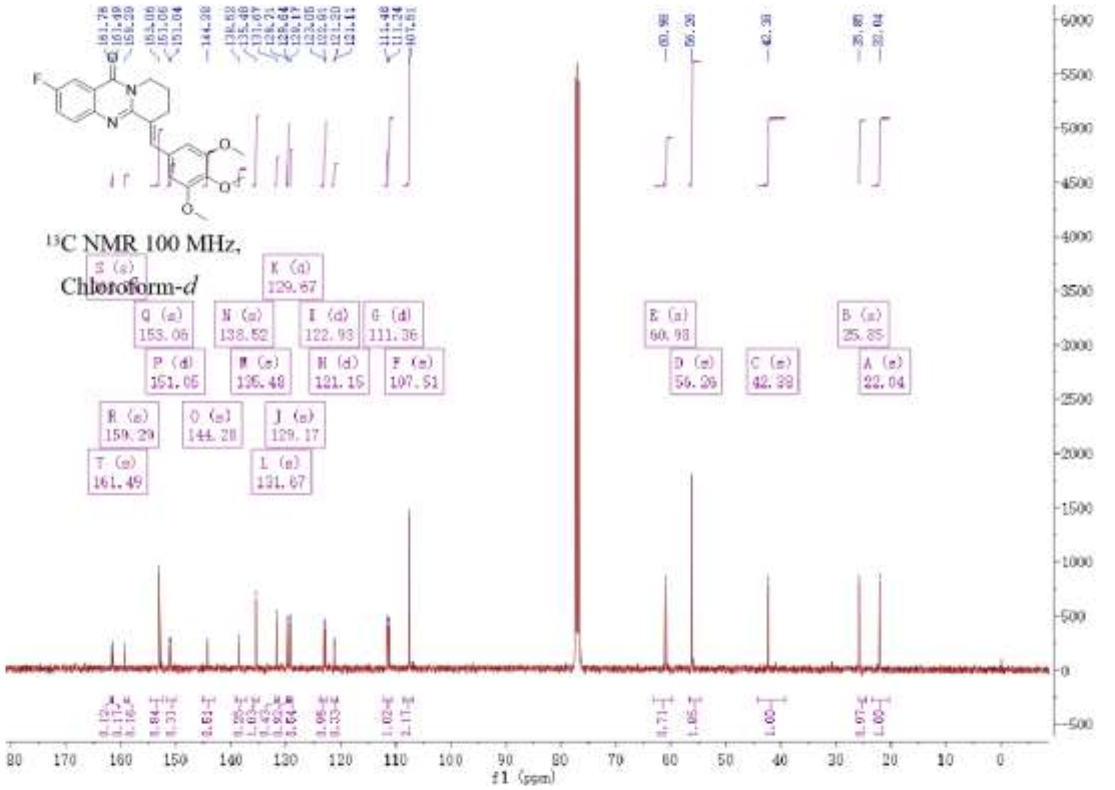

¹³C NMR 100 MHz,
Chloroform-d
f1 (ppm)

Generic Display Report

Analysis Info

Analysis Name	D:\Data\yangy\new\YANGCHENGJIE210519_2_8_01_39350.d	Acquisition Date	5/19/2021 12:07:25 PM
Method	POS_100-1200_For LC.m	Operator	LZU
Sample Name	YANGCHENGJIE210519_2	Instrument	micrOTOF
Comment			

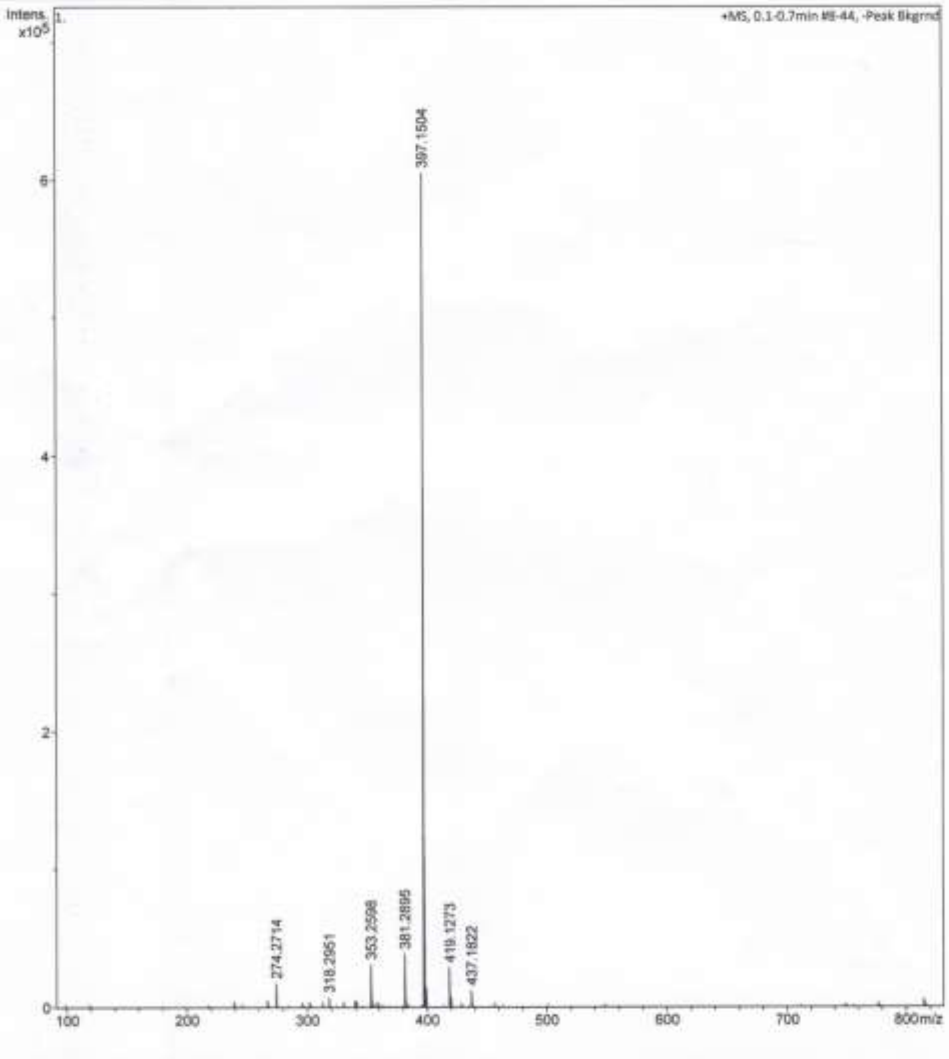

Bruker Compass DataAnalysis 4.1 printed: 5/19/2021 3:45:04 PM by: LZU Page 1 of 1

126

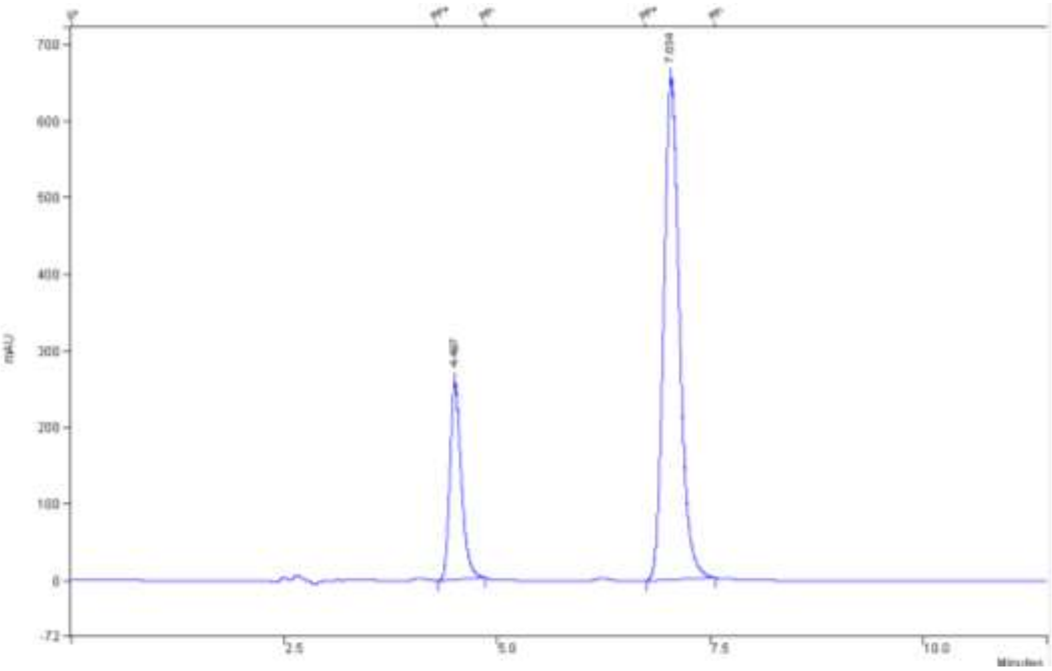

Peak No.	Peak Name	Result ()	Ret. Time (min)	Time Offset (min)	Area (counts)	Sep. Code	Width 1/2 (sec)	Status Codes
1		22.1989	4.497	0.000	24186404	BB	8.4	
2		77.8011	7.034	0.000	84766896	BB	11.7	
	Totals:	100.0000		0.000	108953300			

Figure S22. ^{1}H NMR, ^{13}C NMR, HRESIMS, HPLC spectra of compound **22**.

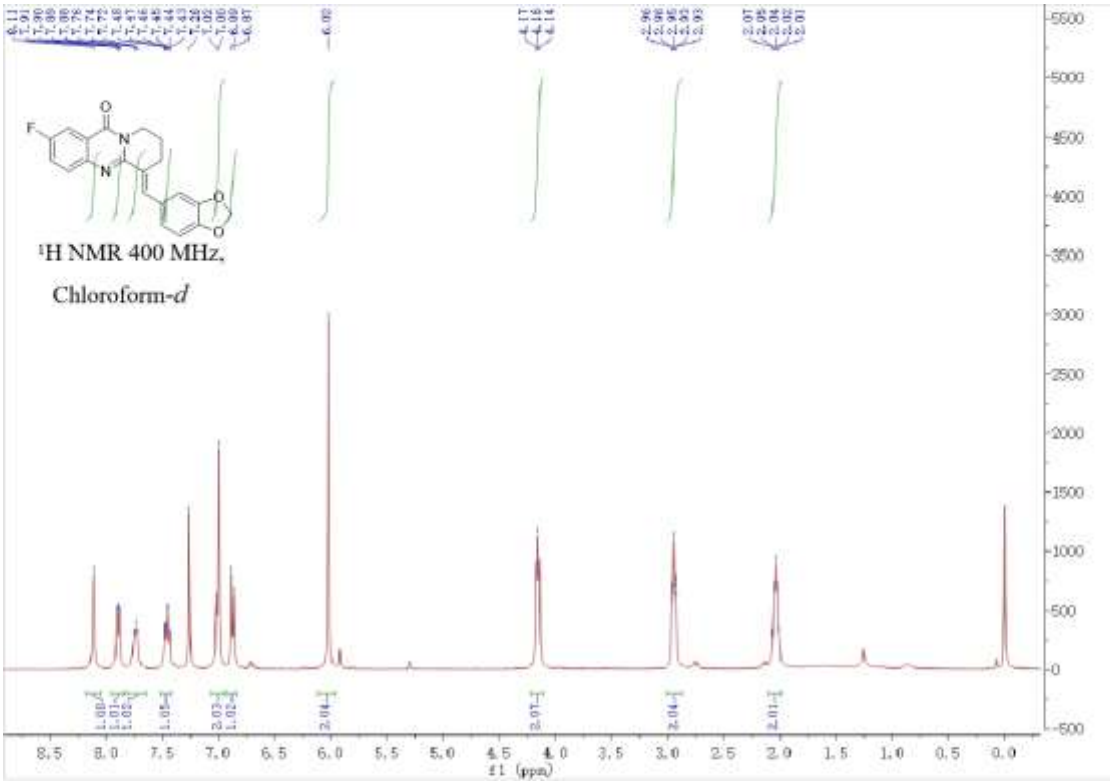

131

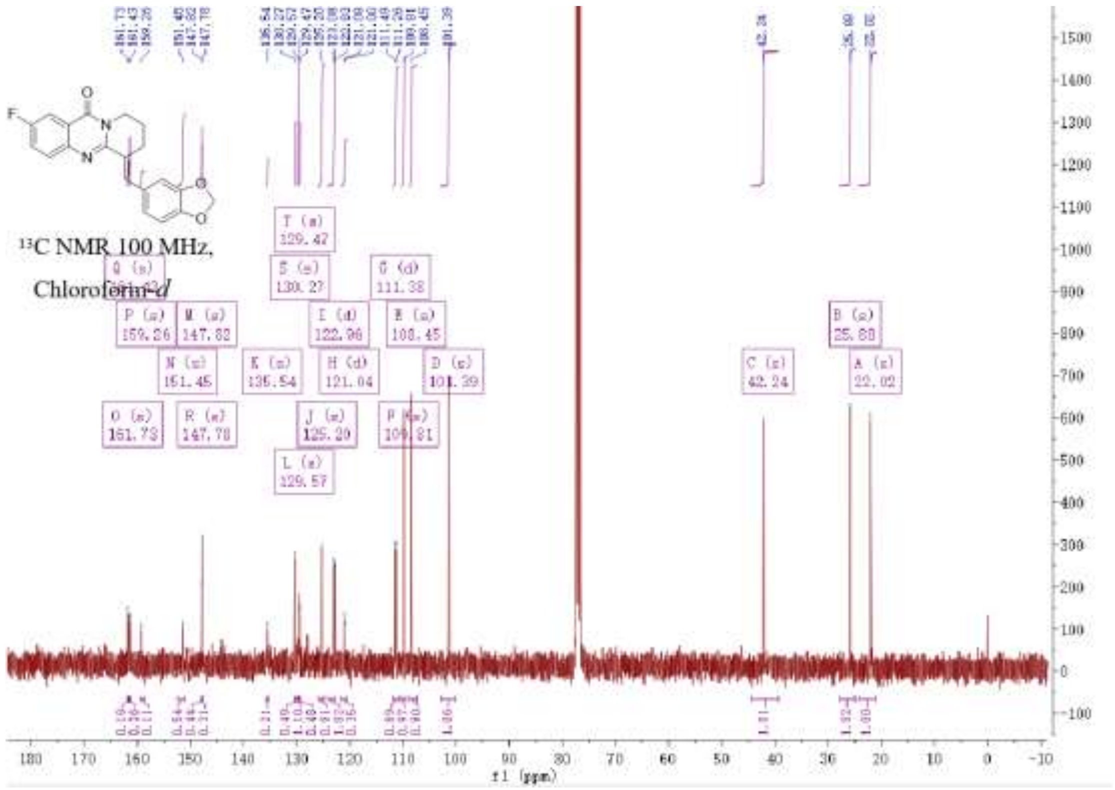

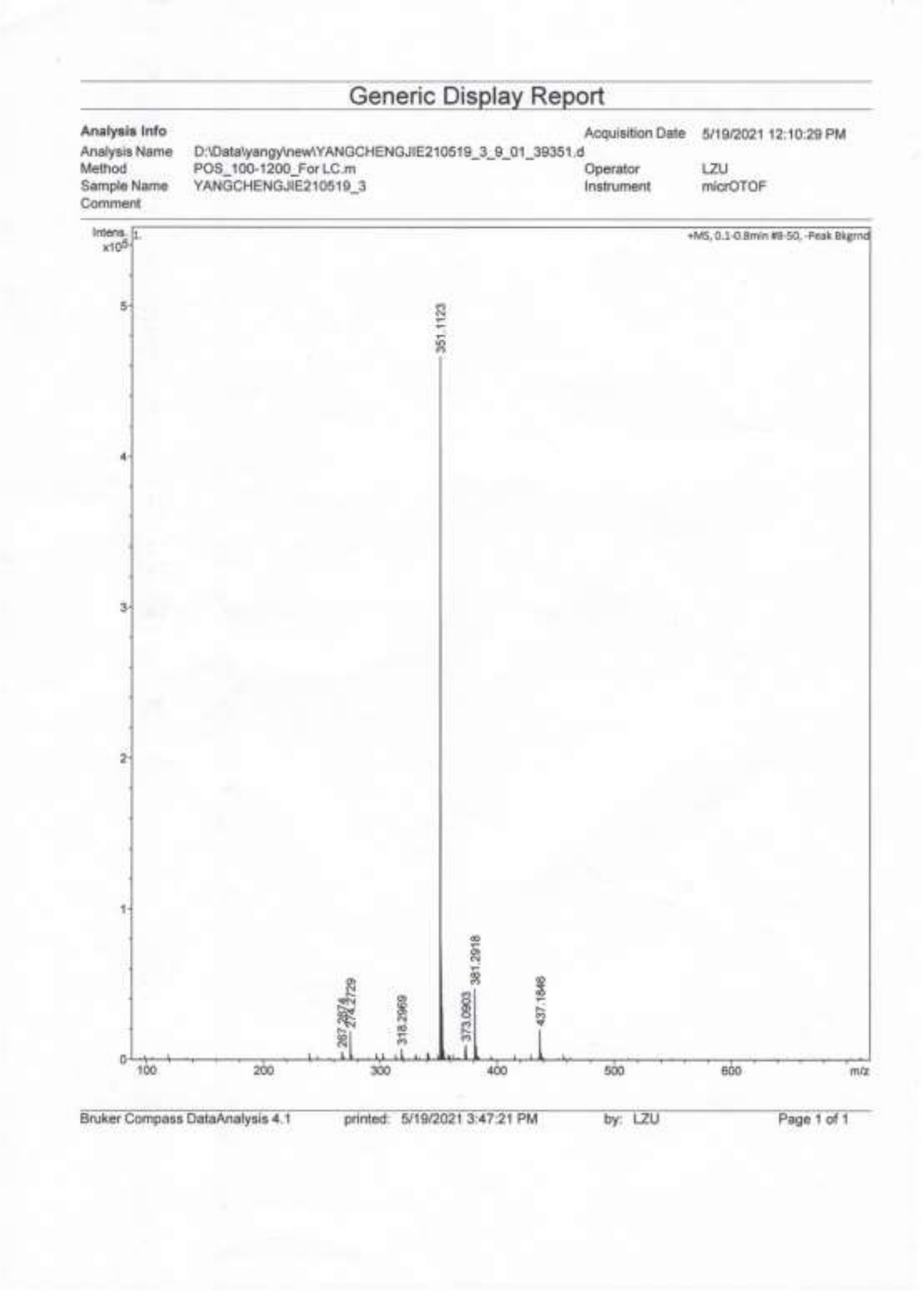

Figure S23. ^{1}H NMR, ^{13}C NMR, HRESIMS, HPLC spectra of compound **23**.

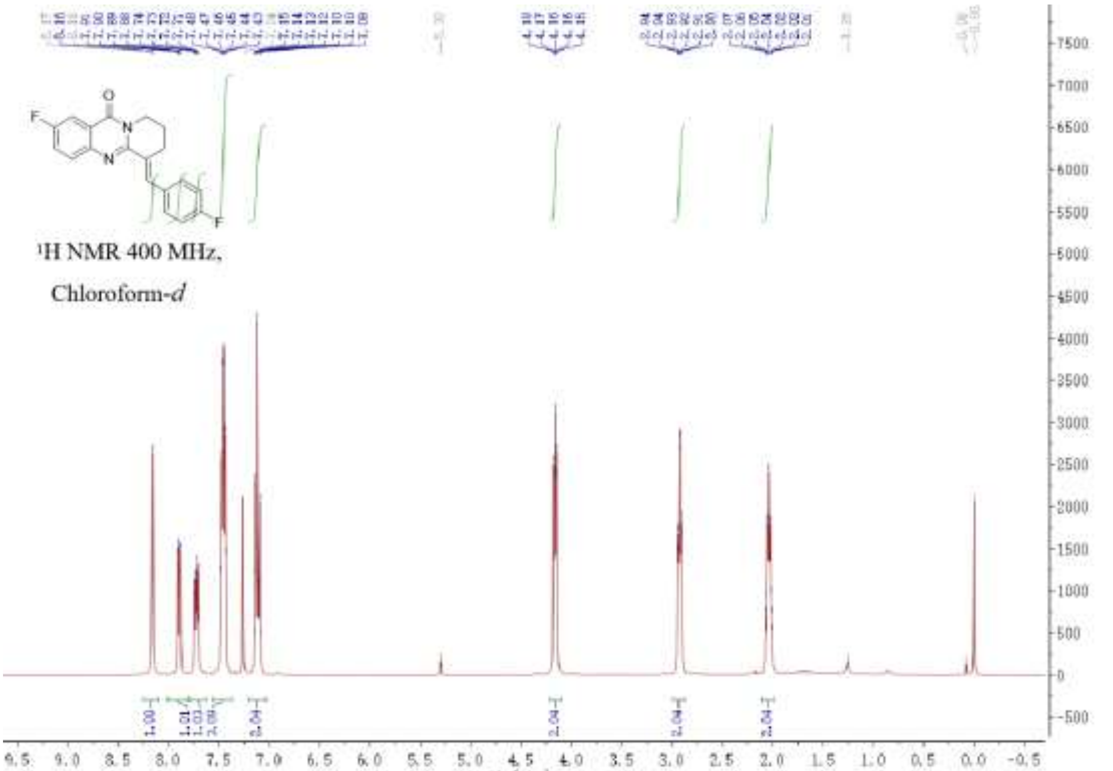
1H NMR 400 MHz,
Chloroform-d
f1 (ppm)

134

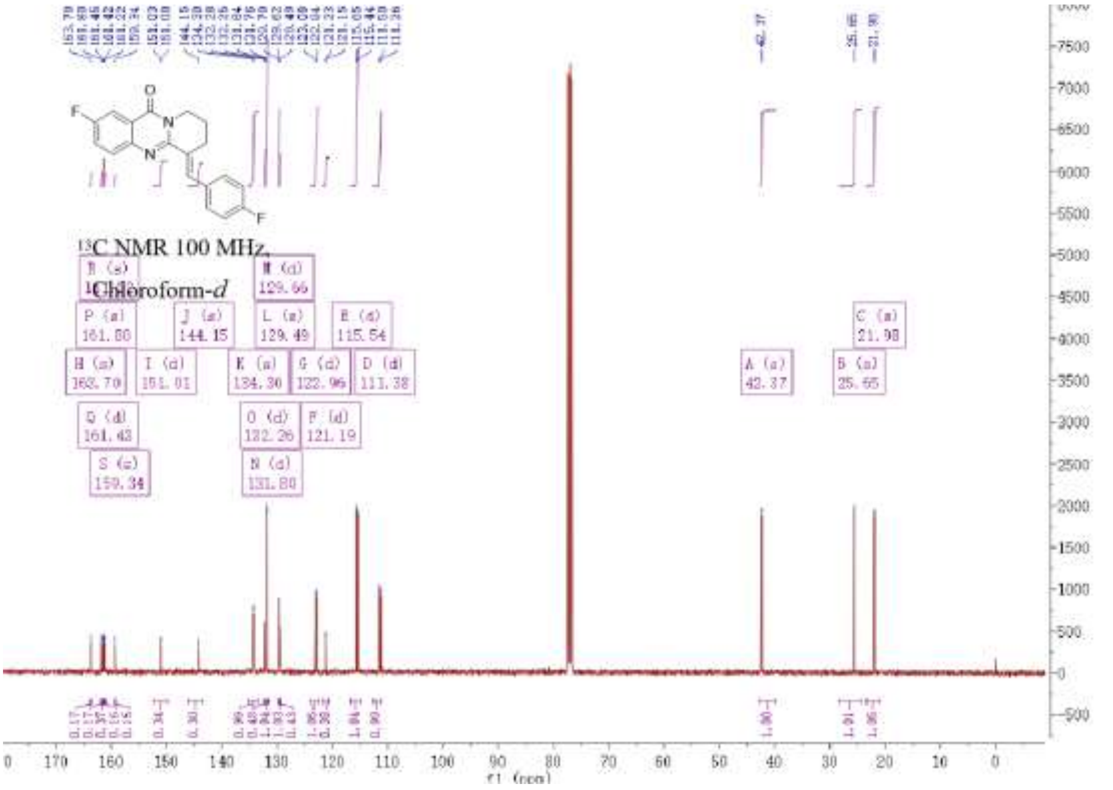
13C NMR 100 MHz,
Chloroform-d
f1 (ppm)

135

Generic Display Report

**Analysis Info**		Acquisition Date	5/19/2021 12:13:34 PM
Analysis Name	D:\Data\yangy\new\YANGCHENGJIE210519_4_10_01_39352.d		
Method	POS_100-1200_For LC.m	Operator	LZU
Sample Name	YANGCHENGJIE210519_4	Instrument	micrOTOF
Comment			

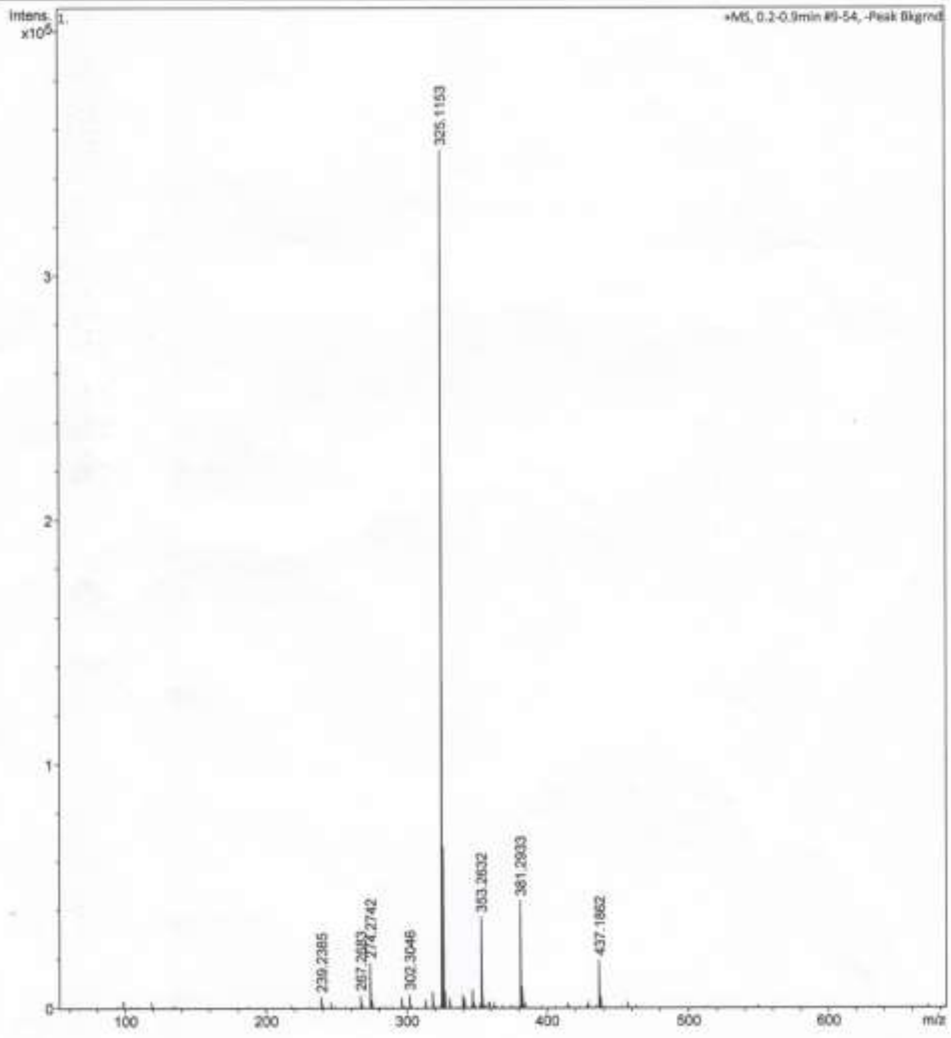

Bruker Compass DataAnalysis 4.1 printed: 5/19/2021 3:47:38 PM by: LZU Page 1 of 1

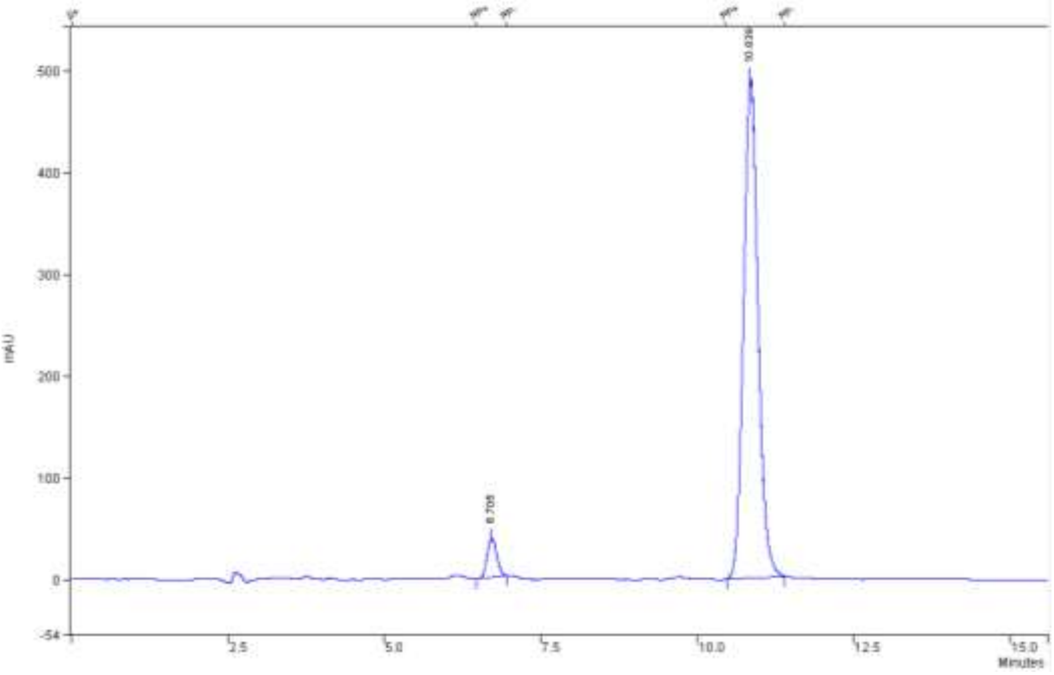

Peak No.	Peak Name	Result ()	Ret. Time (min)	Time Offset (min)	Area (counts)	Sep. Code	Width 1/2 (sec)	Status Codes
1		4.9589	6.705	0.000	4130503	BB	10.3	
2		95.0411	10.839	0.000	79164960	BB	14.7	
	Totals:	100.0000		0.000	83295463			

Figure S24. ^{1}H NMR, ^{13}C NMR, HRESIMS, HPLC spectra of compound **24**.

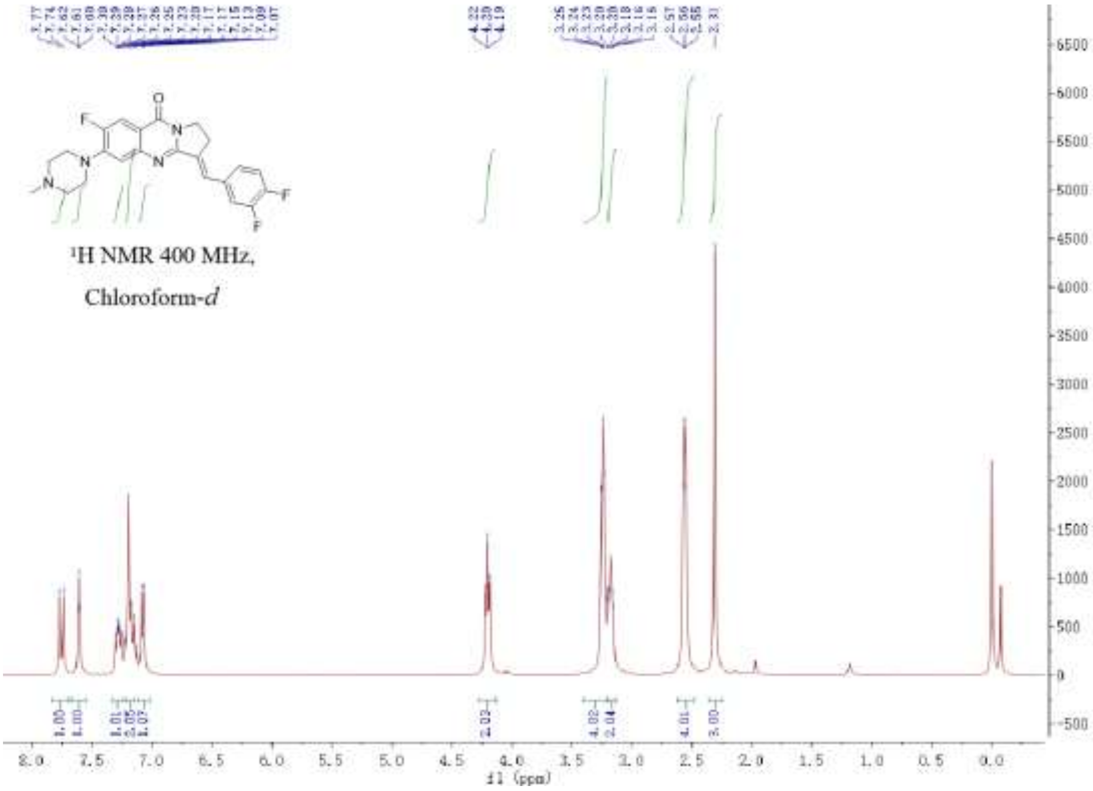

141 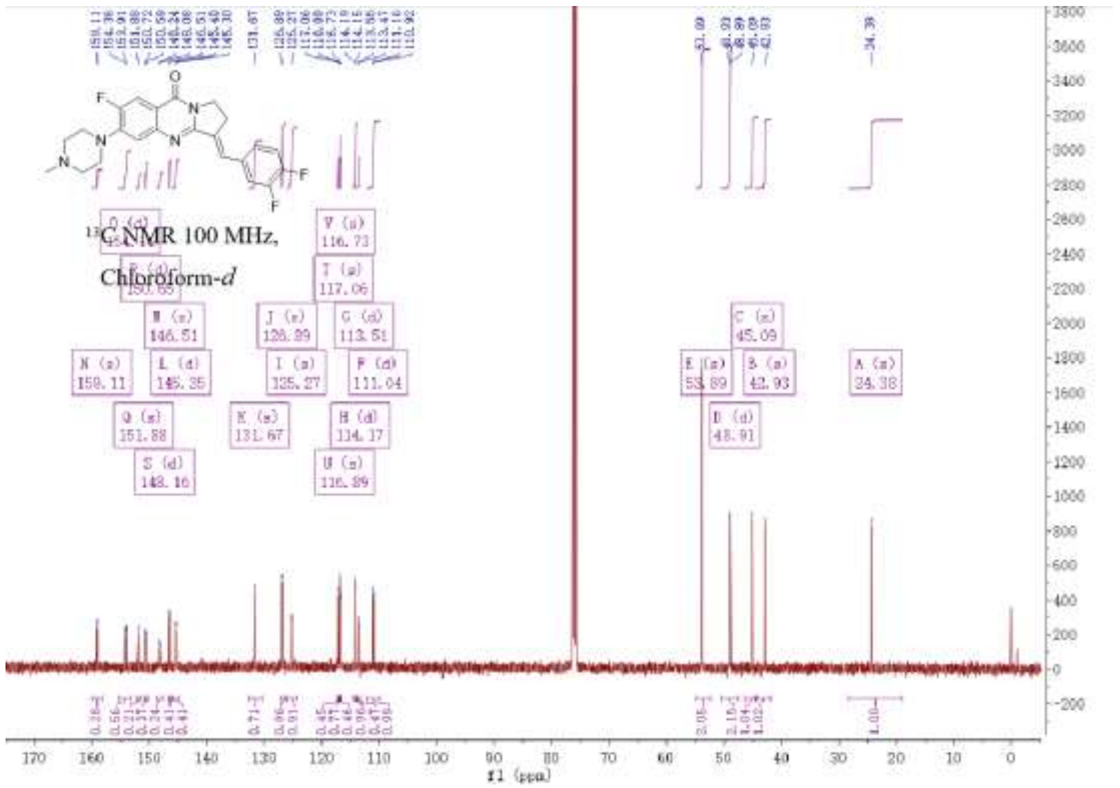
13C NMR 100 MHz,
Chloroform-d
N (s) 159.11
Q (s) 151.88
S (d) 148.16
M (s) 146.51
L (d) 145.35
K (s) 131.67
J (s) 126.89
I (s) 125.27
V (s) 116.73
T (s) 117.06
U (s) 116.89
H (d) 114.17
G (d) 113.51
F (d) 111.04
E (s) 53.89
D (d) 48.91
C (s) 45.09
B (s) 42.93
A (s) 24.38
f1 (ppm)

Generic Display Report

**Analysis Info**		Acquisition Date	5/19/2021 12:19:50 PM
Analysis Name	D:\Data\yangy\new\YANGCHENGJIE210519_6_12_01_39354.d		
Method	POS_100-1200_For LC.m	Operator	LZU
Sample Name	YANGCHENGJIE210519_6	Instrument	micrOTOF
Comment			

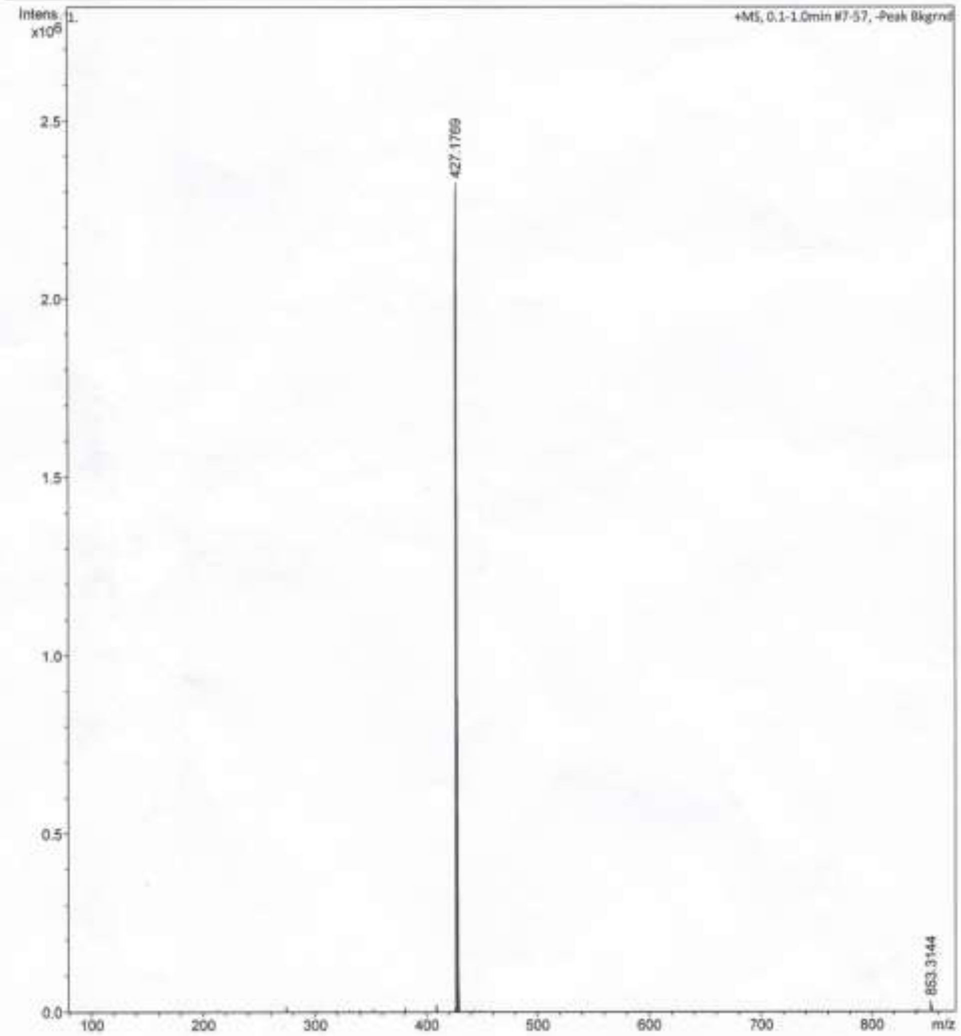

Bruker Compass DataAnalysis 4.1 printed: 5/19/2021 3:48:38 PM by: LZU Page 1 of 1

142

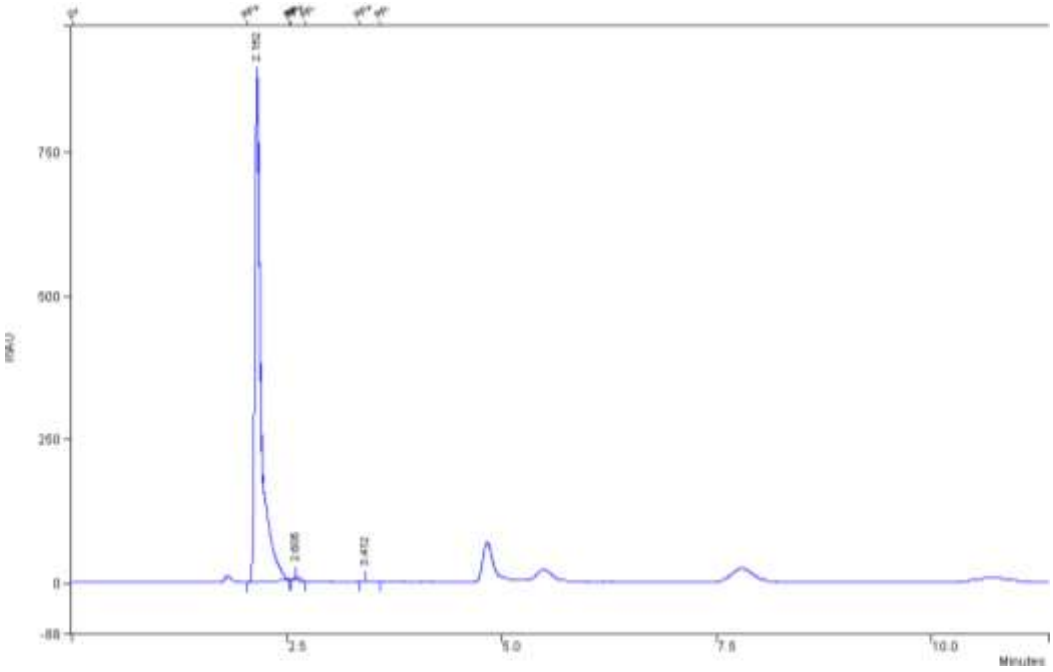

Peak No.	Peak Name	Result ()	Ret. Time (min)	Time Offset (min)	Area (counts)	Sep. Code	Width 1/2 (sec)	Status Codes
1		99.3149	2.152	0.000	45186180	BB	3.7	
2		0.5242	2.608	0.000	238494	BB	3.6	
3		0.1609	3.412	0.000	73207	BB	6.0	
	Totals:	100.0000		0.000	45497881			

Figure S25. ^{1}H NMR, ^{13}C NMR, HRESIMS, HPLC spectra of compound **25**.

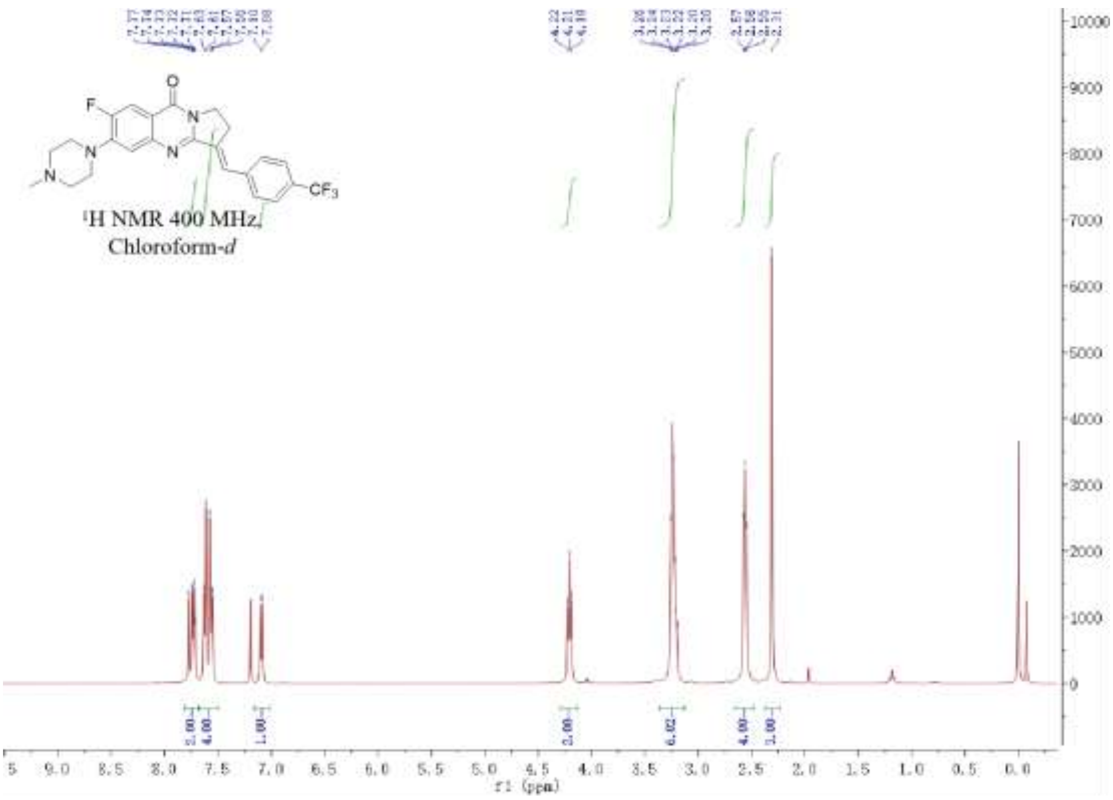
1H NMR 400 MHz,
Chloroform-d
f1 (ppm)

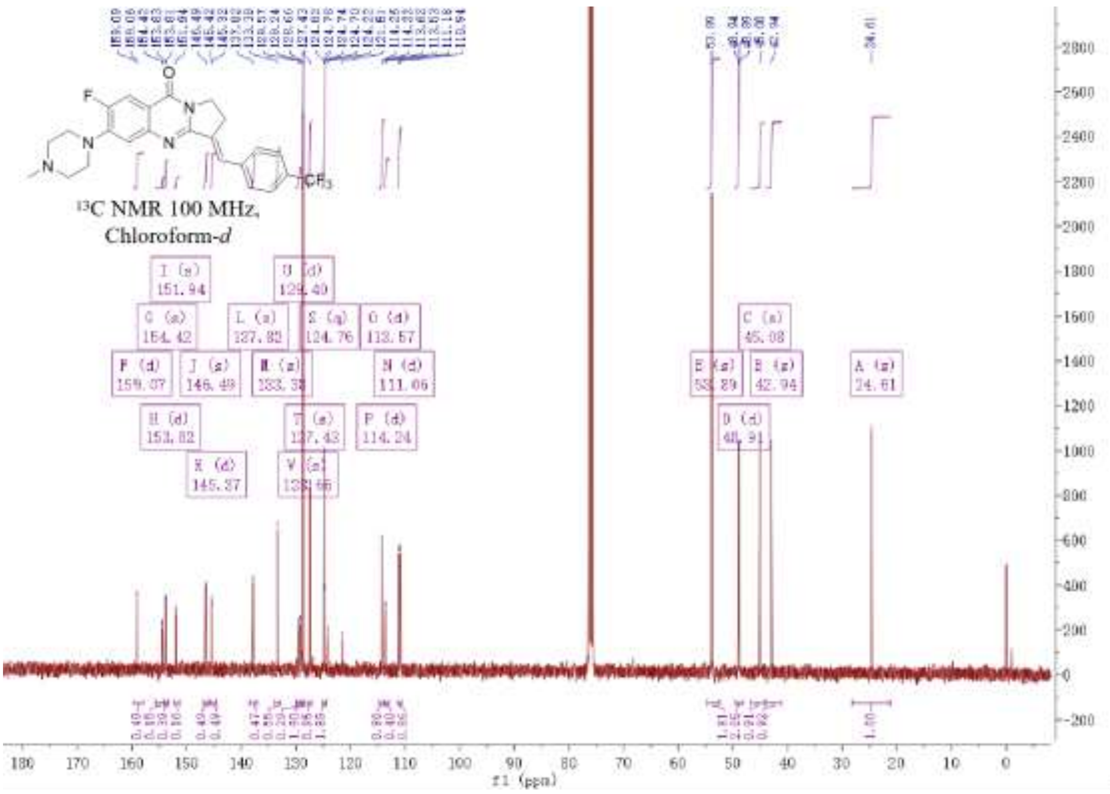
13C NMR 100 MHz,
Chloroform-d
f1 (ppm)

151

Generic Display Report

**Analysis Info**		Acquisition Date	3/11/2021 12:07:51 PM
Analysis Name	D:\Data\yangy\new\YANGCHENGJIE210311_3_01_37508.d		
Method	POS_100-1200_For LC.m	Operator	LZU
Sample Name	YANGCHENGJIE210311	Instrument	micrOTOF
Comment			

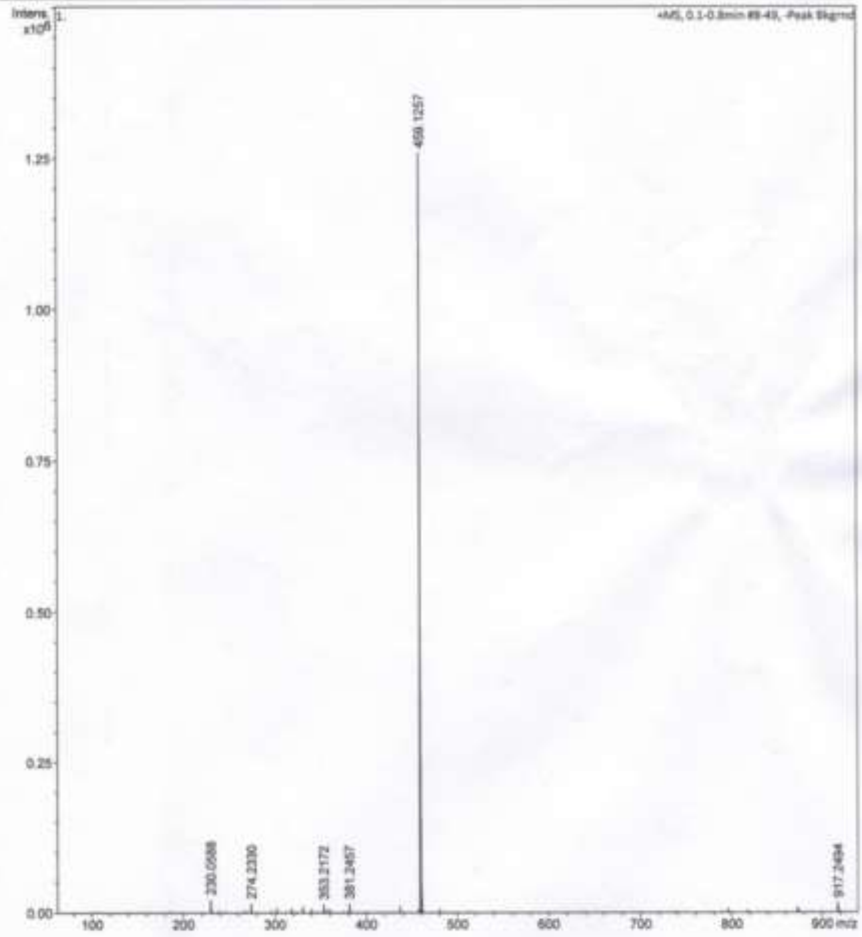

Bruker Compass DataAnalysis 4.1 printed: 3/11/2021 3:30:53 PM by: LZU Page 1 of 1

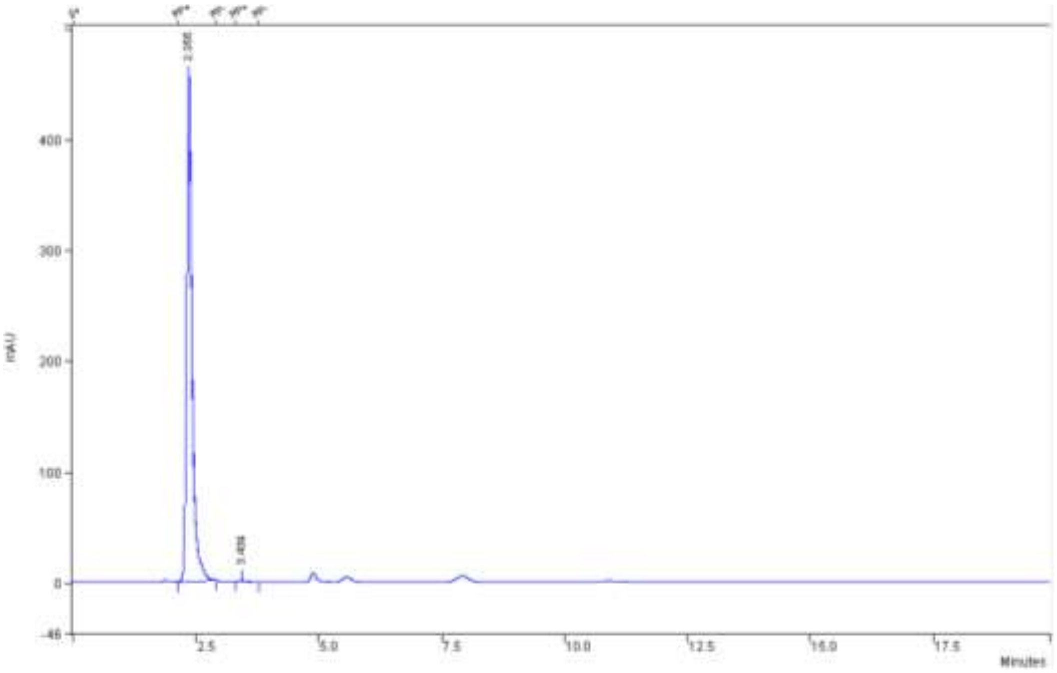

Peak No.	Peak Name	Result ()	Ret. Time (min)	Time Offset (min)	Area (counts)	Sep. Code	Width 1/2 (sec)	Status Codes
1		99.7255	2.355	0.000	34527472	BB	6.2	
2		0.2745	3.439	0.000	95025	BB	5.5	
	Totals:	100.0000		0.000	34622497			

Figure S26. ^{1}H NMR, ^{13}C NMR, HRESIMS, HPLC spectra of compound **26**.

Molecular docking analysis

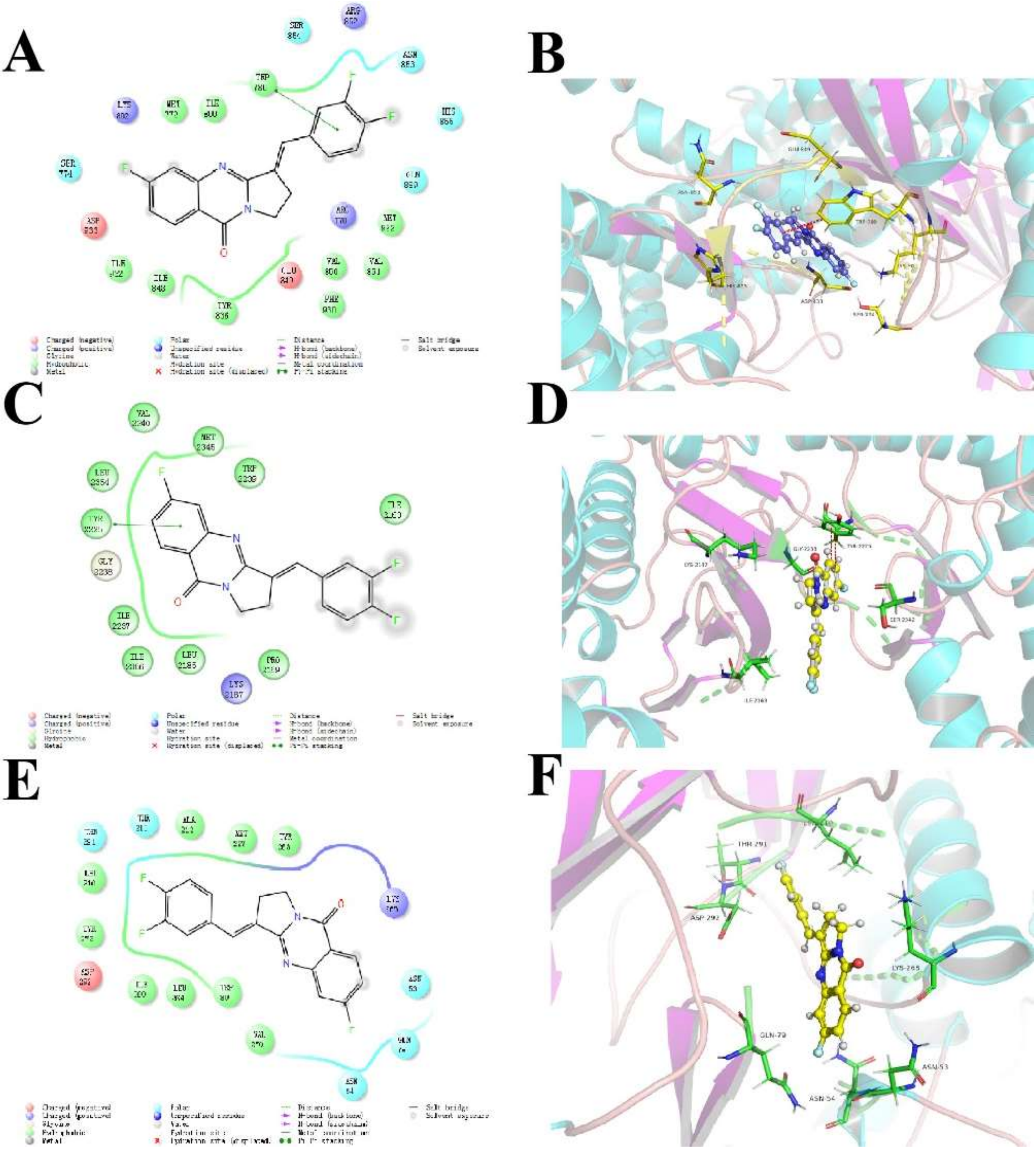

Fig. S27 The result of molecular docking between compound **6** and PI3Kα (PDB: 6gvf), mTOR (PDB: 4jt6)，AKT1 (PDB: 6HHF). (A, B) The binding conformation and the 2D binding interaction of compound **6** in the active site of PI3Kα. (C, D) The

binding conformation and the 2D binding interaction of compound 6 in the active site of mTOR. (E, F) The binding conformation and the 2D binding interaction of compound 6 in the active site of AKT1.

As shown in Table S1, compound 6 showed better affinity with AKT1. For PI3Kα protein, it can be found from the interaction diagram of the ligand that when the ligand binds to the receptor, the polar interaction is greatly increased. As a polar amino acid, aminoamide forms a polar interaction with the ligand. The basic amino acid histidine of residue 855 also forms a strong polar interaction with the ligand. (The reason may be due to the exposure of the ligand to the solvent, resulting in the inability of the residues to exchange electrons with the ligand to produce electrical interaction.) Residue 802, lysine, 770, and arginine, residue 852, are due to their It forms a positive interaction with compound **6** due to its basic alkalinity. Aspartic acid 933 and glutamic acid 849 have a negative interaction due to their own acidity. Tryptophan No. 780 forms a π-π interaction with the benzene ring of compound **6**, which increases the stability of the binding. For mTOR protein, the interaction between compound **6** and the receptor is mainly hydrophobic interaction, and some atoms of the ligand are exposed to the solvent. Among them, the 2225 residue tyrosine (TYR) can form a π-π interaction with compound **6**, which increases the stability of the system binding. Lysine (LYS), residue No. 2187, is a basic amino acid, which can be positively charged in the solvent to form a positive interaction with the ligand. Glycine No. 2238 (GLY) is because of its own hydrophobicity, which can form a strong hydrophobic interaction with the ligand. For AKT1 protein, the carboxyl anion on aspartic acid 292 formed a negative interaction

with the ligand. Lysine 268 formed a positive interaction with the ligand due to its amino cation. Threonine 211 and Threonine 291, Asparagine 53 and Asparagine 54, and Glutamine 79 as polar amino acids formed strong polar interactions with the ligand.

Table S1. Binding affinities of compound 6 and PI3K, AKT and mTOR proteins.

Protein	PDB ID	Resolution (Å)	Source	Ligand scoring
PI3K	6gvf	2.50	Home sapiens	-7.083
AKT	6hhf	2.90	Home sapiens	-8.869
mTOR	4jt6	3.60	Home sapiens	-7.608
